# The allelopathic, adhesive, hydrophobic and toxic latex of *Euphorbia* species is the cause of fairy circles investigated at several locations in Namibia

**DOI:** 10.1186/s12898-020-00313-7

**Published:** 2020-08-03

**Authors:** J. J. Marion Meyer, Christiaan E. Schutte, Jan W. Hurter, Nicole S. Galt, Petunia Degashu, Greg Breetzke, Denis Baranenko, Nicole L. Meyer

**Affiliations:** 1 Department of Plant and Soil Sciences, University of Pretoria, Pretoria, 0002 South Africa; 2 Department of Geography, Geoinformatics and Meteorology, University of Pretoria, Pretoria, 0002 South Africa; 3 ITMO University, 9 Lomonosov Street, St Petersburg, 191002 Russia

**Keywords:** Fairy circles, Namibia, *Euphorbia*, Latex, Allelopathy, Spatial pattern

## Abstract

**Background:**

In this multidisciplinary study we present soil chemical, phytochemical and GIS spatial patterning evidence that fairy circles studied in three separate locations of Namibia may be caused by *Euphorbia* species.

**Results:**

We show that matrix sand coated with *E. damarana* latex resulted in faster water-infiltration rates. GC-MS analyses revealed that soil from fairy circles and from under decomposing *E. damarana* plants are very similar in phytochemistry. *E. damarana* and *E. gummifera* extracts have a detrimental effect on bacteria isolated from the rhizosphere of *Stipagrostis uniplumis* and inhibit grass seed germination. Several compounds previously identified with antimicrobial and phytotoxic activity were also identified in *E. gummifera.* GIS analyses showed that perimeter sizes and spatial characteristics (Voronoi tessellations, distance to nearest neighbour ratio, pair correlation function and L-function) of fairy circles are similar to those of fairy circles co-occurring with *E. damarana* (northern Namibia), and with *E.* *gummifera* (southern Namibia). Historical aerial imagery showed that in a population of 406 *E.* *gummifera* plants, 134 were replaced by fairy circles over a 50-year period. And finally, by integrating rainfall, altitude and landcover in a GIS-based site suitability model, we predict where fairy circles should occur. The model largely agreed with the distribution of three *Euphorbia* species and resulted in the discovery of new locations of fairy circles, in the far southeast of Namibia and part of the Kalahari Desert of South Africa.

**Conclusions:**

It is proposed that the allelopathic, adhesive, hydrophobic and toxic latex of *E. damarana, E.* *gummifera*, and possibly other species like *E. gregaria,* is the cause of the fairy circles of Namibia in the areas investigated and possibly in all other areas as well.

## Background

The fairy circles (FCs) of the arid grasslands in the pro-Namib Desert have puzzled the scientific community for decades. Within these ephemeral grasslands are hundreds of thousands of regularly spaced circular patches not containing any vegetation cover and are commonly referred to as FCs [1].

Past research has indicated that the FCs are confined to a narrow strip, about 50–100 km inland from the Atlantic Ocean, which stretches down from southwestern Angola, through Namibia to northwestern South Africa [2, 3]. An empirical model to predict the occurrence of FCs showed that they are a climate dependent emergent phenomenon [4].

One of the striking characteristics of FCs are that the soil moisture content inside the circles are considerably higher than outside FCs, especially from levels 50 cm below the soil surface [4–8]. The volumetric soil water content of FCs has been found to be in excess of 53 mm of water stored in the upper 100 cm of the soil (even throughout the dry seasons) [2]. Another significant characteristic of FCs are their faster water infiltration rates [3, 9]. These characteristics have previously been explained by the possible trapping of aeolian and water-borne sediments by plants that could result in soil textural changes beneath the vegetation, which in turn, explains the heterogeneity in hydrological processes such as infiltration and runoff [9].

Recent attempts to explain the formation of FCs have focussed on mathematical models to determine the underlying hydraulic processes that gave rise to the regular patterning of FCs, especially in areas like Namibrand and Giribes Plain in Namibia [10, 11]. It was argued that FCs are self-organised vegetation patterns that emerge from positive biomass-water feedbacks [10].

Despite a plethora of research investigating termite activity [2], soil chemistry [12] soil hydraulics [10] and other inherent FC properties [1], there are still a number of different theories attempting to explain their origin and maintenance.

**Fig. 1:**
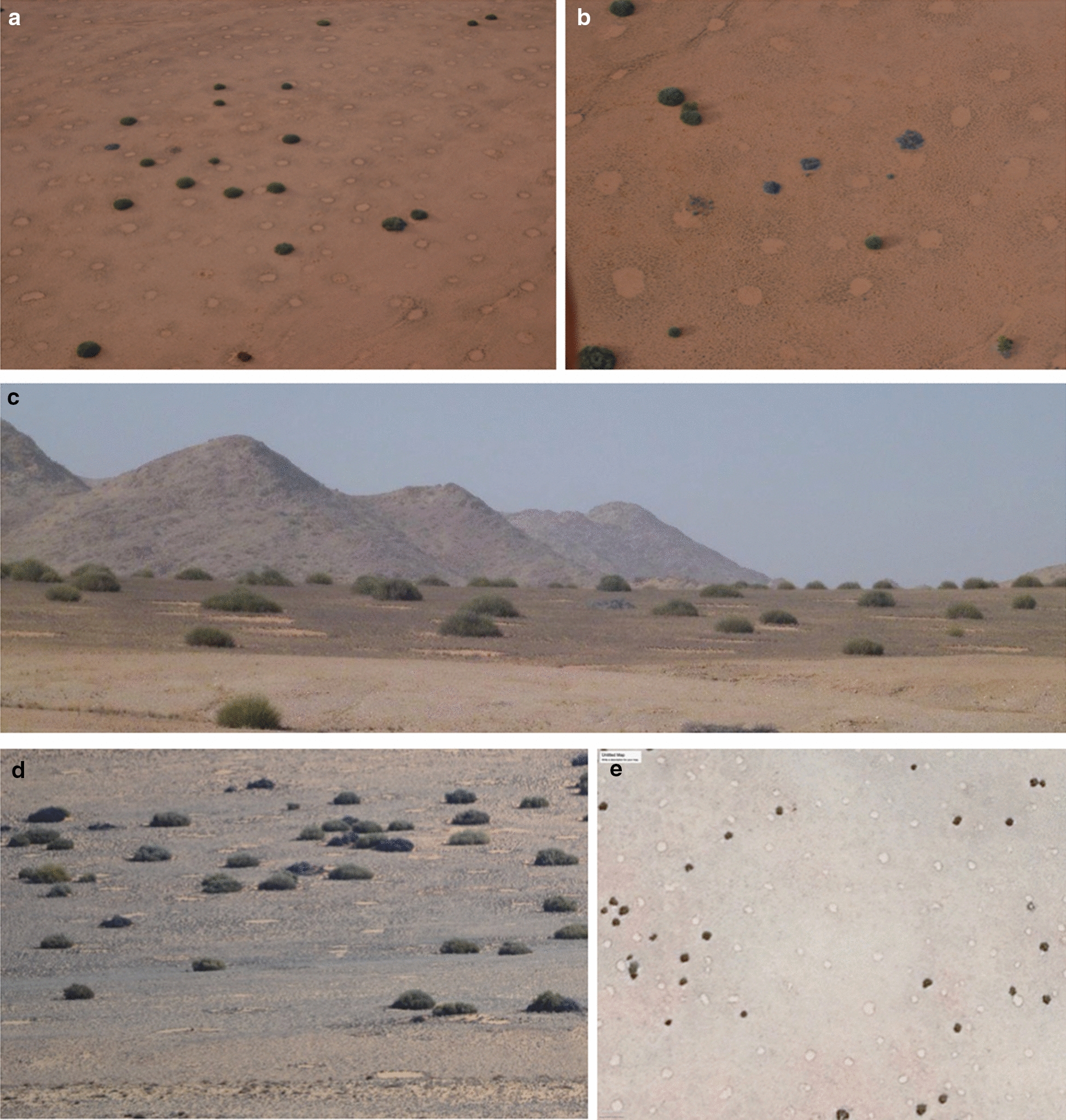
*E. damarana* co-occurring with FCs at Brandberg (**a**, **b**), Giribes Plain (**c**), and *E. gummifera* at Garub near Aus (**d**) and the same area in a Google Earth™ image (**e**)

**Fig. 2:**
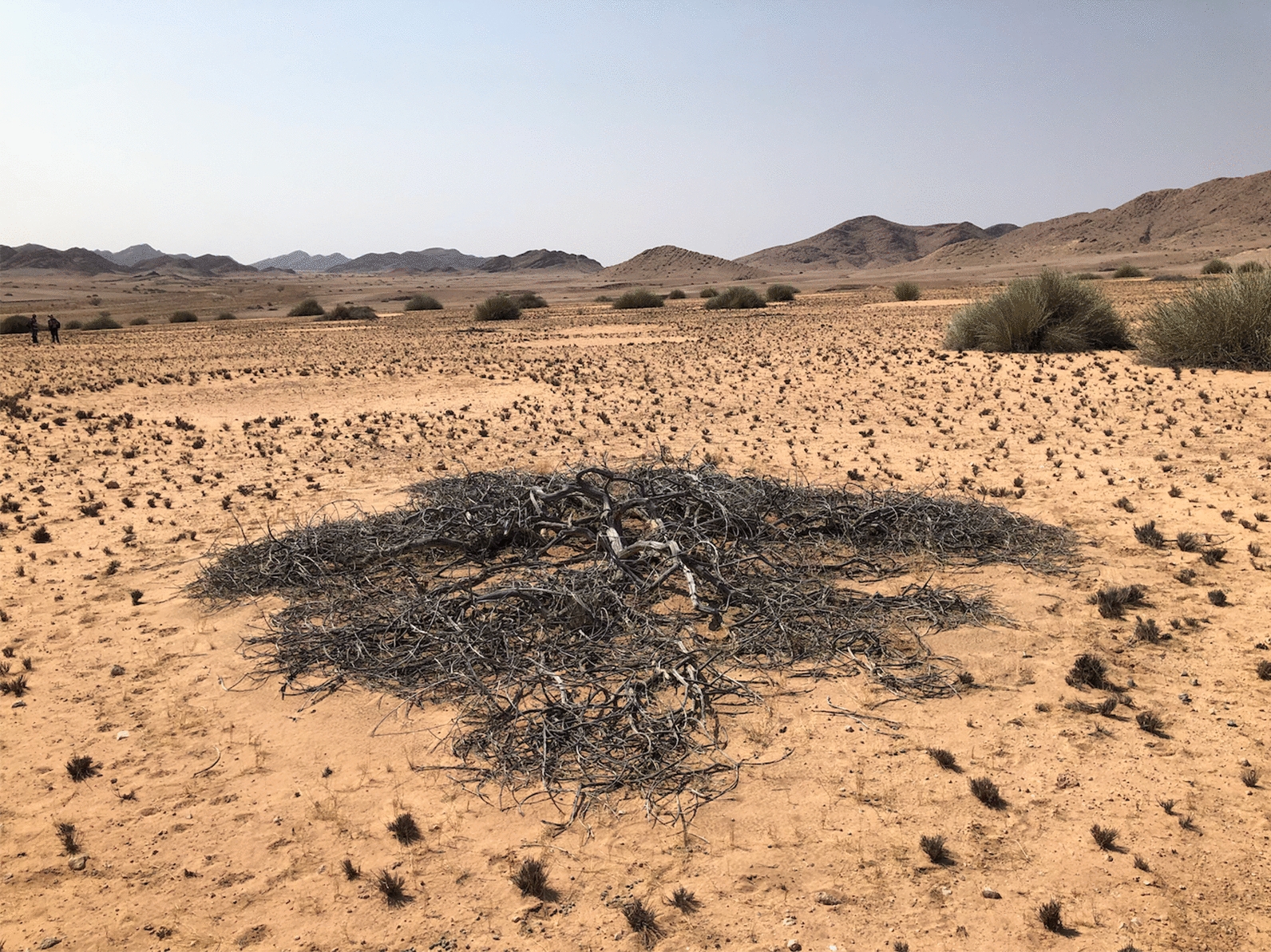
*E. damarana* and FCs in the background and the dead plant remains in the front described by Theron [13] on the Giribes Plain. The outline of a new FC can be seen and several more like these are present at this site

The allelopathy theory proposes that FCs are caused by various *Euphorbia* spp. (roundish, highly poisonous shrubs containing latex) which upon dying leave compounds behind in the soil that inhibit the growth of vegetation leading to barren circles [12, 13]. Fairy circles have been observed to co-occur with various *Euphorbia* spp. (Fig. 1) including *E. damarana* L.C.Leach (Giribes and Brandberg, northern Namibia), *E. gummifera* Boiss. (Garub, southern Namibia) and *E. gregaria* Marloth (southwest of Keetmanshoop, Namibia) [12, 14]. The first documented co-occurrence of FCs and *E. damarana* was on the Giribes Plain by Theron [13], where large numbers of dead *E. damarana* plants were also found. Some of these dead and decaying *E. damarana* sites were marked during 1978 [13] and were still not covered by grasses in 2016 [14], although more than two metres of rain was recorded in this area during the last 40 years [2]. These *Euphorbia* decaying sites resemble emerging FCs (Fig. 2). Ten FCs were also marked in 1978 [13] which were confirmed to be intact circles four decades later [1, 12, 14], suggesting that FCs do not appear/disappear in new locations after several years of rainfall and droughts, but are rather stable entities. However, publications supporting the vegetation self-arrangement theory [3, 9, 15] state that they appear in different places during dry conditions and then disappear after good rainfall. Tschinkel [15] found the life span of FCs to be 43‒75 years.

In the Garub area evidence was found that euphol, a characteristic triterpenoid from the *Euphorbia* genus, was present in soil from inside FCs but not in the matrix soil outside the FCs [12]. A correlation in the sizes of FCs and *E.* *gummifera* was also shown [12], as was done with FCs and *E. damarana* on the Giribes Plain [13].

Proponents of the vegetation self-arrangement theory [10] recently argued that while FCs in Garub may be caused by *E.* *gummifera* [16], this can not explain the origin of FCs in other parts of the Namib where *Euphorbia* spp. are absent.

We report in this multidisciplinary study on the effect of *Euphorbia* spp. on FC soil chemistry and hydraulics, and on their germination inhibition and antibacterial activity on rhizosphere bacteria. We also wanted to determine if these *Euphorbia* spp. could give rise to the current spatial characteristics of FCs and therefore compared their current spatial patterning with those of FCs in four areas of Namibia by analysing Voronoi tessellations, distance to nearest neighbour (nn) ratio, the pair correlation function, the L-function, as well as perimeter size. Historical and recent imagery of these *Euphorbia* spp. were compared to determine if they formed FCs after they died. Lastly, taking the above and published results into consideration, we predicted where FCs could occur and determined if this correlated with the distribution of three succulent *Euphorbia* spp.

## Results

### Physical and chemical properties of FC soil

The aim of this section was to determine if the soil of FCs in areas where euphorbias co-occur with FCs (mixed sites) was as sandy as previously reported in FC hotspots. In order to determine the mechanism of FC formation the soil’s hydrophobicity and infiltration capabilities were investigated. GC-MS analyses were performed on soil from FCs and the matrix, as well as on soil collected from underneath dead euphorbias, to compare the similarity in compounds/fragments of remaining compounds (e.g. long chain hydrocarbons) in these soil types.

#### Physical properties, wettability and infiltration time of soil

**Fig. 3:**
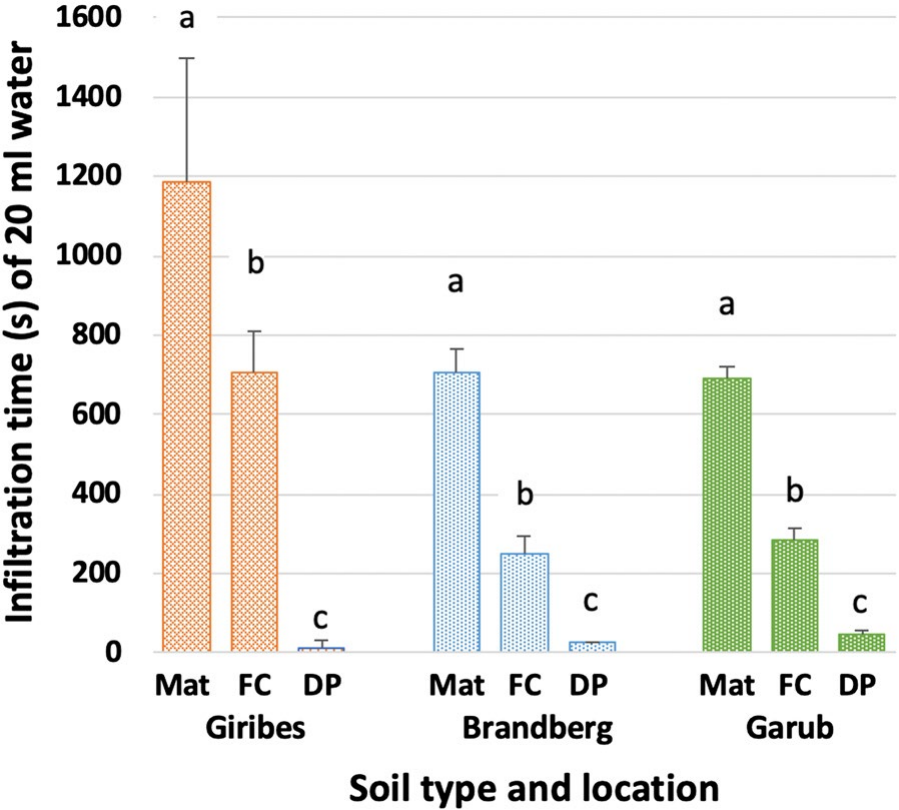
Infiltration time (s) of 20 ml water in matrix (Mat), FC, and DP soil from Giribes, Brandberg and Garub (P < 0.05, soil types compared only inside locations)

FC soil was classified as 85‒90% sand at Giribes Plain, Brandberg and Garub (see Additional file 1: Text S5, Fig. S1). The soil water infiltration time decreased significantly (p < 0.001) (Fig. 3) from the matrix, to the FC, to soil from under dead *Euphorbia* plants (DPs) at all three locations. The matrix sand coated with 5% or more *E. damarana* latex decreased the infiltration time significantly [infiltration time 16.0 ± 1.0 s (SD)] rendering the soil extremely hydrophobic.

The soil droplet wettability results showed that all soil samples from the matrix in Giribes, Brandberg and Garub were hydrophilic, whereas 75% of FC samples were hydrophobic and nearly all from under dead euphorbias were extremely hydrophobic (see Additional file 1: Table S1, Fig. S1 and Additional file 2: Movie S1).

The hydrophobicity (see Additional file 1: Table S1) and faster infiltration time (Fig. 3), and thus also the infiltration rate (of 20 ml water), in FC soil will partly lead to runoff towards the periphery of the FC and the remaining rainwater will flow through preferential soil channels (previous stem and root activity) [17, 18] resulting in wetter soil conditions at deeper levels (see Discussion; Physical and chemical properties of soil) [2, 3, 5, 6]. Hydrophobic properties and faster infiltration rates could be introduced to matrix soil by coating it with *Euphorbia* latex.

#### GC-MS analysis of soil extractions

**Fig. 4:**
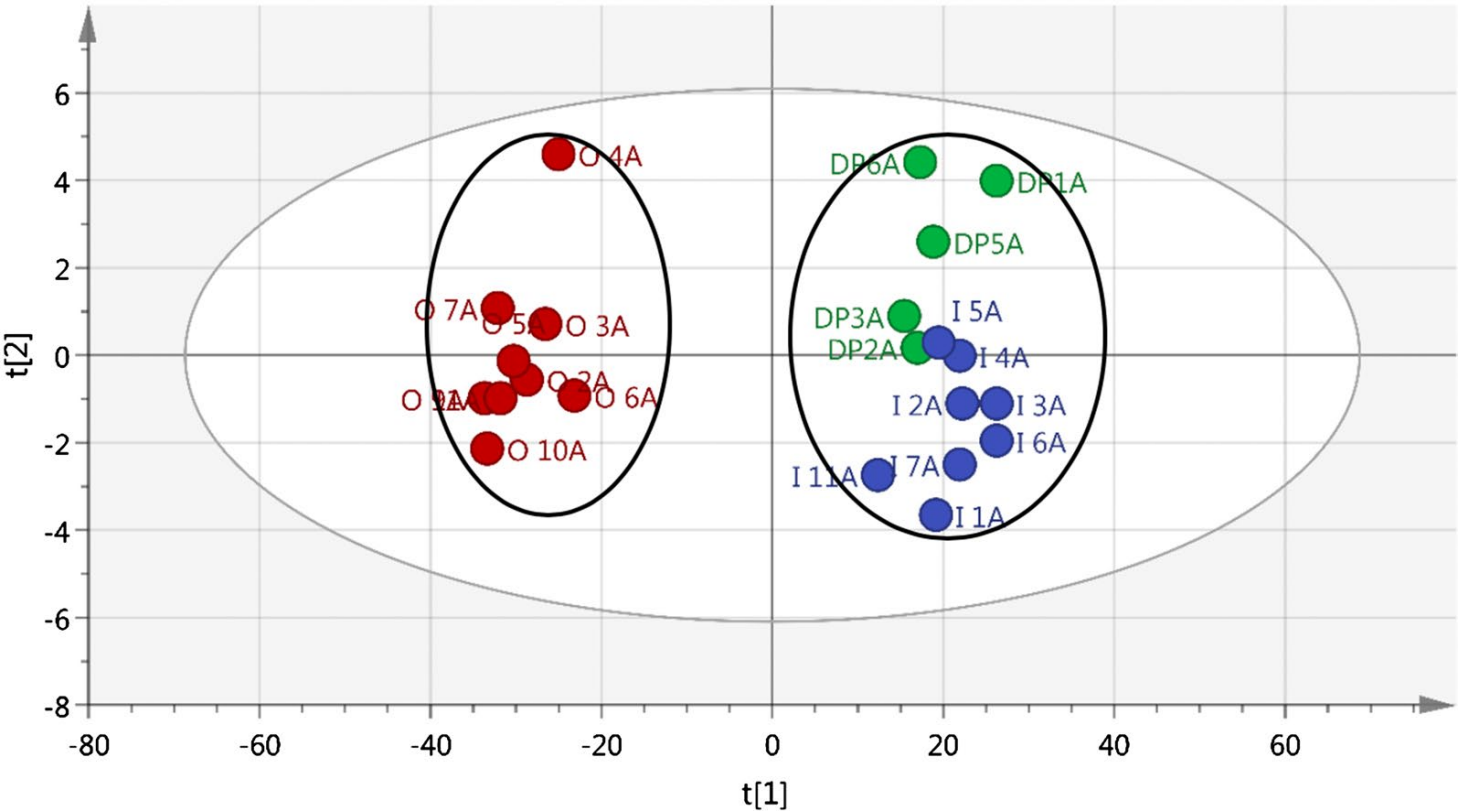
The PCA plot created from GC-MS spectra obtained from soil extracts for samples collected from DPs (green), FCs (blue) and matrix (red) collection sites. R^2^X(cum) = 0.998, Q^2^(cum) = 0.993

Principal Component Analysis (PCA) plots created from GC-MS spectra of soil extracts displayed a strong similarity between FC and DP soil, with higher concentrations of compounds being found in the DP soil (Fig. 4). There was a clear difference in chemical constituents between soil from the matrix and those from FCs and under dead *Euphorbia* plants. Many compounds typical to *Euphorbia* spp. were identified in the soil samples (see Additional file 1: Fig. S2).

#### Phytotoxic and antibacterial activity of *E. damarana* and *E. gummifera*

The immediate short-term effect of compounds from fresh stems and leaves of euphorbias co-occurring with FCs on grass seed germination under water stress, and also on soil bacteria isolated from the *Stipagrostis* grass rhizosphere, was investigated to provide more information on a possible mechanism for FC formation. We also report on the *E. gummifera* antibacterial and allelopathic compounds identified by GC-MS analysis. This species co-occurs with FCs in southern Namibia.

#### Germination inhibition assay

**Fig. 5:**
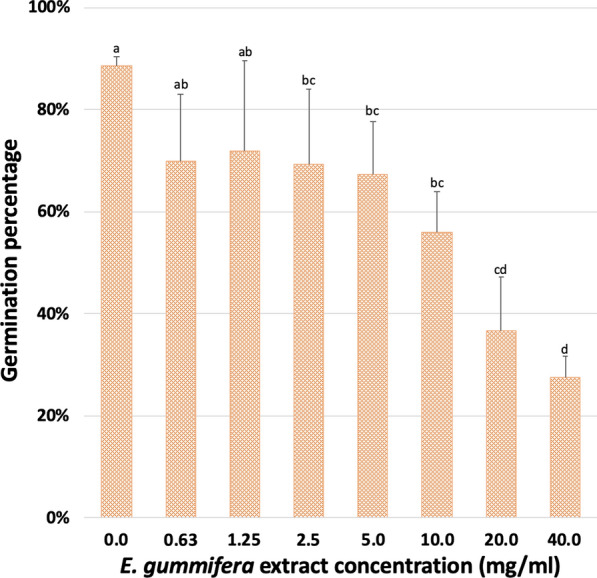
Germination inhibition of *Eragrostis tef* seeds by different concentrations of *E. gummifera* extracts when moistened with limited water (1 ml)

No significant germination inhibition was observed in the control and treatments when 2 ml of water was used to moisten filter papers in Petri dishes (p < 0.05). However, 2.5 mg/ml and higher concentrations of *E. gummifera* extracts significantly inhibited germination when only 1 ml water was used (p < 0.05) (Fig. 5). This indicated that there is a phytotoxic/allelopathic effect on seeds when they are experiencing water stress.

#### Antibacterial activity of *E. damarana* and *E. gummifera* extracts

Following DNA extraction from *Stipagrostis uniplumis* root rhizosphere bacteria (see Additional file 1: Fig. S3B) and sequencing, two bacteria were identified, *Pseudomonas paravulva* (97% similarity to the library sequence) and *Kocuria polaris* (99% similarity to the library sequence). The *E. gummifera* extract proved to be effective at inhibiting the growth of both *P. paravulva* and *K. polaris* at a concentration of 2.5 mg/ml. *E. damarana* extract inhibited the growth of several unidentified rhizosphere bacteria at a concentration of 0.31 mg/ml (see Additional file 1: Fig. S3*A*).

#### GC-MS analysis of *E. gummifera*

Several of the identified compounds from GC-MS analysis have previously been reported to be present in *Euphorbia* spp. and also for having significant antimicrobial activity, e.g. lupeol, quinic acid, α- and β-amyrin, betulin, lanosterol and lupeol acetate (see Additional file 1: Table S2). Two of the identified compounds with antimicrobial activity, have not previously been identified in the *Euphorbia* genus: lucenin 2 and heptacosanol (see Additional file 1: Table S2) [19].

### Spatial point pattern analysis

In order to determine if FCs and the *Euphorbia* species that co-occur with them have a similar spatial pattern, the spatial characteristics in three types of sites were analysed: (1) those with only FCs, (2) only euphorbias, and (3) where FCs and an *Euphorbia* species co-occur (mixed sites). These analyses were performed in three locations, Giribes and Palmwag in northern Namibia, Brandberg in the central-northern area and south in Garub.

#### Size comparison

The perimeter sizes of FCs were also determined and compared to the perimeter sizes of the co-occurring *Euphorbia* species of the location. Perimeter size comparisons were only done at Brandberg and Garub, since Giribes and Palmwag did not have enough plants or had no FCs respectively.

There were no statistical differences between the perimeter size of all the FCs and all the *E. damarana* plants at Brandberg (p = 0.019). No significant differences were also found between the perimeter size of FCs and *E. gummifera* plants at Garub (p = 1.527 × 10^−19^, Table 1; also see Additional file 1: Table S3).

**Table 1:** Mean perimeter of all FCs and *Euphorbia* plants in Brandberg and Garub

Site	Number of objects	Mean perimeter (m)
Brandberg FCs	2028	16.57
Brandberg *E. damarana*	2470	16.65
Garub FCs	1912	10.47
Garub *E. gummifera*	2171	9.47

#### Voronoi tessellations

**Fig. 6:**
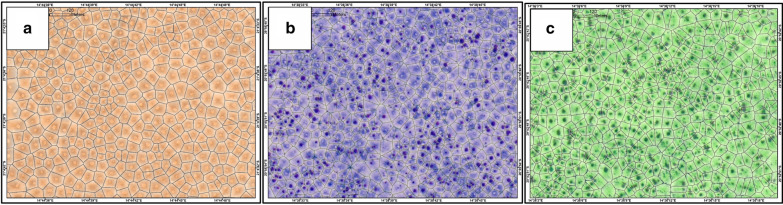
Voronoi tessellations created for FCs (**a**), mixed (**b**) and *E. damarana*-only (**c**) sites at Brandberg

The results of the Voronoi tessellations analysis showed that FCs, *E. damarana, E. gummifera* and the mixed sites had six neighbours on average, with a ‘hexagonal-like’ structure around each individual object (Fig. 6; also see Additional file 1: Table S4). This indicates a regular or uniform spatial distribution for all. The percentage of tiles with six corners differed in that the FC sites had the highest percentage of tiles with six corners (and were therefore the most overdispersed), followed by the mixed sites and then the plant-only sites. According to the pattern formation theory, overdispersed patterns are caused by repulsive interactions between events in the pattern [20]. This is often the case between plants that compete for resources, with the end result being overdispersion. With the hypothesis that FCs are caused by *E. damarana* (in northern Namibia) and *E. gummifera* (in southern Namibia), the FC-only sites are considered to be the end product of competition amongst them, and therefore having the most regular pattern. Subsequently, the mixed sites are considered to be in a transition state from *Euphorbia*-only to FC-only sites, and competition is still taking place between the plants. As the *Euphorbia*-only sites are in areas assumed to be ideal for them, e.g. rocky terrain with higher clay contents than in sandy areas, competition may be less between them. However these plant-only populations are also overdispersed because of some competition, although less so than in mixed and FC-only sites, in which competition is bigger because of the sandy substrate in which these sites are found.

#### Distance to nearest neighbour

A nearest neighbour (nn) R-value of zero is obtained when objects exhibit clustering and reaches 1.0 if the objects are randomly distributed. An R-value of more than 1.0 is indicative of a ‘regular pattern’, also referred to as ‘overdispersion’. Objects distributed in a perfect hexagonal pattern give a value of 2.149. The nn R-values of all sites were above 1.1 indicating overdispersion with the highest values recorded for FC-only sites (Table 2; see Additional file 1: Table S5), followed closely by mixed sites and then the plants-only sites.

**Table 2:** The average nearest neighbour R-values

Site	Site type	R-value
Giribes	Fairy circles	1.5
Giribes	Mixed	1.4
Palmwag	Mixed^a^	1.1
Palmwag	*E. damarana*	1.1
Brandberg	Fairy circles	1.4
Brandberg	Mixed	1.3
Brandberg	*E. damarana*	1.2
Garub	Fairy circles	1.4
Garub	Mixed	1.1
Garub	*E. gummifera*	1.1

^a^*E. damarana* mixed with sand circles (not FCs), see Additional file 1: Text S11, Fig. S14

**Fig. 7:**
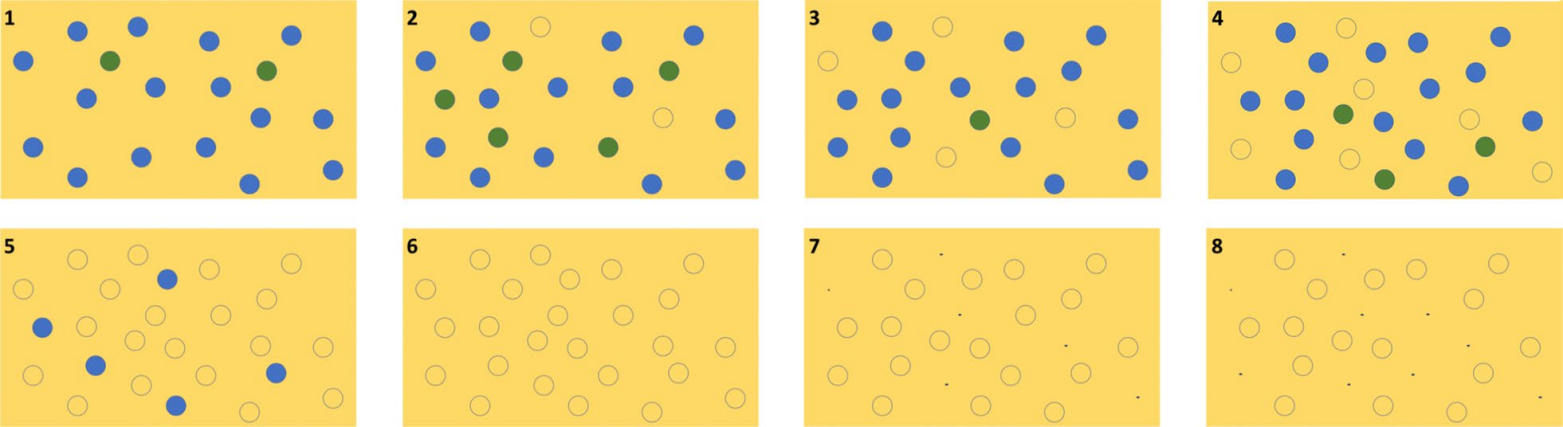
Proposed model of the formation of FCs (open circles) from *Euphorbia* species (blue circles). (1) Plants-only (green dots are juveniles), regular spatial patterning. (3) Plants and FCs mixed site, more regular spatial patterning because new plants established since no. 1. (6) Only FCs, most regular due to new plants and dead plants turning into FCs which form part of the analysis. (8) Only FCs, several years/decades later, clustered spatial patterning because FCs started to disappear. Cramer and Barger [4] found 42% of FC sites to be clustered

Competition is also evident in this analysis in the FC-only and mixed sites, these being always on extremely sandy soil with low water capacity and nutrient resources. At the plants-only sites, *E. damarana* is more overdispersed at Brandberg than at Palmwag. While Palmwag is considered to be representative of a typical location where *E. damarana* occurs on non-homogenous terrain and rocky soil, it will probably not cause FCs in the future. Brandberg is considered to be a site where *E.* *damarana* is transitioning into FCs on sandy soil. This indicated that conditions are less ideal for *E.* *damarana* at Brandberg (very sandy soil) and that competition for water and nutrients are causing these plants to be overdispersed. It is, therefore, not only competition between euphorbias, but also, and perhaps to a larger extent, the effects of the sandy desert environment that results in all plants dying out. Additionally, the plants are competing (R-values at all sites 1.1.‒1.2), and this also influences the pattern, resulting in the plants being spaced away from each other. A schematic model of how this process might evolve over time is shown in Fig. 7.

The lifespan of FCs (and euphorbias which unpublished radiocarbon dating analysis indicates have a lifespan of about 300 years) will influence the nn values and spatial patterning significantly. Locations like Giribes might be nearly at the end of the long transition process from only plants, to mixed sites to only-FC sites and some of the FCs might already have disappeared, resulting in bigger nn values. This might eventually lead to a more clustered distribution of FCs (Fig. 7) as Cramer and Barger [4] found and reported that 42% of the 80 FC sites analysed from South Africa to Angola were not overdispersed. All the FCs of Namibia therefore don’t have a unique spatial signature which is extremely ordered as is the case in Marienfluss, Giribes Plain and Namibrand Nature Reserve.

#### Pair correlation function

**Fig. 8:**
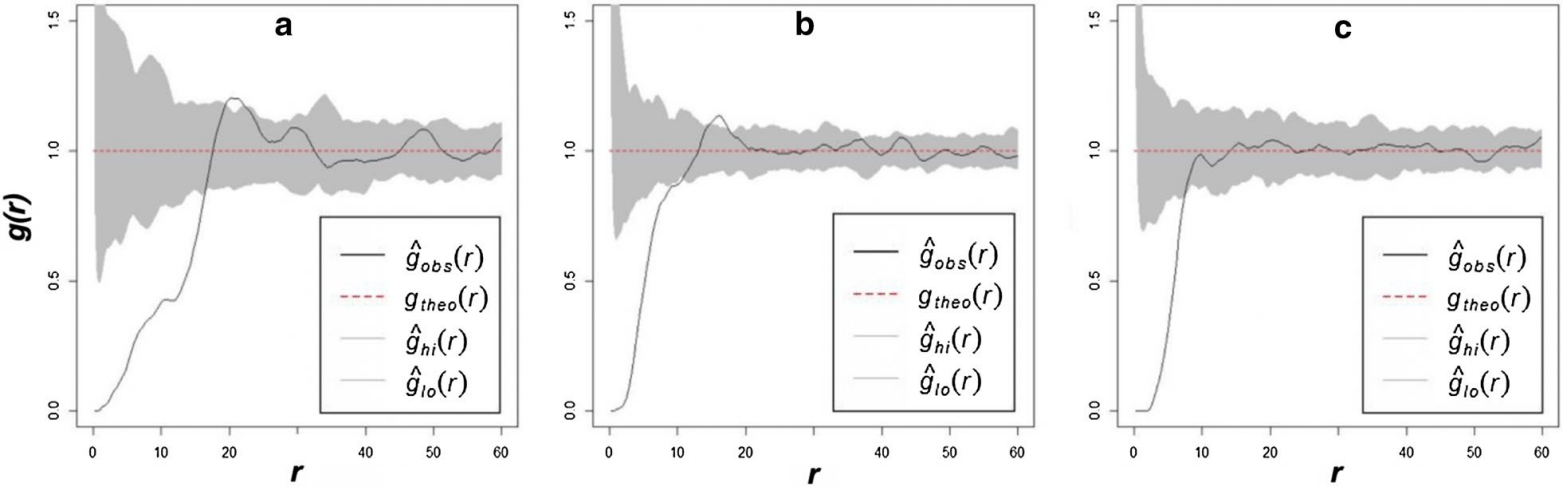
Graphs of the pair correlation function for the FCs (**a**), mixed (**b**) and *E. damarana* (**c**) sites at Brandberg

The pair correlation function results for the FC-only sites were very similar to the mixed sites but less similar to the *Euphorbia*-only sites (Fig. 8; also see Additional file 1: Fig. S4‒S6). The graphs of the pair correlation function start in a similar way for the FCs and *Euphorbia* sites as the g(r) value slowly starts to increase indicating a decrease in overdispersion, but still well outside the simulation envelopes. The rest of the *Euphorbia* graphs differ from those of the FCs. This indicates overdispersion for the first several metres but clustered patterns at larger scales [7].

If *Euphorbia* spp. are assumed to be causing FCs and the mixed sites are in a transition stage from a high percentage plants and a low percentage FCs, to fewer plants and mostly FCs, it can be argued that the mixed sites will become progressively more like the FC sites as more plants die (due to competition between the plants and/or environmental stress) as can be seen in Fig. 8. *E. damarana* and *E. gummifera* sites are generally less regular at larger scales than FC sites, which could indicate that these populations are not experiencing much competition and/or environmental stress. Interestingly, *E. damarana* at Brandberg does not share this clustering with the *Euphorbia* plants at Palmwag and Garub, possibly indicating that conditions are less favourable where these plants occur at Brandberg. It was observed that the plants-only sites at Brandberg are much sandier than those at Palmwag and Garub.

#### L-function

**Fig. 9:**
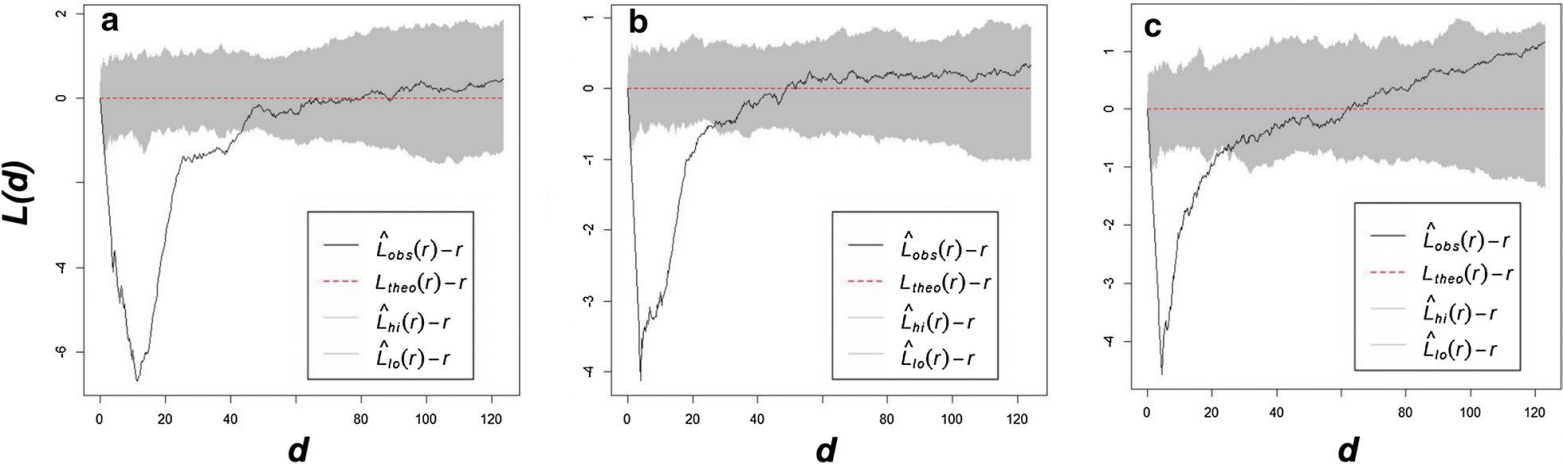
L-function graphs for FCs (**a**), mixed (**b**) and *E. damarana* (**c**) sites at Brandberg

The L-functions for the *Euphorbia*-only sites differed from the FC sites and were characterised by more inhomogeneity (Fig. 9; also see Additional file 1: Fig. S7‒S9). The *E. damarana* sites at Brandberg more closely resembled that of FCs than at the other locations and share the same large-scale homogeneity with the FC and mixed sites. The graphs of the L-function for nearly all mixed sites resembled that of the FCs, indicating regularity for the first few metres. In other words, the smaller scale properties of the pattern revealed by the pair correlation function are preserved mostly across a large area (i.e., hundreds of metres). These results show the same trends as those above and support the hypothesis that the mixed sites are transitioning zones between plants-only to FC-only sites.

### Historical image comparison

Since the FCs at Theron’s site marked in 1978 [13] were all still intact, and it was discovered that *E. damarana* (and possibly the other shrubby succulent euphorbias like *E. gummifera*) took at least more than 40 years to decompose, every avenue was investigated to find historical aerial photographs from pre-1970. The only useable one that could be found was from 1966 on which the *E. gummifera* plants in Garub could be identified. The results of the comparison of this photograph with recent satellite images and ground-truthing are reported here.

#### Giribes Plain

**Fig. 10:**
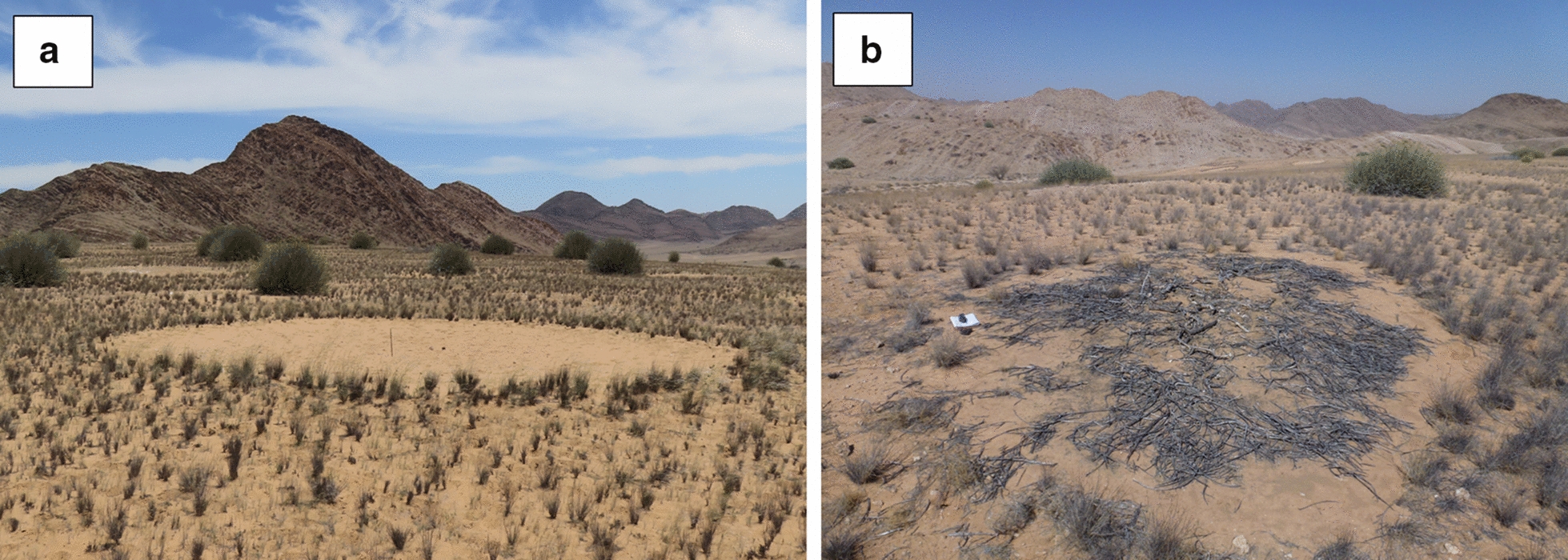
Fairy circle marked by Theron [13] (**a**) and remains of *E. damarana* (**b**) on Giribes Plain

Ten FCs and 3 dead *E. damarana* plants that were marked by Theron in 1978 [13], were located, ground-truthed, photographed and their GPS coordinates recorded, so that they could be located on a satellite image. Although the grass cover was at a minimum, all the FCs and the dead plants marked in 1978 were still intact in 2016 (Fig. 10). A few small grasses were present inside some of the FCs. These FCs were also confirmed as intact in 2014 [1] and therefore it can be assumed that the minimum age of FCs is at least 40 years. These observations support the notion that FCs live long and do not permanently disappear after dry spells as the vegetation self-arrangement hypothesis states [3, 9, 15]. It also shows that the decay process of *E. damarana* takes an extremely long time.

#### Garub

**Fig. 11:**
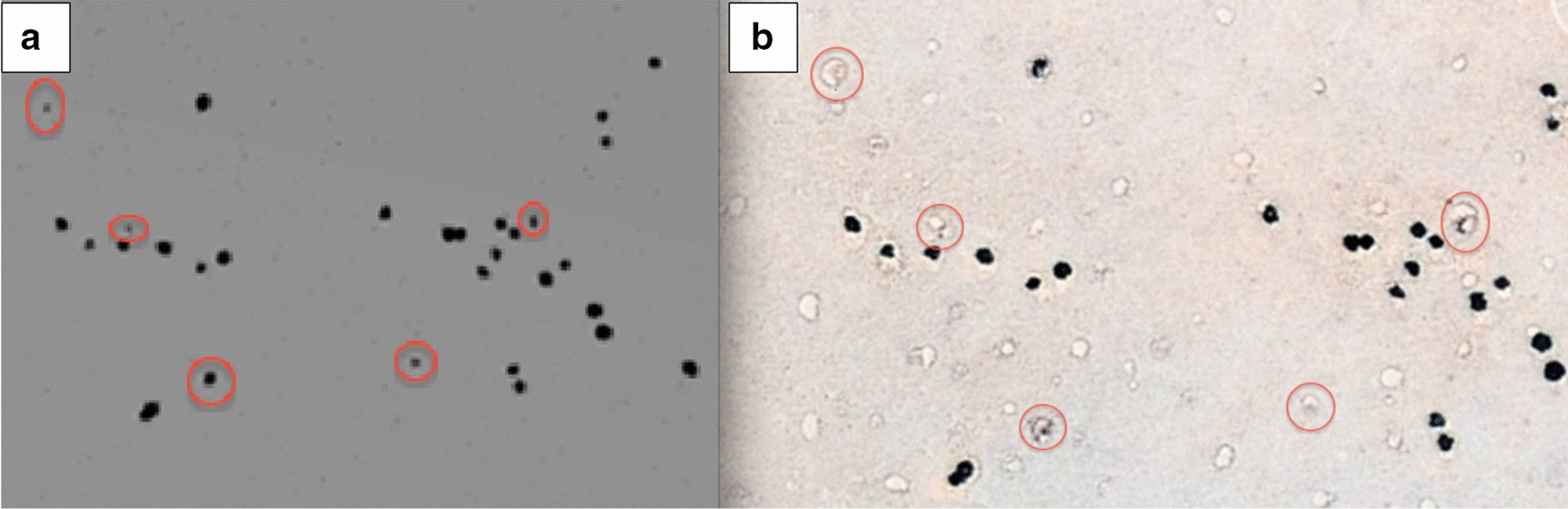
An aerial image (source Ministry of Lands, Namibia) of *E. gummifera* in 1966 at Garub in southwestern Namibia (**a**) and the corresponding area in 2011 on Google Earth™ (**b**). Red circles indicate *E. gummifera* plants (**a**) and developing FCs (**b**)

Figure 11 shows part of an area in Garub in 1966 and the same region in 2011. The black dots were ground-truthed in 2015 as *E. gummifera*, and many were observed to have been replaced by FCs. The remains of some of the plants were also observed inside the barren patches. All the objects that were identified from the aerial and satellite imagery were confirmed to be FCs or *E. gummifera* plants.

The image comparison revealed that of the test sample of 406 *E. gummifera* plants that were alive in 1966, 69 were dead (with remains still present) and 134 of them were replaced by FCs in 2011 (Table 3). This image comparison clearly illustrates that dead *E. gummifera* plants are replaced by FCs and indicates that the plants are in the process of transitioning into FCs at Garub in southwestern Namibia. Other authors recently agreed that *E. gummifera* cause the FCs in this area [16].

**Table 3:** Long-term observations of *E. gummifera* and FCs at Garub

Object	1966	2011
Living *E. gummifera*	406	203
Dead *E. gummifera*	Not possible to determine	69
Fairy circle	Not possible to observe	134

### Site suitability analysis for prediction of FC distribution

As the previously reported FCs all occur in a narrow band near the Atlantic coastline (low altitudes) on sandy soil and at a low mean annual precipitation (MAP) of about 100 mm, it was necessary to know if these conditions are not found elsewhere in Namibia, and with the possibility of discovering newly documented FCs. A digitised FC prediction map was compiled using the above-mentioned parameters and validated with randomly created points inside and outside the prediction area. Since succulent euphorbias are hypothesised as the cause of FCs, their distributions were overlaid on the prediction map. The results are reported in this section.

#### Site suitability prediction model

The selection criteria of suitable rainfall (50–150 mm MAP) and altitude (500–1200 m above mean sea level (mamsl)) maps for FC formation in Namibia are shown in Additional file 1: Fig. S10. The suitable areas lie mainly in a narrow band from the north to the south of Namibia and cover an area of 220,734 and 252,897 km^2^ respectively. Towards the south the areas widen and expand more inland up to a distance of 400 km from the coast. This indicates that the same precipitation range and altitude where FCs are found near the coastline, extends to the southeast of Namibia up to the South African Border and into the Kalahari Desert (data was only analysed in Namibia). The intersection between the suitable rainfall range and suitable altitudinal range (see Additional file 1: Fig. S10) is considerably smaller and covers an area of 148,203 km^2^.

The sandy grass plains were extracted into a single layer (104,443 km^2^) (see Additional file 1: Fig. S10) and also ‘crossed’ the South African Border into the Kalahari Desert.

**Fig. 12:**
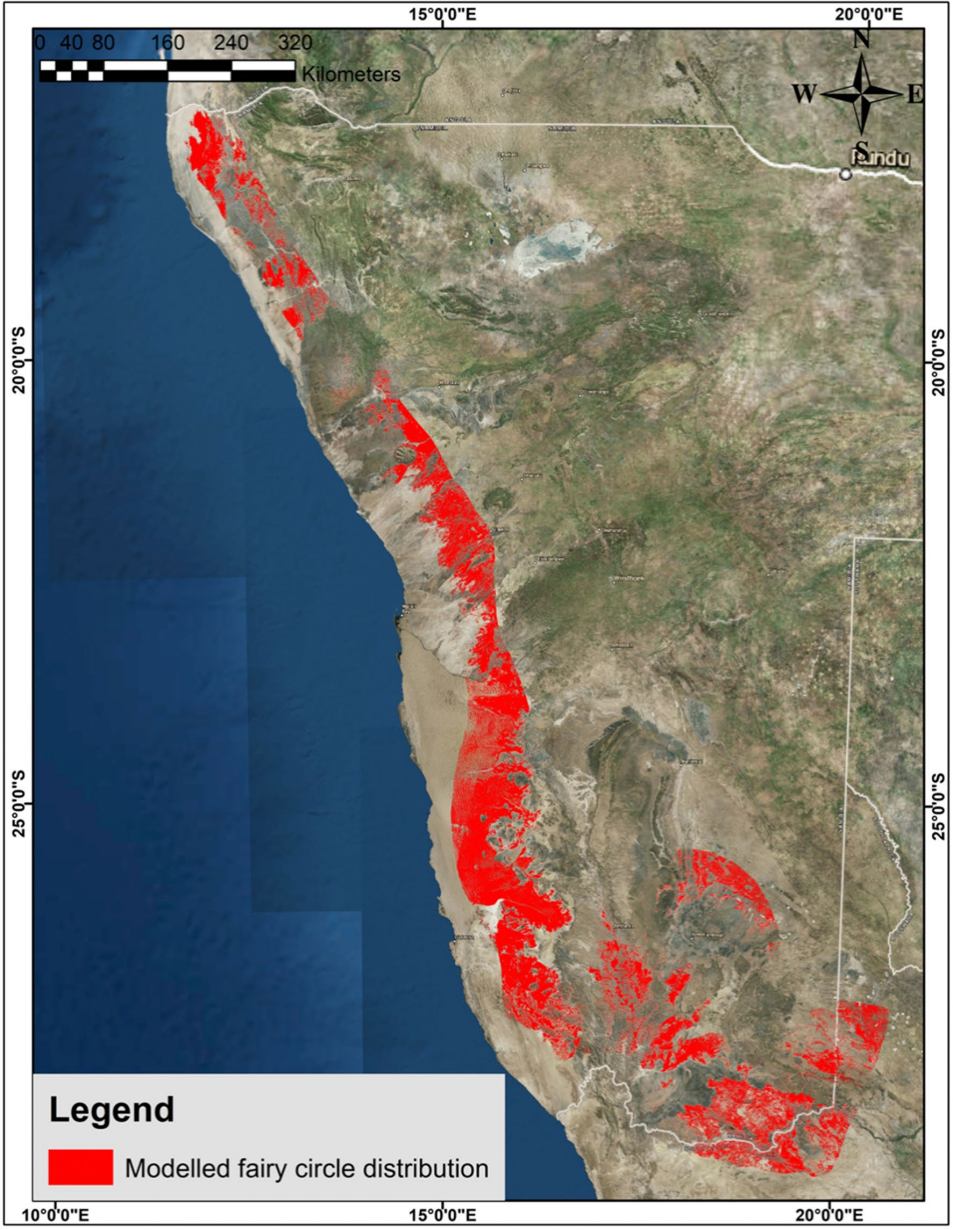
Site suitability prediction map for fairy circle distribution (areas where previously reported FCs occur south-west of the Orange River are not included here). Created in ArcMap

Finally, a combined layer was formed by intersecting the suitable rainfall and altitudinal ranges with the grasslands on sandy soil. This output represents the areas where all three predictor variables are present and thus the areas where FCs are likely to occur (Fig. 12). The modelled FC distribution covers an area of 55,955 km^2^ and indicates that there is a new unreported area in the southeast of Namibia, extending into a part of the Kalahari Desert, where FCs should occur, that has not been identified previously. The occurrence of FCs in these areas was confirmed by analysing Google Earth™ images.

**Fig. 13:**
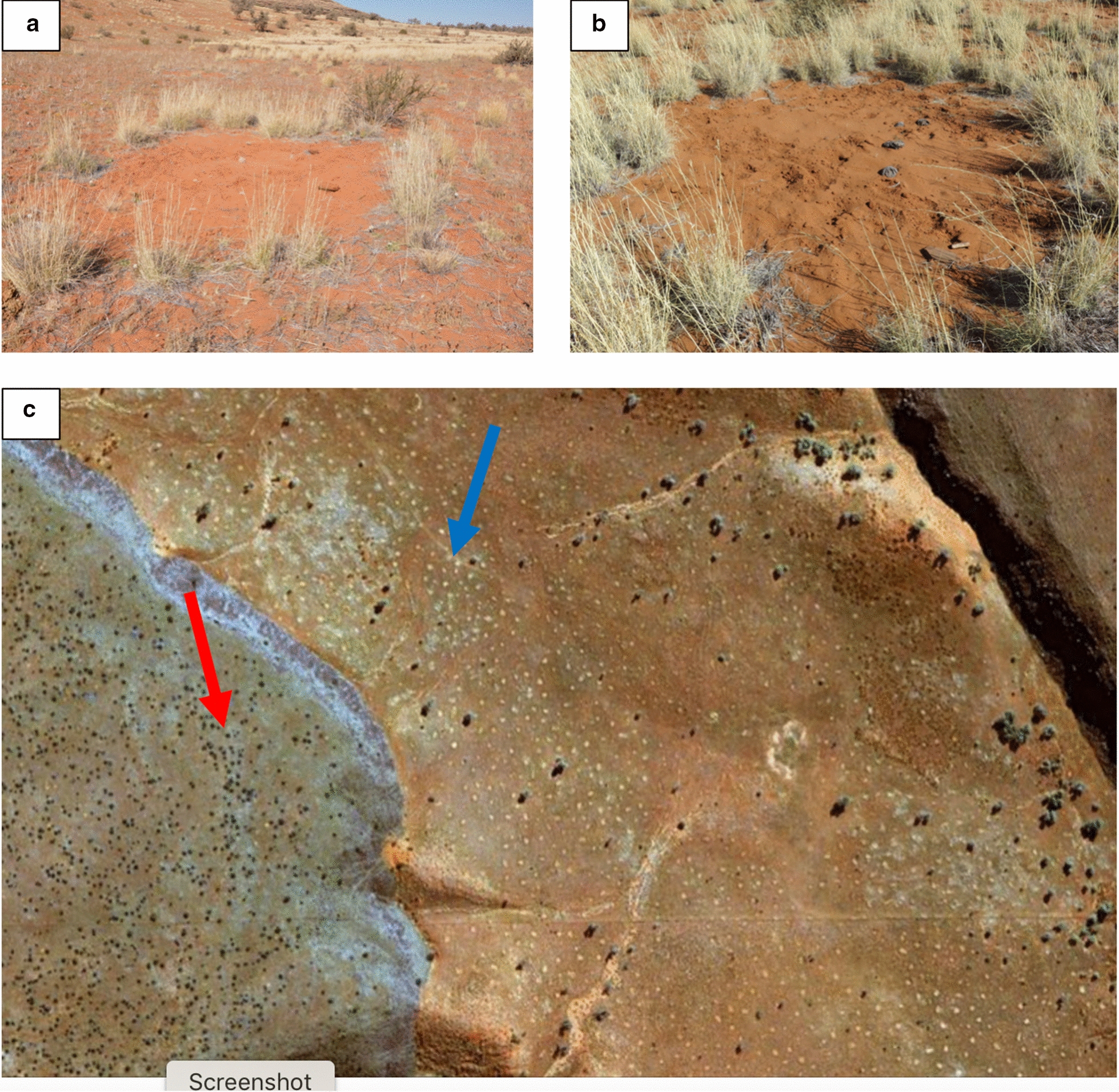
Examples of FCs (**a**, **b**) and many more (**c**, blue arrow, source Google Earth™) and *E. gregaria* (on the high river bank, red arrow in **c**) in the Bakrivier, Kalahari Desert, South Africa, predicted to occur in this area by the modelled prediction map (27°58′54.466″S; 20°02′10.913″E)

A significant new finding was that FCs were discovered inland as far as 400 km from the coastline in the southeast of Namibia and even in a part of the South African Kalahari Desert (Bakrivier) (Fig. 13; also see Additional file 1: Fig. S11). These areas were ground-truthed in 2018.

#### Prediction model validation

**Fig. 14:**
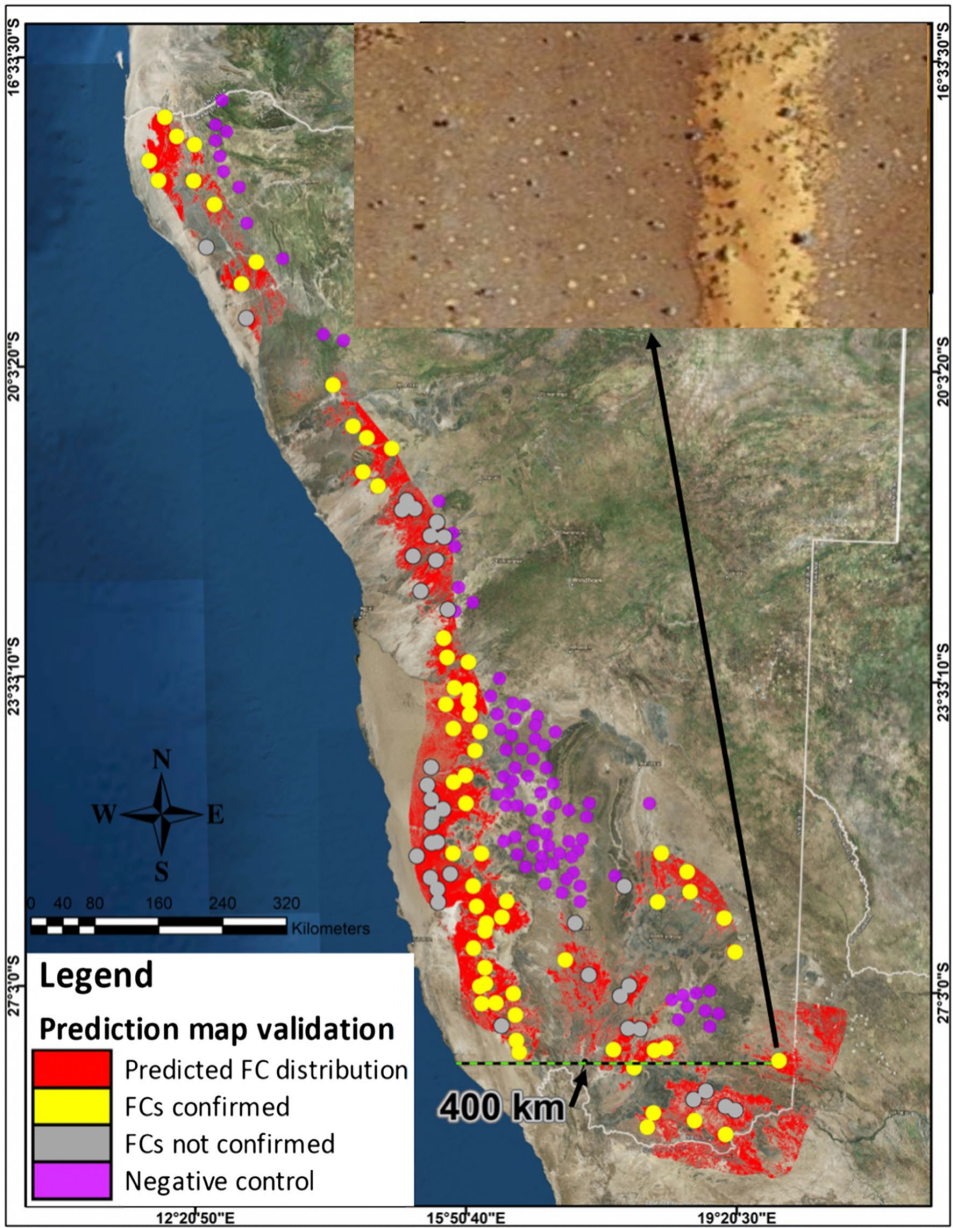
Fairy circle prediction map (red) validation with random points. Yellow indicates areas where FCs have been observed, while FCs were not observed in grey areas. Purple dots indicate the negative control, where no FCs were predicted and also not found. New location of FCs (enlarged area, 27°52′55.85″S; 19°56′3.96″E). Created in ArcMap

The validation process involved generating 100 random points in areas predicted to have FCs (positive control) and also 83 random points (relative area smaller) where FCs were not predicted to occur (negative control) (Fig. 14). Google Earth™ and ground-truthing in a few locations were used to determine whether FCs were present.

Fairy circles were found in the positive control areas in 65 out of the 100 points generated for the modelled FC distribution. It is believed that the 65% hit rate was considerably lower than in reality as a result of a lack of grass cover during dry seasons on several of the available Google Earth™ images (e.g. inside the Namib Sand Sea). This lowered the validation accuracy percentage of the model. No FCs were found in the 83 random points predicted not to contain FCs.

#### Comparison of prediction map with distribution of *Euphorbia* species

Two factors should be taken into consideration regarding the *Euphorbia* distribution data sets. Firstly, plant species are usually under-collected in remote areas and are mostly restricted to areas where there are roads [21], whereas the suitability model is unrelated to human access and covers areas not easily reached. The second factor to consider is that the *Euphorbia* distribution includes all areas and is not restricted to the prediction map criteria of rainfall, altitude and sandy grassland cover.

**Fig. 15:**
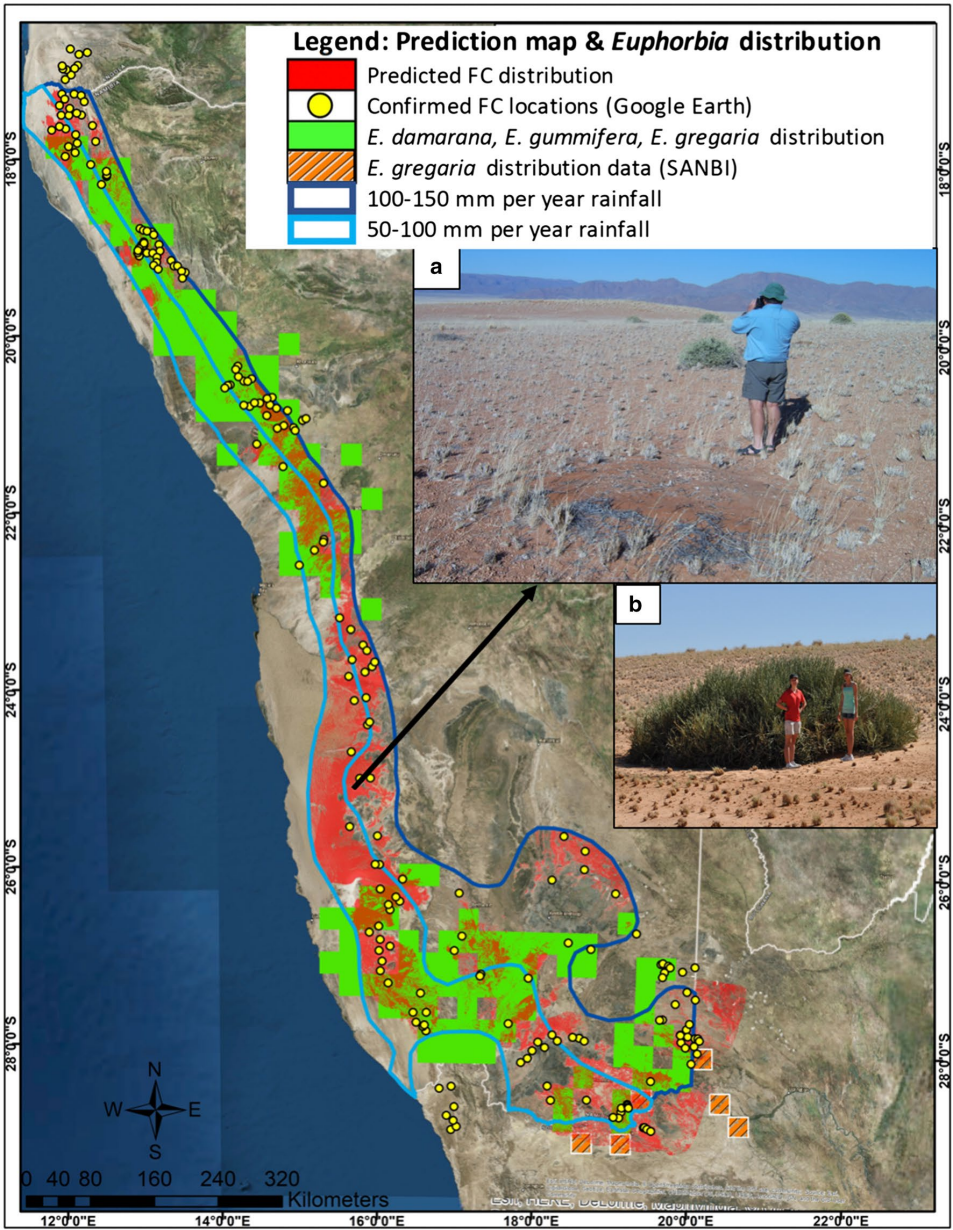
The site suitability prediction map is shown with the distribution of *E. damarana*, *E. gummifera* and *E. gregaria* (green layer). The yellow points indicate confirmed FC locations identified from Google Earth™ images. Small FCs co-occurring with *E. lignosa* in Namibrand (**a**). An *E. gummifera* specimen in Garub (**b**) which has also been reported on inselbergs in Namibrand [22]. Created in ArcMap

Figure 15 shows the good correlation of the distribution of *E. damarana, E. gummifera* and *E. gregaria* to the FC prediction model. The area where there is no overlap between the site suitability map and the distribution of euphorbias, lies in close proximity to the Namib Sand Sea, where FCs cover vast areas, extending east into the valleys of the escarpment, and west into the sand sea itself. In this area the presence of *E. lignosa* Marloth in the Namibrand Nature Reserve, where they co-occur in-between small FCs (Fig. 15a), was noted. The presence of *E. lignosa*, as well as *E.* *gummifera* was reported on inselbergs inside Namibrand Nature Reserve, as well as in the Namib-Naukluft Park [22].

The distribution of *E. damarana* in Namibia is confined to the northwestern parts (see Additional file 1: Fig. S12) and associated to a big extent with the FC distribution from near the Angolan Border to Brandberg to approximately 100 km north of the Namib Sand Sea. Several areas were observed where FCs co-occur with *E. damarana* that have not been documented before, e.g. at Brandberg. This suggests that the co-occurrence is more general than previously thought [1, 16, 23, 24].

*E. gummifera* is confined to the southwestern parts of Namibia (see Additional file 1: Fig. S12) and covers the smallest area of the three species of *Euphorbia*. The distribution of *E. gummifera* is associated with the FC distribution from slightly south of the Namib Sand Sea to the Orange River (this study only focused on Namibia).

*E. gregaria* is widespread throughout the southern parts of Namibia (Additional file 1: Fig. S13) and associated with the FC distribution in these parts of Namibia. New FC locations (e.g. south of Grünau and the Kalahari Desert in South Africa), as well as co-occurrences with *E. gregaria* were ground-truthed in 2018 at locations indicated in Fig. 13, Additional file 1: Figs. S11 and S13. Several areas of co-occurrence were observed on sandy soil where big proportions of *E. gregaria* are dying.

## Discussion

### Physical and chemical properties of soil

The results of the soil analysis showed that the soils associated with FCs and the sites where *E. damarana* (in northern Namibia) and *E. gummifera* (in southern Namibia) co-occur in mixed sites are very sandy and consist of the same range of particle sizes.

It is proposed that the differences in the chemistry between soil from FCs and the matrix, can be ascribed to *Euphorbia* spp. that have grown where FCs are now present. GC-MS analysis and metabolomical PCA multivariate statistical analysis clearly showed the similarity of compounds from FCs and from soil collected under decaying *E. damarana* plants. The long-lasting effect of the compounds from the *Euphorbia* species is the hydrophobicity of the latex and not the intact toxic compounds. Compounds originating from the euphorbias had probably undergone two stages of chemical reactions, the first reaction being a decomposition reaction which occurred when the plant was decomposing. The second being the reaction between plant latex/resin with the soil organic and inorganic matter, when the resin molecules formed a chemical bond with the matter present in the soil. It is thus almost impossible to identify the exact same molecule in the soil and in the plant, given the extreme lapse in time from the death of the plant, to the decomposition of the plant material and ultimately to the bonding of decomposed chemicals to the soil inorganic and organic matter.

The differences in the soil’s chemical properties, such as the hydrophobicity of most soil samples collected from FCs, are most likely caused by the decomposition of *Euphorbia* spp. The decomposition subsequently leads to the alteration of properties such as the water runoff and increased preferential flow [17, 18]. It has previously been reported that hydrophobic substances like resins/latex, especially in sandy soil, can cause allelopathy, suppress germination and improve water conservation by channelling water deep into the soil profile, following preferential flow pathways (previous stem and root activity), while at the same time reducing evaporation due to the partial dryness of the surface soil layer [17]. These hydrophobic properties could be simulated in the current study by coating matrix soil with *E. damarana* latex. A previous study [3] reported faster infiltration rates and thus lower infiltration times per volume of water in larger FCs. We would ascribe this to larger euphorbias containing more latex, and therefore causing more hydrophobicity and faster infiltration rates (as measured with a single ring infiltration meter). It would be valuable to determine whether the adhesive resin produced by spinifex *Triodia* grasses in western Australia [25] is playing a role in the formation of the barren circles, resembling fairy circles, observed there [8]. The water runoff and low k-values (as measured with a disk infiltrometer in which hydrophobicity plays a significant role) indicates the hydrophobicity of the soil, possibly being caused by previous growth of resin-containing *Triodia* grasses [8]. This hydrophobicity could be related to the mechanism of the *Euphorbia* theory which proposes that the sticky, coagulant, hydrophobic terpenoid-containing polymers can change the soil properties? The resin of the soft spinifex species like *T. pungens* found in the Newman area were previously used to make handles of spears and knives. It was ground to a fine powder, heated with fire causing the resin to melt and coagulate, rolled into a ball or cake and used as an adhesive, for attaching stone blades to wooden handles such as spears, knives and chisels [25]. Getzin et al. [8] stated that the gaps, stripes, spots, bare soil, and transient patterns such as rings were induced by frequent fire disturbances.

Furthermore, the hydrophobicity of soil in FCs could be the cause of the peripheral belt of grasses associated with FCs, since rain water will flow over the surface of the hydrophobic FC soil and will infiltrate at the periphery of the FCs [17]. The result being an increased amount of soil water which is available to plants on the periphery, and a decrease in the soil water content available for seedling establishment within the upper layers of FCs [2–4, 6, 7]. This would result in the observations that seedlings are short lived within FCs. Although the increased hydrophobicity and faster infiltration rate is seemingly contradicting, it is important to note that hydrophobicity decreases the water retention ability and capacity, resulting in water infiltrating with more ease due to the lack of adhesive forces between water and the hydrophobic sand particles, resulting in increased cohesion within the water [17]. Furthermore, it must be understood that the thermodynamic interaction between a water droplet and soil particles in the Water Droplet Penetration Time (WDPT) test is different than that during the infiltration time determination. During the latter, the thermodynamic barrier between the water and the soil surface is overcome once substances originating from the soil, migrated to the water, ultimately decreasing the water surface tension, allowing infiltration to occur. This mechanism supports findings that hydrophobic sandy loam soil has been reported to contain as much as 22% moisture [17]. During the WDPT-test, the effect of the soil profile potential and gravitational force is infinitesimally small due to the small volume of water. The WDPT-test is exclusively used to determine soil hydrophobicity, and not infiltration time. The results of the WDPT-test can however be used to interpret and understand the behaviour of liquids in soil, due to the predetermined severity of hydrophobicity.

### Phytotoxic and antibacterial activity of *E. damarana* and *E. gummifera*

Inhibition of germination in *Eragrostis tef* grass seeds by *Euphorbia gummifera* extracts was only significant under water stress. The importance of resource limitation, especially water stress, in allelopathy and germination inhibition in particular, has previously been emphasised [26, 27]. Concentrations of 2.5 mg/ml and higher of *E. gummifera* extract inhibited germination significantly compared to the control. It is believed that much higher quantities of plant metabolites enter the soil after the plant’s death. Several latex-producing species belonging to the *Euphorbia* genus have been documented to have allelopathic properties manifested in germination inhibition [19, 28–30]. Several authors previously conducted seed germination experiments on soil from inside, outside, the edge of FCs and even from soil underneath dead *Euphorbia* plants and found no germination inhibition [5, 13, 31, 32]. It is believed that these experiments would have shown inhibition if they were conducted with very limited water to simulate pro-desert conditions. Future water-stressed germination and growth inhibition experiments in pot-based bioassays of soil from FCs, the matrix and from underneath dead euphorbias could present a more realistic picture of field conditions.

A microtiter-based antibacterial assay showed that *Stipagrostis uniplumis* grass rhizosphere bacteria were inhibited by both *E. damarana* and *E. gummifera.* In the current study only a limited number of rhizosphere bacteria could be cultured and identified but many other latex-containing *Euphorbia* spp. have been found to exert antibacterial activity on several bacterial species [19, 33–35]. Metagenomical studies on the rhizosphere bacteria and fungi of *Stipagrostis* species and their presence/absence in soil from FCs, the matrix and from underneath dead euphorbias, could prove to be interesting.

A number of the compounds identified by GC-MS analysis are hydrophobic and have previously been reported for antimicrobial activity (see [19] for a comprehensive review of these). Lupeol, for example, had been referred to as a strong antimicrobial compound with extremely low minimum inhibition concentrations [36]. The presence of these compounds in *E. gummifera* and previously found in *E. damarana* [37] most likely explains their observed antibacterial activity. It has previously been shown that *Stipagrostis* grass species survive in the harsh pro-desert conditions with the assistance of a microbial rhizosphere [38, 39]. Large quantities of antimicrobial compounds entering the soil will most likely have a detrimental effect on these microbes.

### Spatial point pattern analysis

There is no statistical difference between the perimeter sizes of FCs and the *Euphorbia* spp. in the same areas. This suggests that instead of only rainfall determining FC size [3, 10], it may rather be determined by the size and species of the *Euphorbia* that gave rise to the FC. This could explain why FCs are generally larger in northwestern Namibia where *E. damarana* occurs, than in the southwest where *E. gummifera* is found.

The spatial point pattern analysis revealed that FC sites and co-occurring FC-*Euphorbia* mixed sites are overdispersed. *Euphorbia*-only sites are also overdispersed, but to a lesser extent. Overdispersed or uniform patterns are associated with repulsive interactions between events in the pattern, and in an ecological context often point toward competition for resources. These being more profound in sandy habitats than in areas with a higher clay content, both current habitats for *Euphorbia* spp. According to the vegetation self-arrangement theory the individual grass tufts are competing between themselves to form FCs [10]. The regularity associated with the spatial pattern of FCs and the correlation of FC size with their distances apart, are also consistent with the notion of plant–plant interactions as the major vegetation-patterning mechanism [11]. The current study showed that the nn R-values of FCs and co-occurring *Euphorbia*-FCs sites are similar and to a lesser extent similar to the nn ratio’s of *Euphorbia*-only sites. The mixed sites are hypothesised to be in an intermediate state, progressing from a high density of plants and low density of FCs, towards a lower density of plants and higher density of FCs. This is supported by the spatial analysis results which showed that the mixed sites share physical characteristics with both FCs and euphorbias. The pair-correlation function for FCs and the mixed sites are similar in that both sites display a significant peak; however the distance at which the peak is formed is greater for FCs than mixed sites.

The satellite images used to analyse the point patterns provide only a snapshot in time of the process that is hypothesised to be creating FCs. It is unknown what the length of time required is for an individual *Euphorbia* to reach maturity, for how long the plant will live and how long it will take to decay. The results of this study have shown that the decay process of *E. damarana* is more than 40 years and unpublished radiocarbon dating indicated the lifespan of the plant to be about 300 years. Once the plant has decayed completely, a FC is hypothesised to become visible. It is also uncertain how long a FC will persist. Future work to construct a model using the population dynamics of *E. damarana*, *E. gummifera* and *E. gregaria,* instead of variables representative of termite population dynamics [11], could be valuable.

FCs have previously been hypothesised to disappear during periods of prolonged droughts (when grass cover is absent) and reappear, not necessarily at the same place, after sufficient rainfall (when grass cover is present) [10]. According to the *Euphorbia* theory, the FCs will however appear in the same places after sufficient rainfall. A number of published historical photographs with FCs that are formed in locations where there were none previously, and also no euphorbias, are unclear and not conclusive [2, 7, 15]. The visualisation of FCs are highly dependant on previous rainfall and therefore the absence/presence of grass cover. More evidence is required to prove such appearances of new FCs.

### Historical image comparison

The objective of this part of the study was to conduct long-term observations of FCs, *E. damarana* and *E. gummifera*, in order to determine the approximate age of FCs and to observe what happens to the *Euphorbia* populations over time. In the analysis the exact same FCs marked with steel pins in 1978 at Giribes were located and found still to be intact [1, 14], which supports the notion that FCs can persist for decades through dry/wet spells. The aerial image comparison at Garub revealed the formation of more than 100 FCs resulting from the death and decay of *E. gummifera* plants.

The historical image comparison also showed why FCs nearly always display a circular shape. It was suggested that grasses form a ring-like structure because a circle has the smallest circumference-to-area ratio [10]. This study has shown that FCs exhibit a mostly circular shape because the plants that give rise to them are mostly circular. Many ‘mega-FCs’ [16] or sometimes kidney-shaped FCs have been observed, and likewise euphorbias have also been seen growing next to each other (Additional file 1: Figs. S12A, B and S16C) which will eventually result in the formation of such non-circular FCs. It is also hypothesised that ‘mega-FCs’ [16] will form along drainage lines (e.g. dry river beds) when occasional high rainfall occurs, moving the latex-covered sand downhill and forming long-elliptical FCs.

### Site suitability analysis for prediction of FC distribution

The validation of the prediction model based on altitude, rainfall and type of land cover provided encouraging results, which indicated that the three selection criteria used provided a relatively accurate measure of the presence of FCs in Namibia. Likewise, the negative control also provided validation for the use of the three variables. The accuracy would probably have been higher if more satellite images were available of periods in which land cover was better. Thirteen of the random points in the positive control were, for example, inside the Namib Sand Sea where grass cover is virtually always absent.

One major finding from the suitability model was that FCs were predicted to occur in the far southeast of Namibia, including the Nama-Karoo Biome and the Kalahari Desert. This was confirmed by ground observations and is a significant new finding in the FC phenomenon.

In order to determine whether there is an association between FCs and *Euphorbia* spp., the site suitability map was compared to the distribution of *E. damarana*, *E. gummifera* and *E. gregaria*. The distribution of *E. damarana* is closely associated with the predicted FC distribution in the northwestern and central western parts of Namibia, while the distribution of both *E. gummifera* and *E. gregaria* are closely associated with FCs near the southwest coast to the southeast of Namibia. This is the first documented co-occurrence of FCs with *E. gregaria*. Large areas have been identified where high numbers of especially *E. gummifera* and *E. gregaria* are dying in sandy soil and several FCs were observed between these DPs. These areas are considered to be in the beginning stages of transitioning into areas with low numbers of these plants and large numbers of FCs.

The distribution of the succulent euphorbias covers the distribution of the FCs in the prediction map much more closely than the termite *Psammotermes allocerus*, which extends beyond the rainfall isohyets associated with FCs. No termite activity related to FC formation was observed in this study over several years.

This study has shown that *E. damarana*, *E. gummifera* and *E. gregaria* are limited to an almost identical rainfall range as FCs, but to a lesser degree restricted to the altitude range of FCs. The reason being that euphorbias will only cause FCs when they are growing on sandy soil, which has faster infiltration rates and a low water-holding capacity and therefore are sensitive to droughts and rising temperatures, as has been reported to occur in Namibia during the last 25 years [40, 41].

## Conclusion

The FC phenomenon has resulted in decades of detailed, scientific investigations with contrasting hypotheses regarding their origin and maintenance. The *Euphorbia* spp. allelopathy theory has received little attention during this time, apart from two reports that both focused on the FCs of Garub in southern Namibia [12, 16]. There have been three main points of critique against the *Euphorbia* theory. Firstly, *E.* *damarana* apparently prefers to grow on a stony coarse-textured substrate [1, 23]. Secondly, there has been a lack of reports on the co-occurrence of FCs and *Euphorbia* spp. [15, 23], and thirdly, *E. damarana* is spatially irregularly distributed and could therefore not result in the observed regular pattern of FCs [24].

This study has firstly documented vast areas where *E. damarana*, *E. gummifera* and *E. gregaria* occur on sandy plains near Giribes, Brandberg, Garub, southwest of Keetmanshoop and several other areas in Namibia, and are therefore not restricted to stony coarse-textured areas. Secondly, this study and previous ones [12–14] documented the co-occurrence of these species with FCs in several areas. The occurrence of *E. gummifera* has also been reported on inselbergs in the Namibrand Nature Reserve [22], where they still survive and are surrounded by tens of thousands of FCs. This species indicates an altitudinal upward movement by growing on plains and lowlands in the Succulent Karoo, but only occurs on inselbergs at study sites north of the Succulent Karoo [22]. Thirdly, we showed that *E. damarana* and *E. gummifera* have distance to nn ratio R-values bigger than 1, and are therefore not clustered in spatial arrangement.

Before a hypothesis on the cause and maintenance of FCs can be accepted it would have to account for all the important properties of FCs—their mostly circular, or occasional ‘mega-FC’ or long-elliptical shape on drainage lines [16], their overdispersion, their size and their changing diameter at different latitudes. We propose that the *Euphorbia* theory can probably explain all these characteristics, although we presented data on only three areas in southern, central and northern Namibia. The circularity of FCs can be explained by the fact that *E. damarana*, *E.* *gummifera* and *E. gregaria* have mostly circular shapes and occasionally grow next to each other, forming kidney-shaped FCs as mentioned above (Additional file 1: Figs. S14‒S17). We also presented evidence that FCs are the product of dead *Euphorbia* plants in the studied areas as indicated in the soil analysis, the spatial analyses of the transitional mixed sites, and the historical imagery comparison in Garub and Giribes. The soils are extremely sandy in these areas and could possibly result in a hydraulically connected landscape [3], enabling the mobilization of edaphic resources for which the euphorbias compete. Together with the environmental stress of such arid areas, this process eventually results in the observed regular/uniform pattern of FCs. We also found no statistical difference in size between FCs, *E. damarana* and *E. gummifera* in areas where they co-occur.

We also found significant differences between FC and matrix soil in terms of physical and chemical properties, and that these differences can be ascribed to the decomposition of *Euphorbia* spp. This study also illustrated that *E. damarana* and *E. gummifera* possess biological activity, as demonstrated by its phytotoxic/allelopathic and antibacterial activity. The results also provided valuable information regarding the chemistry of *E. gummifera* which could be linked to its biological activity through the identified compounds.

Finally, if the findings of this study are correct and applicable to all FCs, then an astonishing record exists of the ‘footprints’ of hundreds of thousands of succulent euphorbias that have died in several areas of the Namib pro-desert, stretching from Angola in the north and South Africa in the south.

We hypothesise that *Euphorbia* spp. colonised sandy plains when climatic conditions were more favourable in the past. Since sandy soils have low water-holding capacity, and might facilitate the formation of a hydraulically connected landscape, these plants would have been under pressure for water and nutrient availability. When climatic conditions became less favourable the lack of water and competition for nutrients would have resulted in increased competition between these plants and many would have died. Foden et al. [41] showed that the temperature increase in Namibia during the last two or three decades is roughly three times more than the global mean temperature increase reported for the 20th century. Several authors [42–44] showed that drastic temperature increases and drier conditions were present in southern Africa on a number of occasions during the last few centuries. It is further proposed that the decomposition of the dead plants altered the chemical properties of the sand which manifested in the hydrophobicity of FC soil. Various other compounds also entered the soil from the decaying euphorbias, some of which posed phytotoxic/allelopathic and antimicrobial activity. Most of these compounds would probably have broken down in a relative short time, but the milky latex that adhered to the sand became hard and could persist in the soil for a long time. These changes to the soil caused the formation of more FCs and as the transition proceeded from mixed to FCs-only sites, it resulted in a more regular/uniform spatial pattern.

It has been observed that seeds germinate inside FCs and seedlings emerge after good rainfall but survive only for short periods after the rains. As the harsh desert conditions set in, we propose that the seedlings in the FCs die due to the soil water that infiltrated to depths beyond the reach of the young seedlings’ roots. In time, with the occasional rain, the effect left in the soil by euphorbias will slowly erode away, seedlings will survive for longer periods in the older FCs until they are eventually fully established and reach maturity.

This study has shown that the *Euphorbia* theory can explain the formation of fairy circles at specific sites across Namibia where these succulents co-occur with fairy circles. Further research in areas where big populations of the large succulent euphorbias are currently not present, like Namib Rand Nature Reserve and Marienfluss, is required to shed more light on the question if the *Euphorbia* theory is applicable to all the FCs of Namibia, South Africa and Angola.

## Materials and methods

### Physical and chemical properties of FC soil

#### Physical properties, wettability and infiltration time of soil

Five surface soil samples were collected from three separate locations in Namibia (Giribes Plain, Brandberg and Garub (see Additional file 1: Text S5). These are locations where FCs co-occur with *E. damarana* and *E. gummifera*. Particle size analysis of FC soil was performed to identify the sizes of sand, silt and clay fractions (see Additional file 1: Text S5).

The infiltration time of water through soil samples from these locations was determined by developing a laboratory method. Soil (50 g) was placed on filter paper disks in 60 ml plastic syringes fitted to a vacuum chamber. Distilled water (20 ml) was added to the soil samples (5 replicates) in the syringes and allowed to equilibrate for approximately 20 s before application of a low negative pressure (10 kPa). The time it took for the water’s meniscus to reach the surface of the soil was determined (infiltration rate (time/20 ml water)). Soil samples of FCs, the matrix, underneath dead *Euphorbia* plants, and also matrix soil coated with latex were assessed. Latex from *E. damarana* was collected at Brandberg and left to dry at room temperature for about 1 year, whereafter it was powdered in a grinder and mixed in water with matrix soil (2.5, 5.0 and 10.0% m/m latex) on a magnetic stirrer. After drying for 3 weeks at 50 °C the infiltration time was also determined.

Soil wettability (hydrophobicity) was determined by employing the WDPT method [45]. This method was employed on soil from FCs, matrix and under decaying *E. damarana* and *E. gummifera* from the three areas (5 replicates). Soil particles larger than 2 mm were removed by sieve and the average penetration time of 4 water droplets per soil sample was used to determine the level of the hydrophobicity.

#### Soil extraction and GC-MS analysis

Surface soil of Giribes from FCs, matrix and from underneath dead *E. damarana* plants were extracted (50 g) using a speed extractor (Büchi E-916) as previously described for soil extraction in Garub by Meyer et al. [12]. *E. damarana* occurs in Giribes and Brandberg and therefore soil from Brandberg was not analysed (results from Garub have previously been published [12]). The only alteration being the use of isopropanol:ammonia (95:5) as the solvent system [46]. Extracts were methylated and analysed on a GC-MS TQ8040 (Shimadzu) (see Additional file 1: Text S6). Chromatograms were analysed by multivariate analyses to identify discriminatory signals by creating Sigma plots (S-plots). From these S-plots a number of variables were identified with the highest absolute magnitude of variance. Once identified, the retention times were investigated on chromatograms in order to determine whether a difference in chemical constituents between matrix, FC and DP soil extracts were present.

### Phytotoxic and antibacterial activity of *E. damarana* and *E. gummifera*

#### Plant collection and extraction

Aerial parts of living *E. damarana* and *E. gummifera* were collected from Giribes and Garub, respectively, placed in paper bags, transported and stored at − 80 °C for 2 days, followed by storage at 5 °C until extraction in methanol (see Additional file 1: Text S7). Due to the poisonous nature of these plants, caution was taken during harvesting and laboratory analyses. The dominant grass species of Garub, *Stipagrostis uniplumis*, was harvested with intact roots and rhizosphere, placed in airtight plastic ziplock bags and stored at 5 °C after collection and transport.

#### Germination inhibition assay

The effect of *E. gummifera* extract (0.625 to 20.0 mg/ml dilution series) on the germination of the seeds of a grass species naturalised in Namibia, *Eragrostis tef,* were determined on filter paper disks in Petri dishes moistened with either 1 or 2 ml water (see Additional file 1: Text S8).

#### Rhizosphere bacterial isolation and identification

Attached rhizosphere soil particles were removed from the roots of *S. uniplumis* and bacterial identification was done using 16S rRNA sequencing [47] (see Additional file 1: Text S9).

#### Microtiter-based antibacterial assay

The antibacterial properties of *E. gummifera* and *E. damarana* methanol extracts were tested in microtiter plates [48] against 6 unidentified and 2 identified *S. uniplumis* rhizosphere bacteria and described in Additional file 1: Text S10.

#### Chemical analysis of *E. gummifera* methanol extract

The *E. gummifera* methanol extract was chromatographed on silica gel with elution solvent mixtures of increasing polarity of hexane, ethyl acetate and methanol. Due to the solidifying of latex in the extract at room temperature, the chromatography glass column was heated to 40‒45 °C with 3 hair dryers and eluent solvents in a water bath (40‒45 °C). Collected fractions were analysed by GC-MS for compound identification (see Additional file 1: Text S6).

### Spatial point pattern analysis

#### Study area selection and experimental design

The spatial distribution of FCs and two *Euphorbia* spp. were conducted at: (1) FC-only sites, (2) *E. damarana* populations, (3) *E. gummifera* populations, and (4) areas where FCs co-occur with one of the two plant species. Several localities of co-occurrence were identified and are described in the Additional file 1: Text S11, Figs. S14‒S17. These co-occurrence areas are all on sandy soil and are named ‘mixed sites’ in this study. The mixed sites where FCs and *E. damarana* (in northern Namibia) or *E.* *gummifera* (in southern Namibia) co-occur, are hypothesised to be transitioning from high numbers of euphorbias and low numbers of FCs, to areas where the plants are becoming less and the FCs becoming more, up to a point in the future when only FCs will be present.

Study sites were chosen in areas where the terrain was homogeneous and free of obstructions such as dry riverbeds that could potentially introduce bias into the analysis. The sampling design ensured that independence was met by placing sites a minimum distance of 1 km apart, although larger distances were frequently the case. Four locations in Namibia were selected for the analyses: (1) Giribes and (2) Palmwag (both northwestern Namibia), (3) Brandberg (central western Namibia) and (4) Garub (southwestern Namibia). Representative sites of each area were constructed resulting in 19 sites (see Additional file 1: Text S11, Table S6).

#### Data acquisition and analysis

Satellite imagery for the four study sites were obtained from the Digital Globe Foundation (WGS1984-UTM Zone 33S, see Additional file 1: Table S7). ArcMap 10.5.1 was used to create pan-sharpened images of the imagery and the objects in the sites manually digitized. The additional fields: x-coordinate, y-coordinate and perimeter (for size comparison), were added to the attribute tables of each site.

On-screen digitizing was done to capture the location of the objects. From the 19 sites a total of 12,516 objects were digitized. These consisted of 5095 FCs, 4763 *E. damarana*, 2167 *E. gummifera* and 491 sand circles in rocky areas [49] (see Additional file 1: Text S11.2). After all the objects in the site were digitized, their perimeter was calculated.

#### Size comparison of FCs, *E. damarana* and *E. gummifera*

Perimeter size comparisons were done for all the FCs, *E. damarana* and *E. gummifera* plants at Brandberg and Garub, respectively, using a *t* test. Only 69 *E. damarana* plants were identified in the mixed site at Giribes and since this sample was so small, a size comparison at this location was not done.

#### Point pattern statistics

A number of point pattern analysis techniques were used to compare the spatial distribution of FCs, *E. damarana* and *E. gummifera* in places where they co-occur with FCs (mixed sites). For the Voronoi tessellations analysis, tiles were created around each object and the number of corners of each tile was calculated [50]. The average distance to the nearest neighbour (nn) was also calculated, as well as the distance to the nn ratio; the latter calculated using the Clark-Evans test (see Additional file 1: Text S12) [51]. To reveal the degree of smaller-scale order effects in the patterning of FCs, *E. damarana* and *E. gummifera*, the pair-correlation function g(r) was used [52] (see Additional file 1: Text S13). The L-function was used to assess departures from complete spatial randomness at larger distances [53] (see Additional file 1: Text S14). All analysis was performed in R using Spatstat and the Dirichlet packages.

### Historical image comparison

FCs and dead *Euphorbia* plants marked by Theron, as well as his historical images of Giribes in 1978 [13] were inspected on site in 2016. Obtaining historical aerial images of the other study site locations was challenging, as fine resolution images were only available for a few areas. Aerial imagery of Garub had the finest resolution and dated back to 1966, which was used in the image comparison. Thirty-two *E. gummifera* plants and 18 FCs in the Garub aerial image were ground-truthed on location.

Although the aerial image of Garub had a fine enough resolution to clearly observe *E. gummifera* plants, the contrast was not good enough for FCs to be visible. The exact location of individual *E. gummifera* plants was determined by comparing the 1966 image to a Google Earth™ image of 2011, and ground observations in 2016. Where ground control points, for instance rocky outcrops, streams or other similar landmarks could be identified in both images, they were used to georeference screen grabs of the 1966 image.

### Site suitability analysis to create a FC prediction map

#### Defining the criteria

A site suitability analysis for FCs in Namibia was undertaken using altitude, rainfall and land cover (sandy grassland). The data of the environmental factors in Namibia was available in electronic format and entered into ArcMap (version 10.5 ESRI) to create the suitability map. This study focused on Namibia although we are aware that FCs also occur in Angola and South Africa.

FCs are generally confined to areas with a MAP of 80‒120 mm [2]. However, a conservative approach was followed, and the precipitation range of 50‒150 mm MAP was used [3, 23]. Altitude is an important predictor variable of FCs. A Google Earth™ investigation into where FCs occur and a previous review [1] led to the decision to restrict the altitudinal range from 500 to 1200 mamsl.

Fairy circles most often occur in grasslands on sandy soil. In this study these two variables were combined into one predictor variable. Landsat™ 5 imagery was previously used to identify sandy deposits in Namibia [54]. In the current analysis Landsat™ imagery was also used but first subjected to an unsupervised image classification to group the pixels into different land cover types based on similar spectral properties. From this classification land cover classes corresponding to grassland on sandy deposits were determined. An example of the vegetation classification process in Giribes is described and shown in Additional file 1: Text S15 and Fig. S18.

#### Data acquisition and creating the model

All data used in the study was freely available in electronic format and more information about it is provided in the Additional file 1: Text S15 and Table S8. The steps in the creation of the ArcGIS model are described in Additional file 1: Text S16, Figs. S19 and S20.

#### Model validation

The resultant map was then validated by creating a positive control of 100 random sample points in Namibia and inspecting them using Google Earth™ for the presence/absence of FCs. A buffer of 10 km was constructed around each sample point and the resultant polygons exported as a *.kml file. This was then manually inspected in Google Earth™ and a number of areas were ground-truthed for the presence/absence of FCs. When there were more than 10 bare circular patches in the same area with a minimum size of 2 m, they were considered to constitute the presence of FCs.

A negative control of the relative same size as the modelled FC distribution was created with a calculated 83 random sample points with an 8 km buffer in an area where none of the predictor variables were present (see Additional file 1: Text S17, Fig. S20). These points were then investigated for the presence/absence of FCs with Google Earth™.

The historical imagery tool had to be used in Google Earth™ as the coverage and quality of the imagery varied both in terms of spatial and temporal resolution; i.e. certain sites had several years of imagery and others only had 1 or 2 years of coverage (sometimes taken during the dry periods when grass was absent). Other sites were not covered with imagery of adequate spatial resolution.

#### Comparison of FC prediction map with distribution of *Euphorbia* species

The distribution maps for *E. damarana*, *E. gummifera* and *E. gregaria* were obtained from the Tree Atlas Project of Namibia [55] and from the South African National Botanical Institute (SANBI). The data were collected by grid cell (15′ × 15′ or quarter-degree square, approx. 27 × 27 km). Each layer was overlaid onto the modelled FC distribution, and also intersected with the modelled FC distribution.

## Supplementary information

**Additional file 1.** Additional text, Figs. S1 to S20, Tables S1 to S8, References.

**Additional file 2: Movie S1.** Water drop adsorption time on soil from the matrix, FC and under dead *E. damarana.* This is an indication of hydrophobicity and adsorption, not infiltration to deeper soil levels.

## Data Availability

The datasets used and/or analysed during the current study are available from the corresponding author on request and available at: https://drive.google.com/drive/folders/1d8tgRhQhhVhnGPWkBBtVfbbew-tDL6Dl?usp=sharing.

## Abbreviations

CSR: Complete spatial randomness
ddH_2_O: Double distilled water
DMSO: Dimethylated sulfoxide
DP: Dead *Euphorbia* plant
FC: Fairy circle
g(r): Pair correlation function
GC-MS: Gas chromatography-mass spectroscopy
GIS: Geographic information systems
GPS: Global positioning system
mamsl: Metre above mean sea level
MAP: Mean annual precipitation
MIC: Minimum inhibitory concentration
nn: Nearest neighbour
PCA: Principal component analysis
R-value: Nearest neighbour index
SANBI: South African National Botanical Institute
SD: Standard deviation
S-plot: Sigma plot
USGS: United States Geological Society
WDPT: Water droplet penetration time
