# Supplementary material for: The allelopathic, adhesive, hydrophobic and toxic latex of Euphorbia species is the cause of fairy circles investigated at several locations in Namibia
Source: BMC Ecol. 2020 Aug 3;20:45. doi: 10.1186/s12898-020-00313-7 (PMC7397633; doi:10.1186/s12898-020-00313-7)
Supplement: Supplementary file 1 — Additional file 1. Additional text, Figs. S1 to S20, Tables S1 to S8, References. [file 12898_2020_313_MOESM1_ESM.pdf]

# Additional Information for:

## The allelopathic, adhesive, hydrophobic and toxic latex of *Euphorbia* species is the cause of fairy circles investigated at several locations in Namibia

J. J. Marion Meyer, Christiaan E. Schutte, Jan W. Hurter, Nicole S. Galt, Petunia Degashu, Greg Breetzke, Denis Baranenko and Nicole L. Meyer

J. J. Marion Meyer
Email: marion.meyer@up.ac.za

**This PDF file includes additional files for:**

Additional text

Additional Figs. S1 to S20

Additional Tables S1 to S8

Additional references

Additional movie file S1 (Water drop adsorption time on soil from the matrix, FC and under dead *E. damarana*. This is an indication of hydrophobicity and adsorption, not infiltration to deeper soil levels.)

Data files available at:
https://drive.google.com/drive/folders/1d8tgRhQhhVhnGPWkBBtVfbbew-tDL6Dl?usp=sharing

## 1. Physical and chemical properties of soil

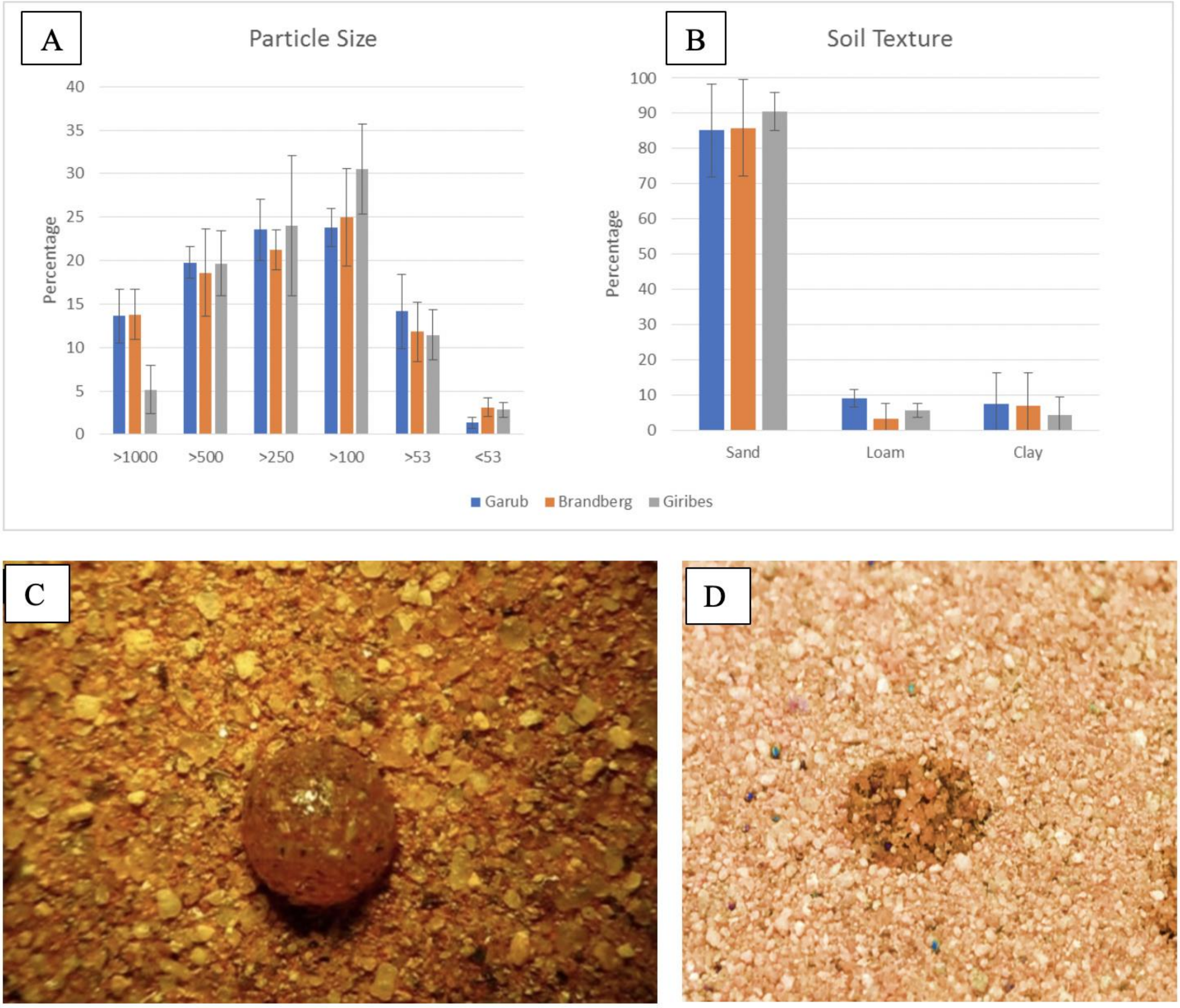


**Fig. S1.** Soil particle sizes (**A**) and texture (**B**) of FCs at Garub, Brandberg and Giribes. Soil wettability: water drop placed on FC soil displaying strong hydrophobic characteristics (**C**), and on matrix soil (**D**) (an additional movie file shows this, see Additional Movie File S1)

**Table S1.** Soil wettability in Giribes, Brandberg and Garub as determined with the water droplet penetration time assay [1]

| Collection sites | % of samples classified according to penetration time | | | |
|---|---|---|---|---|
| | Penetration time (s) | | | |
| | <1 | 1-10 | 11-60 | >60 |
| Matrix | | | | |
| Giribes | 100 | 0 | 0 | 0 |
| Brandberg | 100 | 0 | 0 | 0 |
| Garub | 100 | 0 | 0 | 0 |
| Fairy circle | | | | |
| Giribes | 50 | 50 | 0 | 0 |
| Brandberg | 0 | 50 | 50 | 0 |
| Garub | 25 | 25 | 50 | 0 |
| Dead plant | | | | |
| Giribes | 0 | 0 | 0 | 100 |
| Brandberg | 0 | 0 | 75 | 25 |
| Garub | 0 | 0 | 25 | 75 |

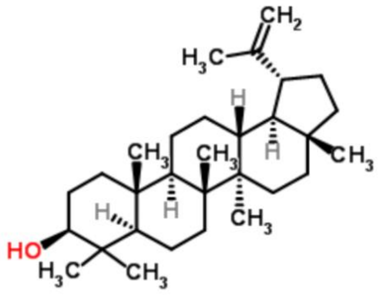

Lupeol

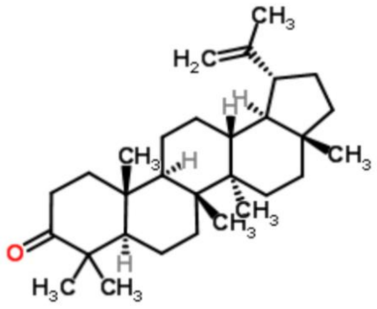

Lupenone

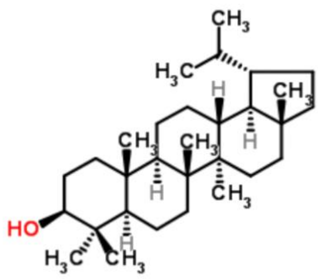

Lupanol

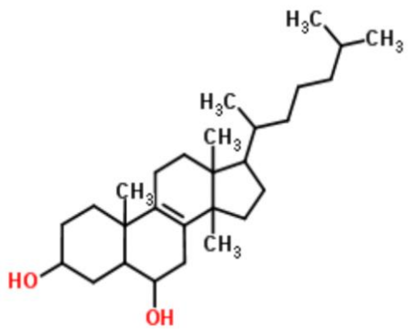

Macdougallin

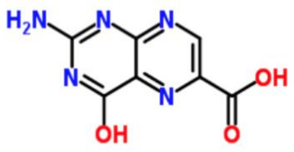

Pterin-6-carboxylic acid

**Fig. S2.** Compounds identified in soil samples that were previously identified in *Euphorbia* species and not in grass species

## 2. Phytotoxic and antibacterial activity of *E. gummifera* and *E. damarana*

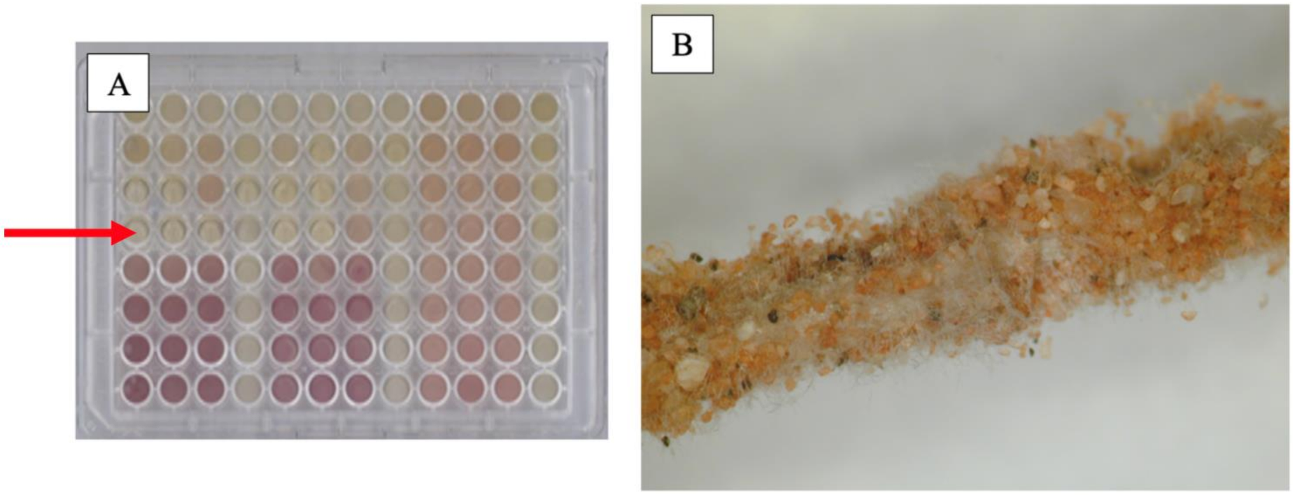


**Fig. S3.** Antibacterial activity (**A**) of *E. damarana* extract on two of the unidentified root rhizosphere (**B**) bacteria at 0.31 mg/ml extract (arrow). Each set of three vertical lanes represents three replicates of serially diluted *E. damarana* extract on an unidentified root rhizosphere bacterium. Red-coloured wells represent no inhibition

**Table S2.** Compounds extracted from *E. gummifera* with previous reported antibacterial activity

| Compound name | Compound structure | Hit % | Retention time (min) |
|---|---|---|---|
| Lupeol | 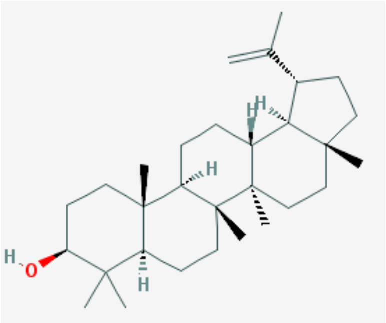 | 90% | 27.755 |
| α-Amyrin | 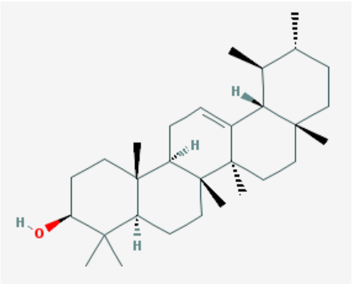 | 92% | 31.860 |
| β-Amyrin | 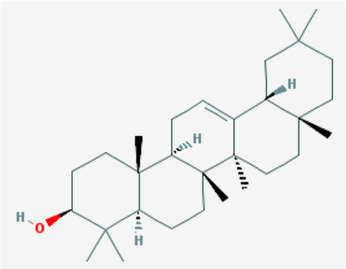 | 94% | 31.890 |
| Betulin (Lup-20(29)-ene-3, 28-diol, (3.beta)) | 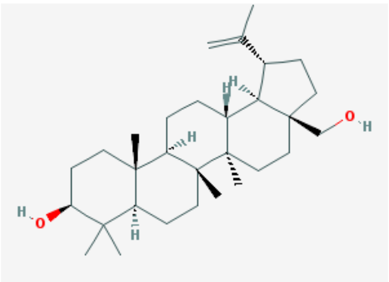 | 89% | 28.485 |
| Lupeol acetate (Lup-20(29)-en-3-ol, acetate, (3.beta)) | 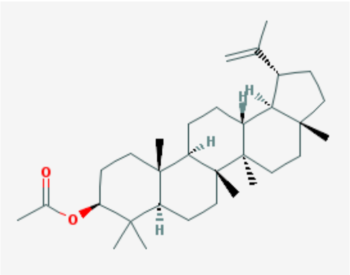 | 94% | 24.030 |

| Compound name | Compound structure | Hit % | Retention time (min) |
|---|---|---|---|
| Lanosterol | 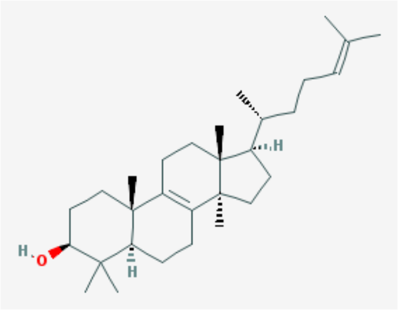 | 90% | 31.375 |
| 1-Heptacosanol | 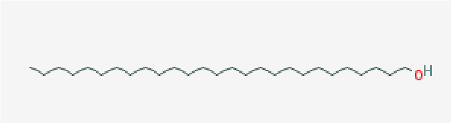 | 97% | 24.555 |
| Quinic acid | 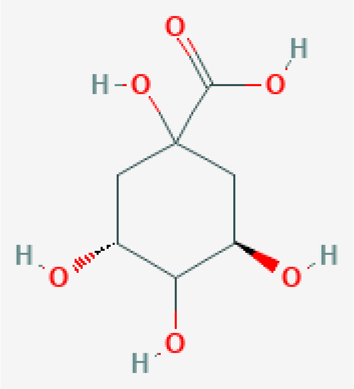 | 83% | 15.280 |
| Lucenin 2 | 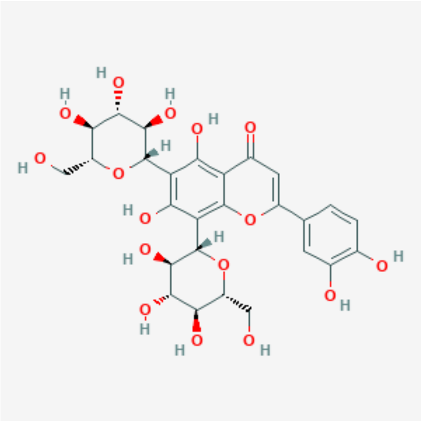 | 82% | 24.230 |

## 3. Spatial analysis

**Table S3.** Sample site type and mean perimeter (no significant statistical differences were found between the perimeter sizes of FCs and euphorbias occurring in the same locations, $p < 0.05$)

| **Sample site** | **Site type** | **Mean perimeter (m)** |
|---|---|---|
| Brandberg | | |
| C1 | Fairy circles | 18.93 |
| C2 | Fairy circles | 17.41 |
| M1 | Fairy circles | 15.77 |
| M2 | Fairy circles | 13.77 |
| P1 | *E. damarana* | 16.47 |
| P2 | *E. damarana* | 17.46 |
| M1 | *E. damarana* | 16.25 |
| M2 | *E. damarana* | 16.13 |
| Garub | | |
| C1 | Fairy circles | 10.59 |
| C2 | Fairy circles | 9.30 |
| M1 | Fairy circles | 12.98 |
| M1 | Fairy circles | 16.48 |
| P1 | *E. gummifera* | 8.50 |
| P2 | *E. gummifera* | 8.16 |
| M1 | *E. gummifera* | 16.06 |
| M2 | *E. gummifera* | 16.75 |

**Table S4**. Results of the Voronoi tessellations

| Sample site | Site type | Mean number of corners | % with 6 corners |
|---|---|---|---|
| Giribes | | | |
| C1 | Fairy circles | 5.91 | 38.10 |
| C2 | Fairy circles | 5.89 | 33.12 |
| M1 | Mixed | 5.87 | 35.68 |
| Palmwag | | | |
| P1 | *E. damarana* | 5.91 | 30.66 |
| P2 | *E. damarana* | 5.92 | 18.66 |
| M1 | Mixed | 5.91 | 33.66 |
| M2 | Mixed | 5.93 | 20.60 |
| Brandberg | | | |
| C1 | Fairy circles | 5.91 | 39.07 |
| C2 | Fairy circles | 5.88 | 37.38 |
| M1 | Mixed | 5.93 | 34.17 |
| M2 | Mixed | 5.92 | 35.50 |
| P1 | *E. damarana* | 5.92 | 30.04 |
| P2 | *E. damarana* | 5.92 | 32.98 |
| Garub | | | |
| C1 | Fairy circles | 5.91 | 36.21 |
| C2 | Fairy circles | 5.93 | 36.30 |
| M1 | Mixed | 5.88 | 33.22 |
| M2 | Mixed | 5.84 | 28.98 |
| P1 | *E. gummifera* | 5.93 | 30.08 |
| P2 | *E. gummifera* | 5.91 | 30.32 |

**Table S5.** The mean distance to nearest neighbour (nn) and the distance to nearest neighbour ratio (R-value). R-value > 1 indicates regularity, R-value = 1 complete spatial randomness and R-ratio < 1 indicates clustering

| **Sample site** | **Site type** | **Mean distance to nn (m)** | **R-value** |
|---|---|---|---|
| Giribes | | | |
| C1 | Fairy circles | 17.05 | 1.49 |
| C2 | Fairy circles | 15.74 | 1.42 |
| M1 | Mixed | 14.08 | 1.36 |
| Palmwag | | | |
| P1 | *E. damarana* | 10.17 | 1.05 |
| P2 | *E. damarana* | 7.24 | 1.13 |
| M1 | Mixed | 11.23 | 1.13 |
| M2 | Mixed | 8.55 | 1.20 |
| Brandberg | | | |
| C1 | Fairy circles | 15.31 | 1.45 |
| C2 | Fairy circles | 16.51 | 1.41 |
| M1 | Mixed | 10.10 | 1.29 |
| M2 | Mixed | 10.10 | 1.24 |
| P1 | *E. damarana* | 10.23 | 1.21 |
| P2 | *E. damarana* | 11.34 | 1.21 |
| Garub | | | |
| C1 | Fairy circles | 10.23 | 1.38 |
| C2 | Fairy circles | 9.02 | 1.36 |
| M1 | Mixed | 9.35 | 1.15 |
| M2 | Mixed | 11.93 | 1.11 |
| P1 | *E. gummifera* | 4.78 | 1.08 |
| P2 | *E. gummifera* | 4.92 | 1.05 |

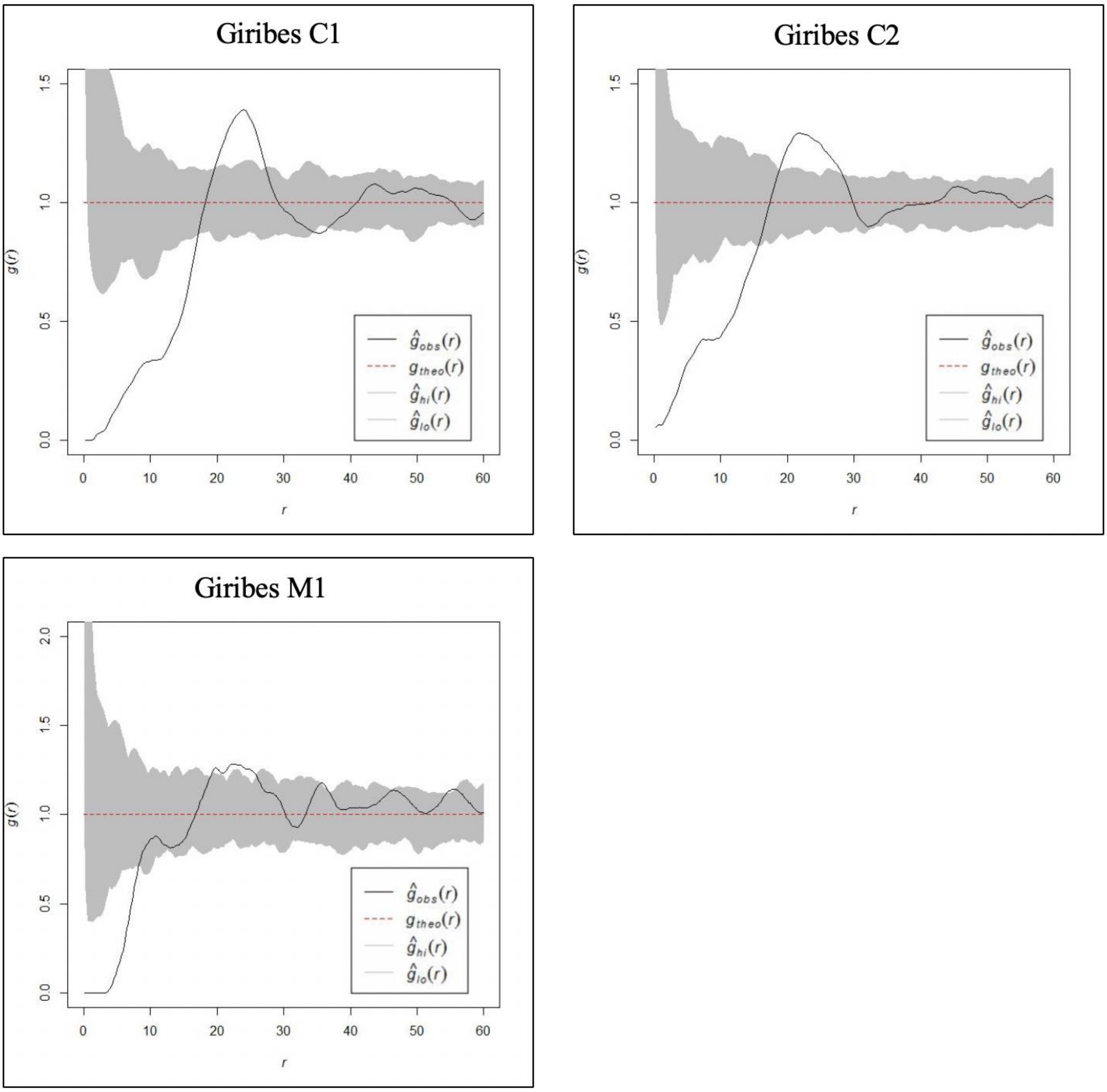


**Fig. S4.** Graphs of the pair correlation function for the fairy circle sites (**C1**, **C2**) and the mixed sample site (**M1**) at Giribes

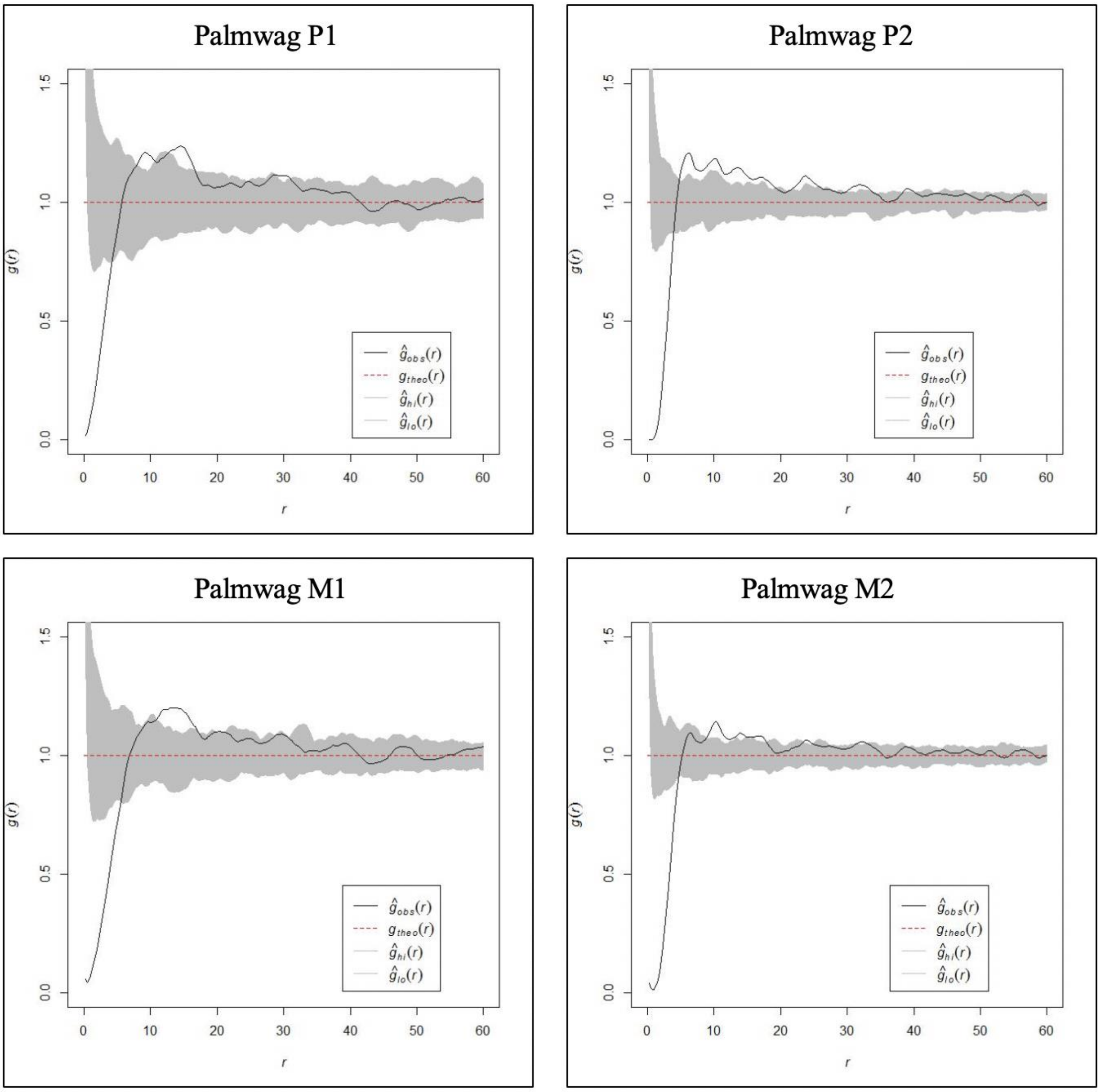


**Fig. S5.** Graphs of the pair correlation function for the *E. damarana* sites (**P1**, **P2**) and the mixed sites (**M1**, **M2**) at Palmwag

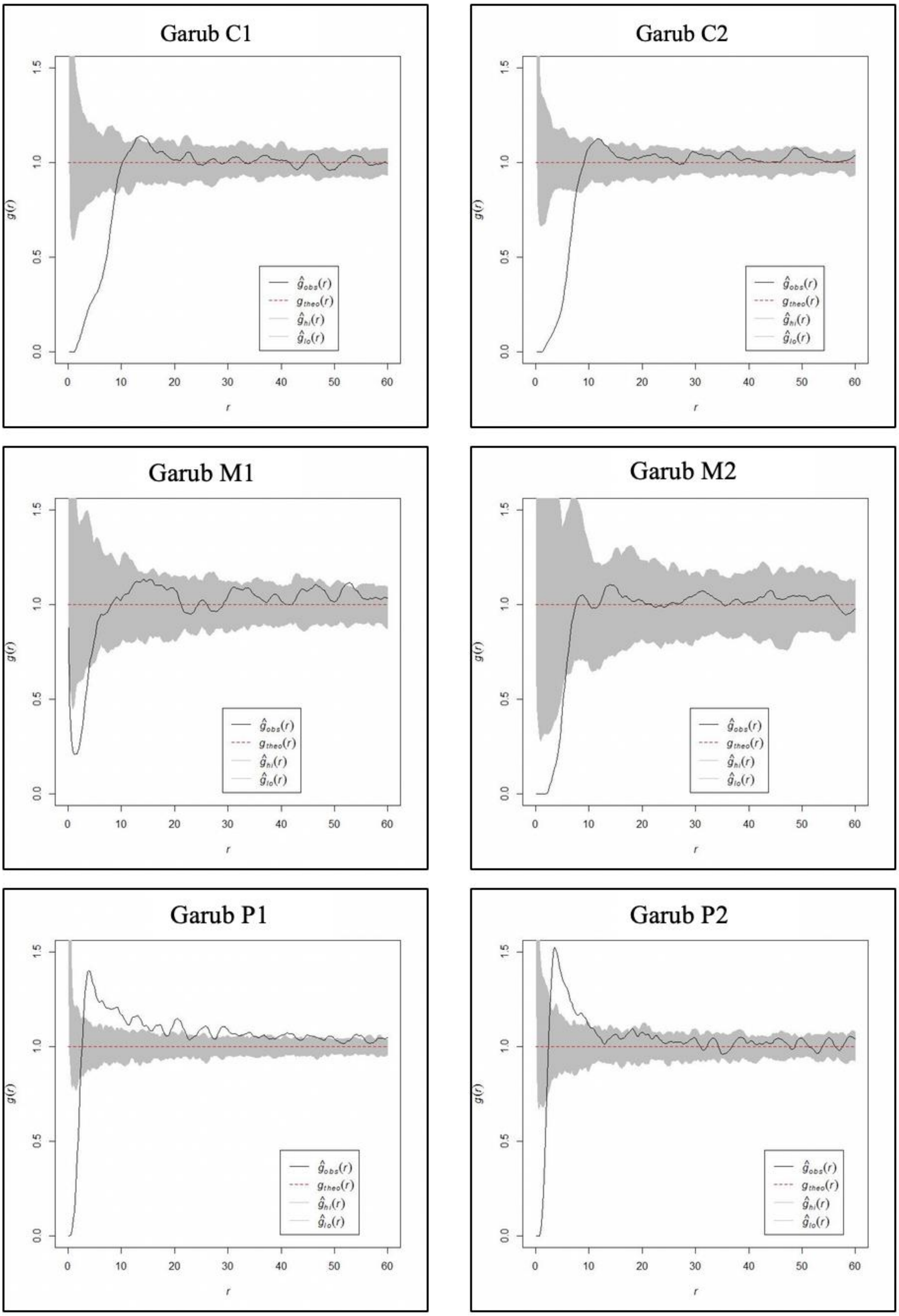


**Fig. S6.** Graphs of the pair correlation function for the fairy circle (**C1**, **C2**), mixed (**M1**, **M2**) and *E. gummifera* (**P1**, **P2**) sites at Garub

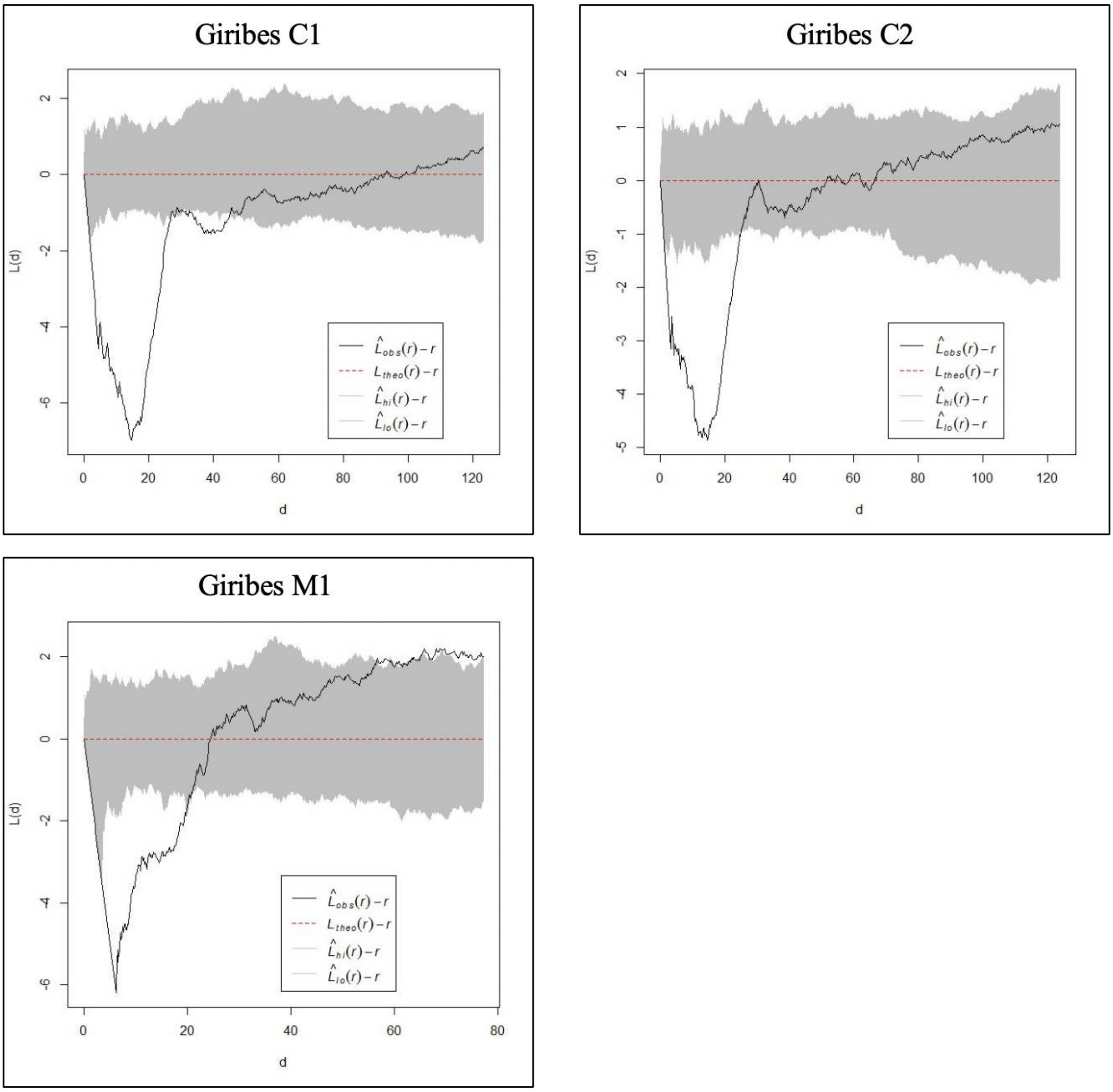


**Fig. S7.** L-function graphs for the fairy circle (**C1**, **C2**) and mixed sites (**M1**) at Giribes Plain

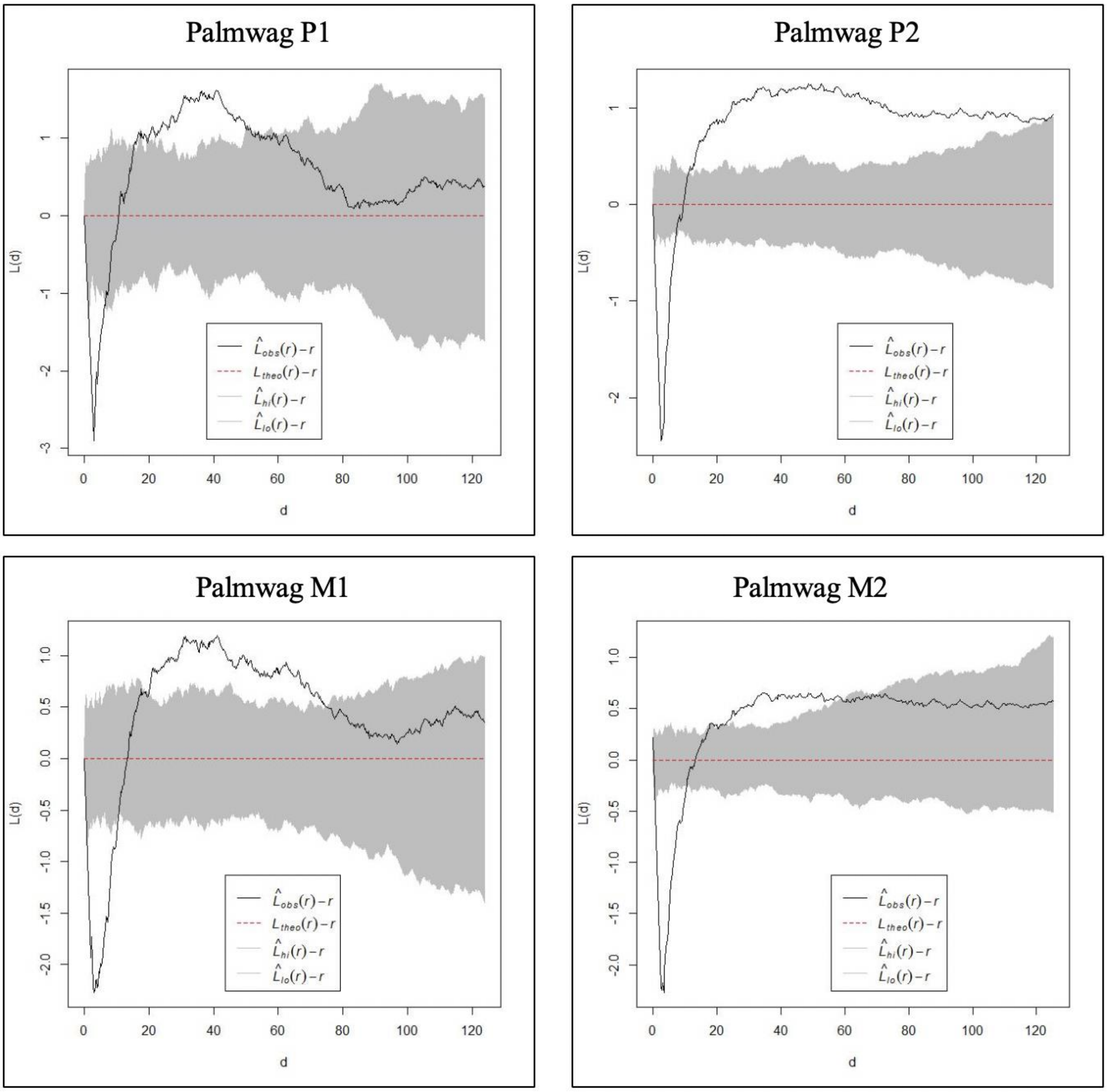


**Fig. S8.** L-function graphs for the *E. damarana* (**P1**, **P2**) and mixed sites (**M1**, **M2**) at Palmwag

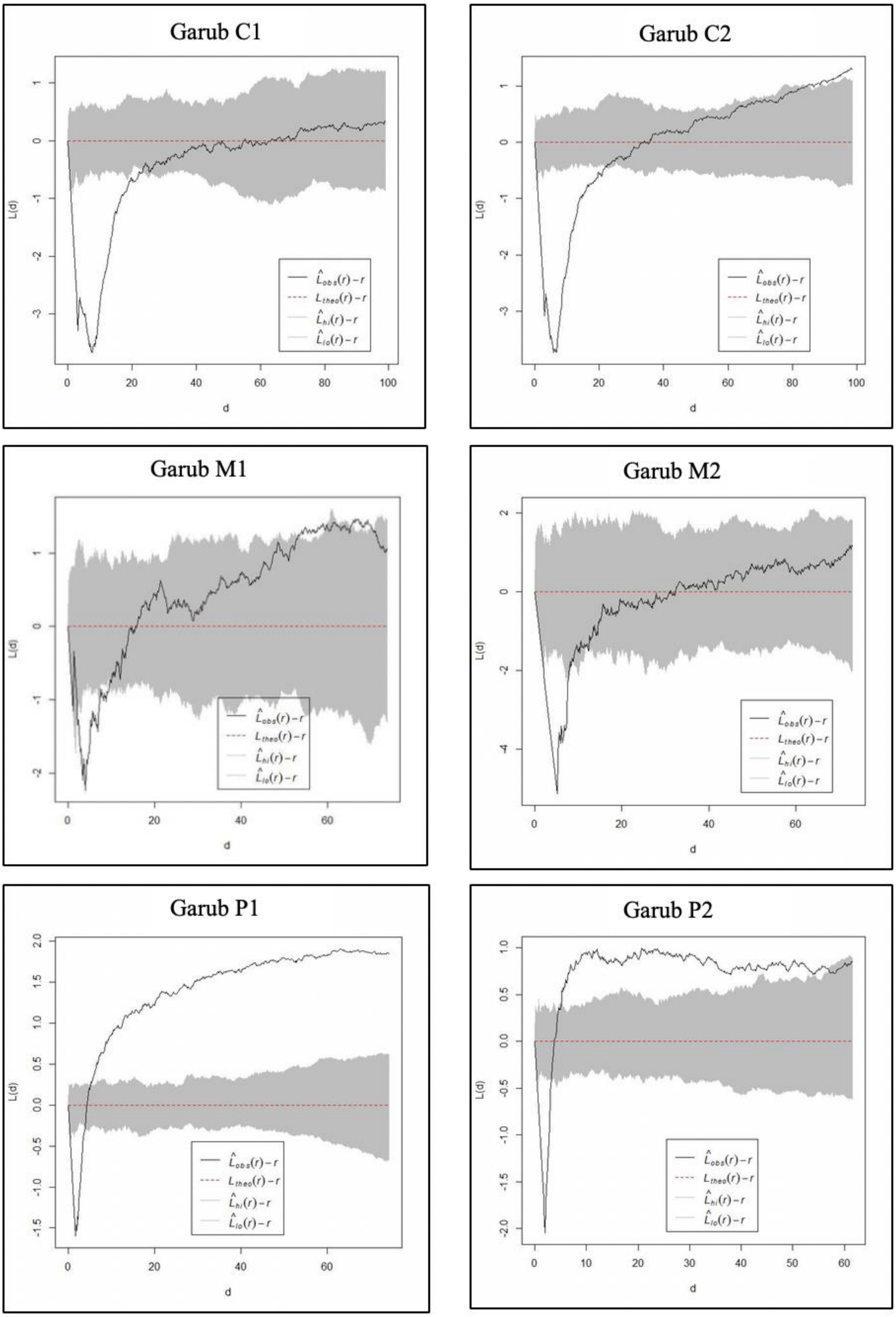


**Fig. S9.** L-function graphs for the fairy circles (**C1**, **C2**), mixed (**M1**, **M2**) and *E. gummifera* sites (**P1**, **P2**) at Garub

## 4. Site suitability analysis for prediction of fairy circle distribution

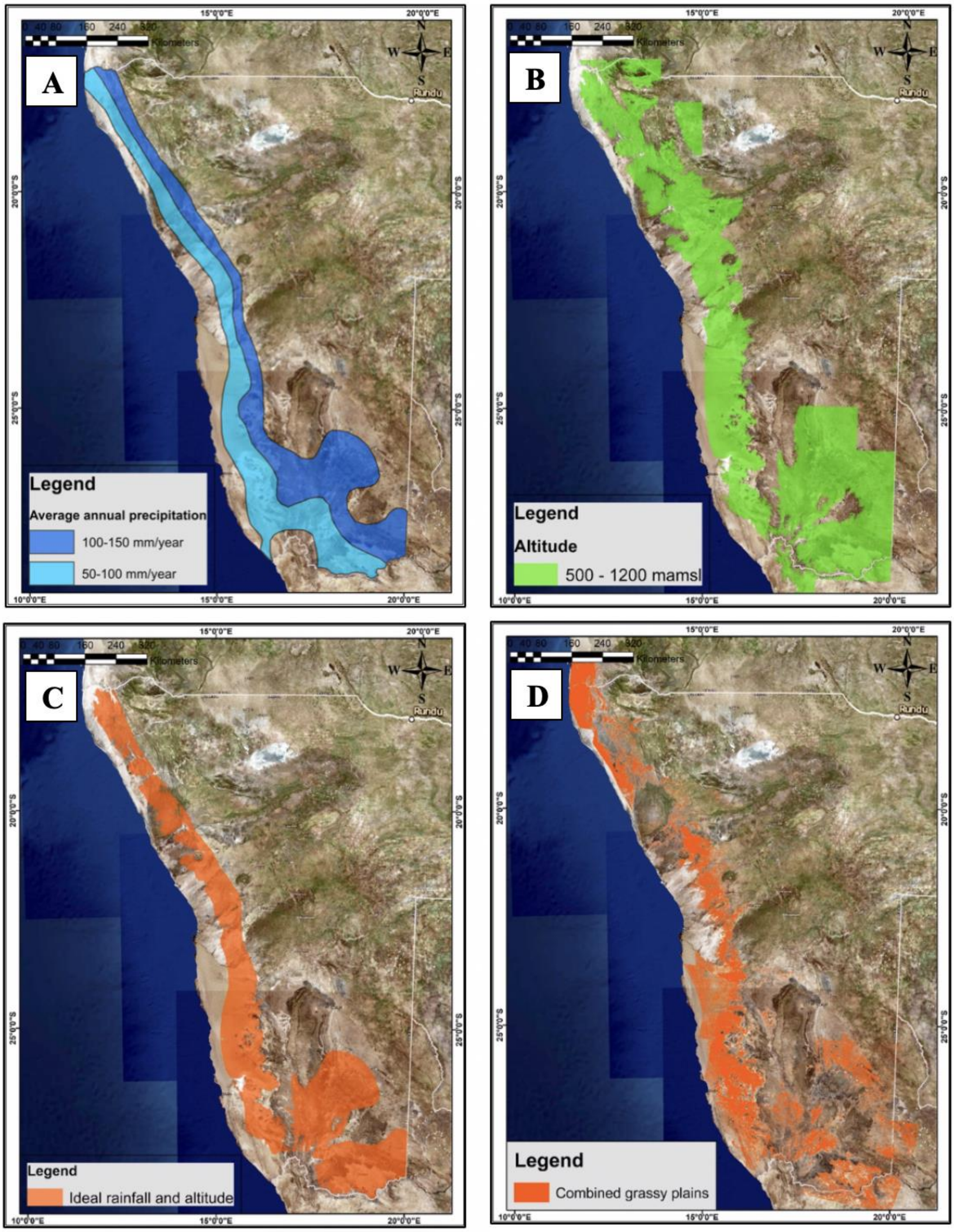


**Fig. S10.** The ideal rainfall range (**A**), altitude range (**B**), the intersection of ideal rainfall and altitude ranges (**C**) and the combined grassy land cover classification map for the study area (**D**). Created in ArcMap

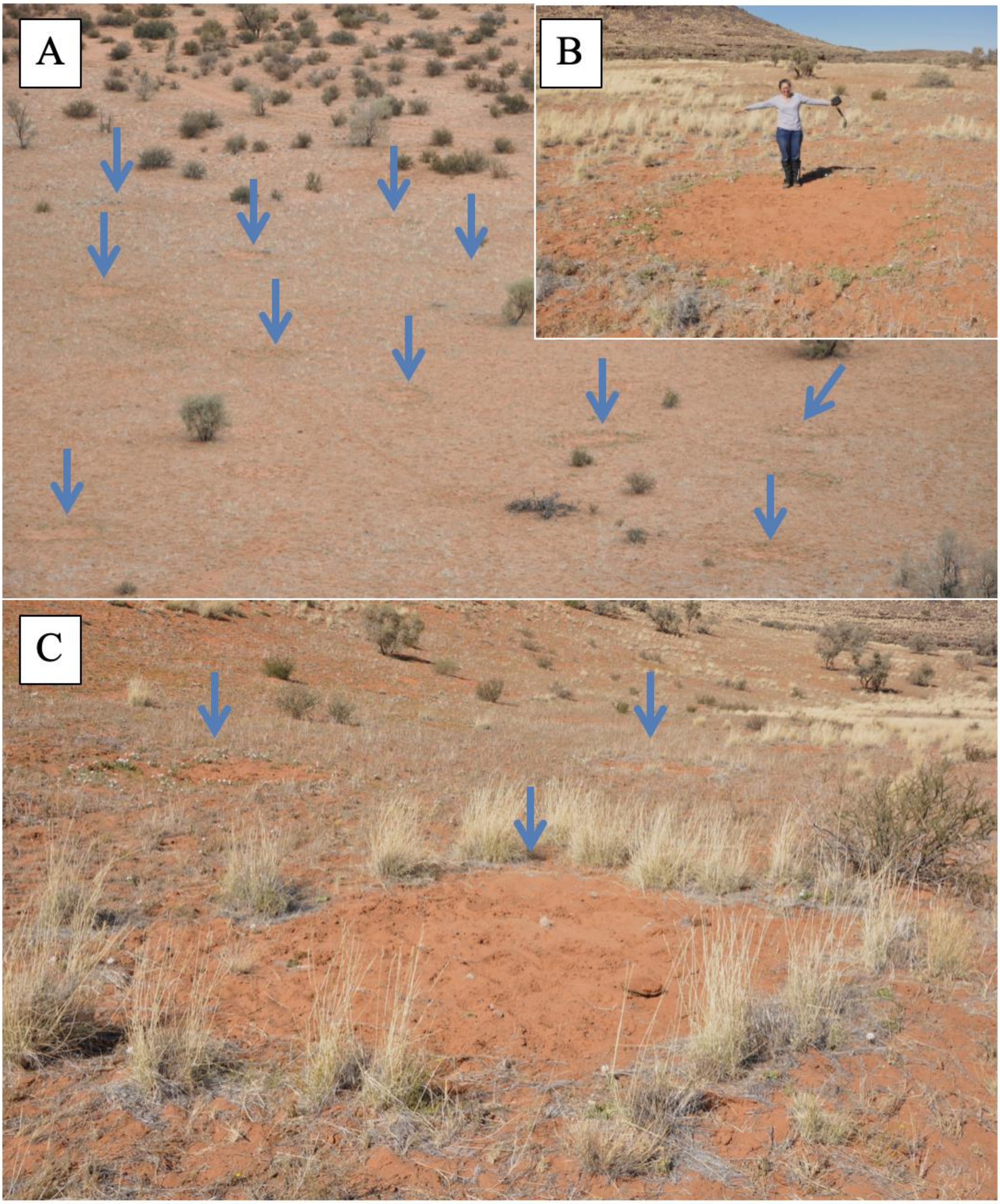


**Fig**. **S11.** Fairy circles in the Bakrivier, Kalahari, South Africa (coordinates 27°58'54.466"S; 20°02'10.913"E). Image (**A**) taken from the high riverbank, where *E. gregaria* occurs, blue arrows indicating FCs in the dry riverbed (**A**). Fairy circles surrounded by *Arctotis leiocarpa* Harv. (**B**) or tall grasses (**C**)

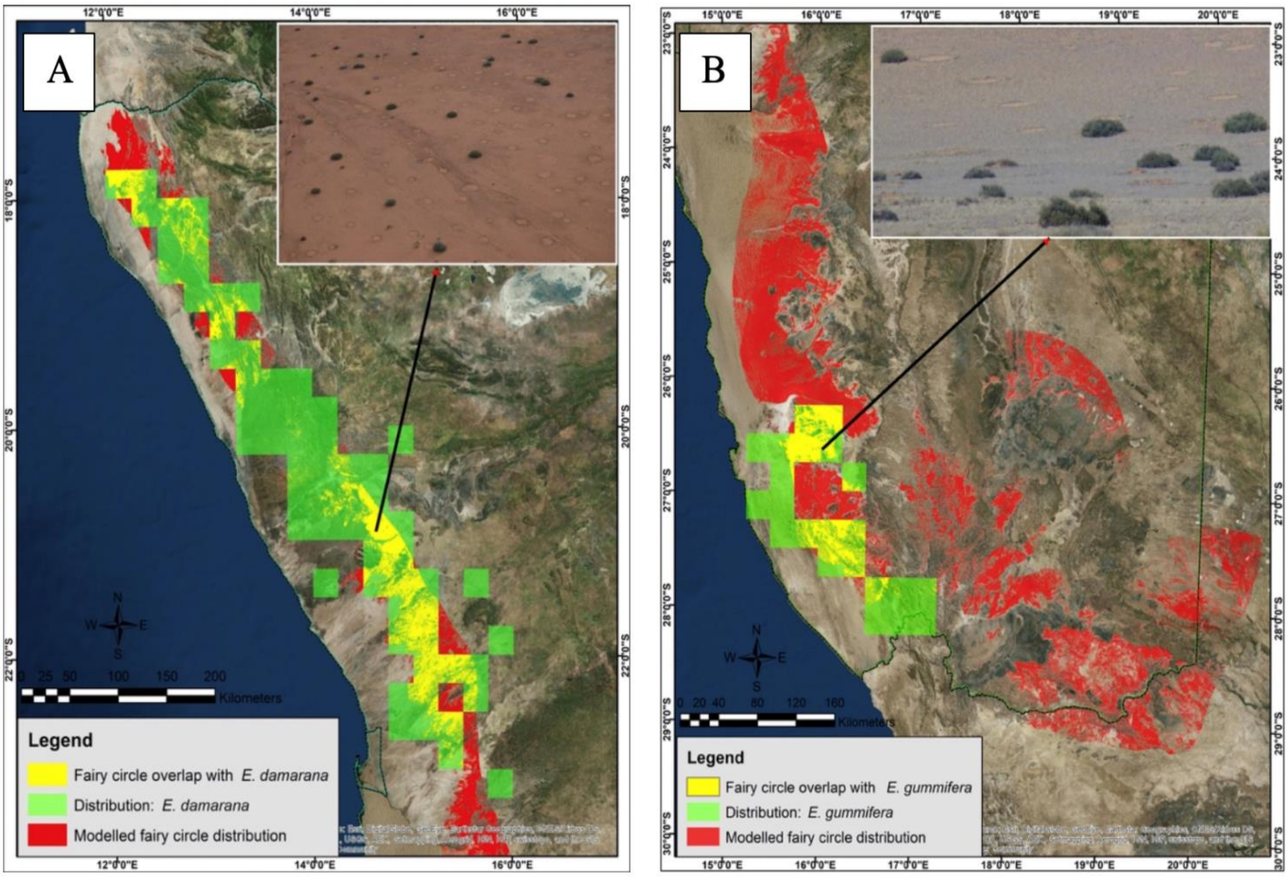


**Fig. S12.** *E. damarana* (**A**) and *E. gummifera* (**B**) distribution in relation to the modelled fairy circle distribution. The yellow areas indicate areas where the species overlap with the modelled fairy circle distribution. Examples of fairy circle co-occurrence with *E. damarana* at Brandberg, northern Namibia (insert **A**) and with *E. gummifera* at Garub, southern Namibia (insert **B**). Created in ArcMap

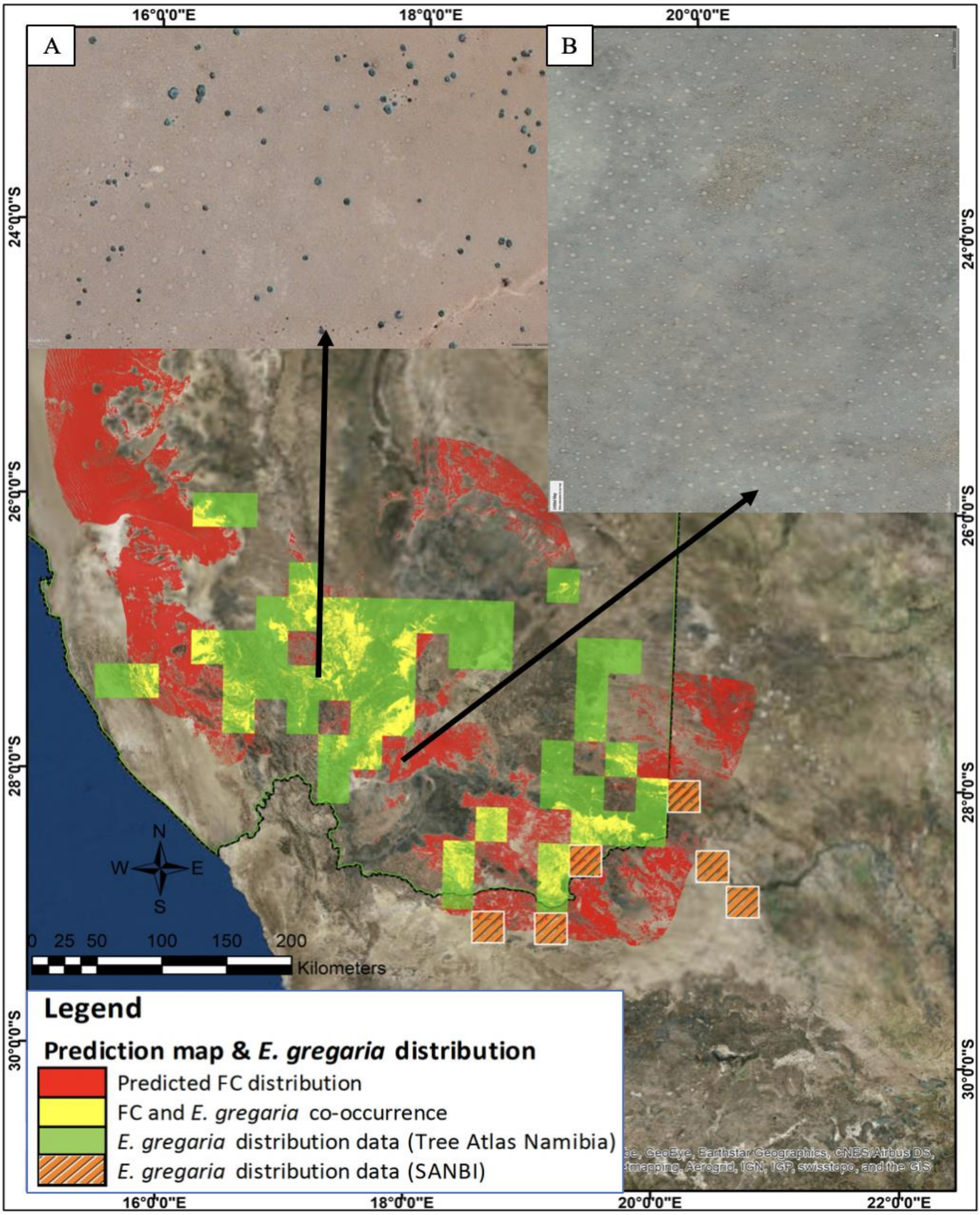


**Fig. S13.** *E. gregaria* distribution in relation to the modelled FC map. Yellow areas indicate where the distribution of *E. gregaria* overlaps with the modelled FC distribution. Newly documented FCs co-occurring with *E. gummifera* southwest of Keetmanshoop (**A**, source Google Earth™) (27°15'32.828"S; 17°16'45.498"E). Thousands of newly documented FCs south of Grünau (**B**, source Google Earth™) (28°02'44.074"S; 18°06'42.055"E). Created in ArcMap

## 5. Physical properties of fairy circle soil

Soil samples (at least 5) were collected from locations where FCs (fairy circles) co-occur with *Euphorbia damarana* and *E. gummifera* at Giribes Plain (19°03'47.05"S; 13°20'21.31"E), Brandberg (21°04'26.85"S; 14°38'48.37"E) and Garub (26°38'52.17"S; 16°05'5.95"E). The samples were put through sieves of the following sizes: > 1000 µm, > 500 µm, > 250 µm, > 100 µm, > 53 µm and < 53 µm, to collect different sized particles. Particle size analysis was performed to identify the sand, silt and clay fractions present in each sample [2]. The method comprises two parts, dispersion of the soil and separation of particles into size groups. To allow the soil to disperse completely, the soils were pre-treated to remove organic material and salts. The next step involved removing each particle size group (sand, silt and clay) from the pre-treated soil-water mixture. This was accomplished by allowing the soil particles of different sizes to settle out of the solution at different times. A floating device, with measuring units, was placed in the pre-treated soil-water mixture. Readings were taken at the start and after 6.5 hours to calculate the concentration of sand, clay and silt [2]. The fractions were subsequently dried and weighed.

## 6. Soil extraction and GC-MS analysis

Aliquot amounts (700 µg) of all the soil extracts were made up to 1.0 ml with methanol in GC-MS bottles, followed by the addition of 500 µl of methoxyamine hydrochloride (25–30 wt. % in $H_2O$, Sigma-Aldrich) and incubated for 10 minutes at 75°C. The extracts were analysed by means of a GC-MS TQ8040 (Shimadzu) equipped with an AOC-20i auto injector. Separation was carried out on an Rxi®-5Sil MS (30 m × 0.25 mm ID, film thickness 0.25 µm) column. The injector was rinsed three times pre- and post-run to ensure that no cross contamination occurred. A splitless injection mode was employed at a temperature of 250°C. The initial oven temperature was set at 60°C with a hold time of 0.25 min, after which it was increased to a final temperature of 280°C, at a rate of 8°C/min and then held for 10 min, resulting in a total program time of 37.75 min per sample. High purity helium gas was employed as carrier gas, with a primary pressure of 500–900 kPa. The mass spectrometer detector's start time was set to be 8.10 min in order to prevent saturation. Recording continued until the program was completed. An ion source temperature of 200°C was applied while an interface temperature of 250°C and a solvent cut time of 8.0 min proved to be sufficient. The detector voltage was set at 0.1 kV, relative to the tuning result.

Peaks with intensity > 37 000 were manually integrated on the chromatograms in order to compile a table of peak area and retention time for metabolomic analyses using SIMCA-P v. 14

software (Umetrics, Sweden). After the exclusion of statistical outliers, principal component analysis (PCA) sites were created (Pareto scaling) in order to identify discriminatory signals.

**7. *E. damarana* and *E. gummifera* extraction and antibacterial activity**

The collected aerial parts of *E. damarana* and *E. gummifera* were identified by Ms Magda Nel, HGWJ Schweikerdt Herbarium, University of Pretoria, vouchers PRU 122228 and PRU 124383, respectively. The plants were freeze dried (United Science Pty Ltd Freeze drier) for a week and extracted in methanol using a Speed Extractor (Büchi E-916). The extraction was done at 50°C and 100 kPa with four cycles of extraction using 40 ml steel extraction tubes. The extractions consisted of three cycles of 1 min heat up to 50°C, 15 min hold and 5 min solvent discharge, while the last cycle differed only in terms of the hold, which was for 9 minutes. Samples were collected in 240 ml glass bottles and dried using a Genevac EZ-2 Büchi Plus personal evaporator. A total of about 100 g of plant material was extracted.

**8. Germination inhibition assay**

An *E. gummifera* extract (see Text 7 above) stock solution was prepared using methanol as solvent. The solution was sonicated for 15 min in a heated water bath (40°C) and a dilution series of the following concentrations: 20.0, 10.0, 5.0, 2.50, 1.25 and 0.625 mg/ml were prepared. Each concentration had five replicates. To a Petri dish (9 cm) containing a Whatman no.1 filter paper, 2.0 ml of each concentration and 100% methanol (control) was added. The Petri dishes were left open for 2–3 days to allow the methanol to evaporate from the filter paper. The filter papers were once moistened with either 1.0 or 2.0 ml of distilled water. In each plate 30 *Eragrostis tef* seeds were evenly spaced and the plates incubated at 25°C for 48 hours. A stereo microscope was used to determine how many seeds germinated and the data statistically analysed with Graph Pad Prism (GraphPad Software Inc., San Diego, CA) using a two-way Anova analysis with a 95% confidence interval.

**9. Rhizosphere bacterial isolation and identification**

Attached rhizosphere soil particles were carefully removed with a sterile blade from the roots of *Stipagrostis uniplumis* (HGWJ Schweikerdt Herbarium, University of Pretoria, herbarium voucher PRU 124384). To isolate bacteria from the rhizosphere soil, 1 g of the soil was added to 9 ml of Ringers solution (Merck) and mixed thoroughly by shaking vigorously. A dilution series was then made from this solution. Three replicates of each dilution were plated using the spread plate method,

onto nutrient agar (Merck). The plates were incubated at 30–35°C for 48 hours. Distinct single colonies were identified and streaked out on nutrient agar using the cross-streak method. Each distinct culture was sub-cultured until pure cultures without contamination were obtained. Petri dishes were sealed with parafilm and stored at 4.5°C until used for the antibacterial assay. For the purpose of the antibacterial assay single colonies of each distinct type of bacteria was picked up with a sterile loop and the loop dipped in nutrient broth (Merck). The bacteria were then grown in the liquid media.

To identify isolated bacteria, using 16S rRNA gene sequencing, the method described by Patel [3] was used. The DNA extraction was done using the Zymo Research Quick-gDNA Miniprep kit (The Epigenetics Company). The sequences that were obtained from Sanger sequencing were imported into the BioEdit sequence alignment programme [4] to edit the sequences. The edited sequences were imported into the NCBI BLAST website (https://blast.ncbi.nlm.nih.gov/Blast.cgi), where they were compared to known sequences of bacteria.

**10. Microtiter-based antibacterial bioassay**

Ten mg/ml *E. gummifera* and *E. damarana* methanol extract stock solutions were prepared in 2 ml Eppendorf tubes with 10% dimethylated sulfoxide (DMSO). The extracts were sonicated (DSA Ultrasonic Cleaner) for 15 min to allow complete dissolving of the extracts. Following sonication, 900 µl of dd$H_2O$ was added to the extracts. The positive control was prepared by dissolving 2 mg of the antibiotic, ciprofloxacin in 10 ml of dd$H_2O$.

As a precaution to determine whether or not the solvent DMSO would affect the growth of the tested bacteria, a 5% DMSO solution was prepared which served as a solvent control. The densities of the bacterial cultures were determined by adding 200 µl of sterile broth and bacterial cultures to respective wells of a 96 well plate. The absorbance was read at 600 nm and the cultures were adjusted (adding more broth or bacterial culture) depending on the values. The extracts were serially diluted in the microtiter plates and incubated at 37°C for 48 hours.

The bacterial growth was visualised by adding 40 µl of iodonitrotetrazolium chloride (AMRESCO) to all of the wells and the plate incubated in the dark until a colour change was visible (± 45 min). The minimum inhibitory concentration (MIC) was determined visually by determining at which well/concentration no colour change occurred.

## 11. Spatial analysis – descriptions of the four locations

### *11.1. Giribes Plain*

Giribes Plain is located in northwestern Namibia at 19°03'47.05"S; 13°20'21.31"E, close to the town of Sesfontein (Fig. S14). This is the northernmost location that was analysed and represents two types of sites, one with only FCs and one with a small population of *E. damarana* mixed with FCs, about 12 km south of Leopard Rock, landmark of Giribes Plain. The nearest *E. damarana* plants occur about 5 km from Leopard Rock. Giribes is surrounded by hills but the topography inside the plain itself is flat and the underlying substrate is sand. The plain is covered by grass from the genus *Stipagrostis* with thousands of FCs spread out over the plain. Three sample sites were identified on the Giribes Plain: two 500 x 500 m sites containing only FCs and one of 250 x 250 m containing both FCs and *E. damarana* (the small size of this site is due to its location). The FC-only sites are named Giribes C1 and Giribes C2 and the mixed site Giribes M1 (Table S6).

Field work consisted of verification of the objects being studied remotely. This was done by recording the coordinates of 10 FCs and 10 *E. damarana* plants on the satellite images.

### *11.2. Palmwag*

Palmwag is also in northwestern Namibia, 115 km southwest of the Giribes Plain, at 19°54'22.29"S; 13°58'55.55"E (Fig. S15). This site is on the Etandeka Plateau and the nearest site to the Giribes Plain that could be accessed during field excursions, where large enough *E. damarana* populations occur on a rocky substrate without the presence of FCs. One of the aims of this study was to compare the spatial characteristics of FCs with that of *E. damarana* in non-sandy locations.

This site is situated between rocky outcrops sloping down into plains and the geology/substrate is a desert pavement, which is a surface covered with tightly packed rocks [5]. Fig. S15*B* shows a false colour composite image (band 7, 4, 2) of the area, note the green hue, due to the presence of the desert pavement (coarse gravel plains). Between the *E. damarana*, there are many circular disturbances (sand circles) in the desert pavement (Fig. S15*D*). These disturbances are similar to FCs in that they are also mostly round, but instead of the absence of grass, there is an absence of rocks inside the circle area. The remains of dead *E. damarana* could be seen in several of the sand circles, and have been confirmed by phytochemical analysis as described in Meyer et al. [6]. Because both FCs and sand circles in the rocky areas are hypothesised to be a result of dead *Euphorbia* spp., it was decided to include these sand circles in the mixed sites. Unfortunately, we have never been able to verify if grass could establish inside the sand circles in rocky areas, because during all of our visits no grass cover was ever present.

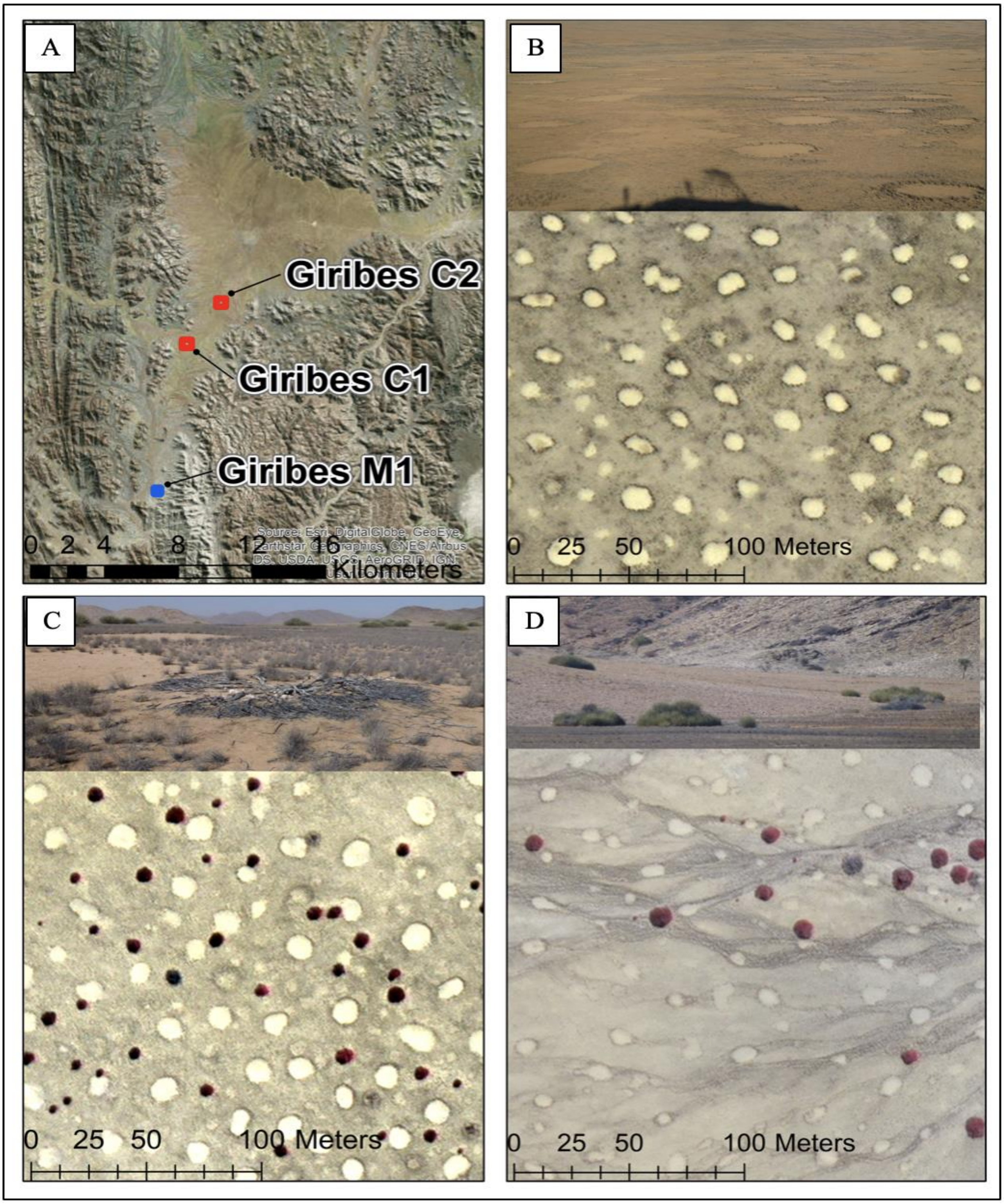


**Fig. S14.** Sampling design at the Giribes Plain (**A**, source Google Earth™). The red squares indicate the two FC sites, while the blue square indicates the mixed site. Fairy circles in the northern parts of Giribes (**B**). Fairy circles co-occurring with *E. damarana* (**C**, **D**); on satellite images bright red indicates healthy vegetation, while dark tones indicate dead vegetation. Satellite imagery in B–D courtesy of DigitalGlobe Foundation

**Table S6.** Naming convention and sizes of the sample sites at four locations in Namibia

| Study site | Type of object | Sample site name | Sample site size (m) |
|---|---|---|---|
| Giribes Plain | Fairy circles | Giribes C1 | 500 x 500 |
| | Fairy circles | Giribes C2 | 500 x 500 |
| | Fairy circles and *E. damarana* mixed | Giribes M1 | 250 x 250 |
| Palmwag | *E. damarana* | Palmwag P1 | 500 x 500 |
| | *E. damarana* | Palmwag P1 | 500 x 500 |
| | *E. damarana* and sand circles mixed* | Palmwag M1 | 500 x 500 |
| | *E. damarana* and sand circles mixed* | Palmwag M2 | 500 x 500 |
| Brandberg | Fairy circles | Brandberg C1 | 500 x 500 |
| | Fairy circles | Brandberg C2 | 500 x 500 |
| | *E. damarana* | Brandberg P1 | 500 x 500 |
| | *E. damarana* | Brandberg P2 | 500 x 500 |
| | Fairy circles and *E. damarana* mixed | Brandberg M1 | 500 x 500 |
| | Fairy circles and *E. damarana* mixed | Brandberg M2 | 500 x 500 |
| Garub | Fairy circles | Garub C1 | 500 x 500 |
| | Fairy circles | Garub C2 | 500 x 500 |
| | *E. gummifera* | Garub P1 | 300 x 300 |
| | *E. gummifera* | Garub P2 | 300 x 300 |
| | Fairy circles and *E. gummifera* mixed | Garub M1 | 300 x 300 |
| | Fairy circles and *E. gummifera* mixed | Garub M2 | 300 x 300 |

* Sand circles in rocky areas formed by the lack of rocks in bare circular patches [6]

**Table S7.** Satellite data was obtained from WorldView-1 (WV-I) and WorldView-2 (WV-II) satellites, courtesy of the DigitalGlobe Foundation

| Satellite | Spectral resolution | Spatial resolution | Date | Site |
|---|---|---|---|---|
| WV-I | Panchromatic | 0.5 m | 2011 | Garub |
| | Multispectral | 1.5 m | 2012 | Garub |
| WV-II | Panchromatic | 0.5 m | 2011 | Giribes |
| | Multispectral | 1.5 m | 2012 | Brandberg, Palmwag |

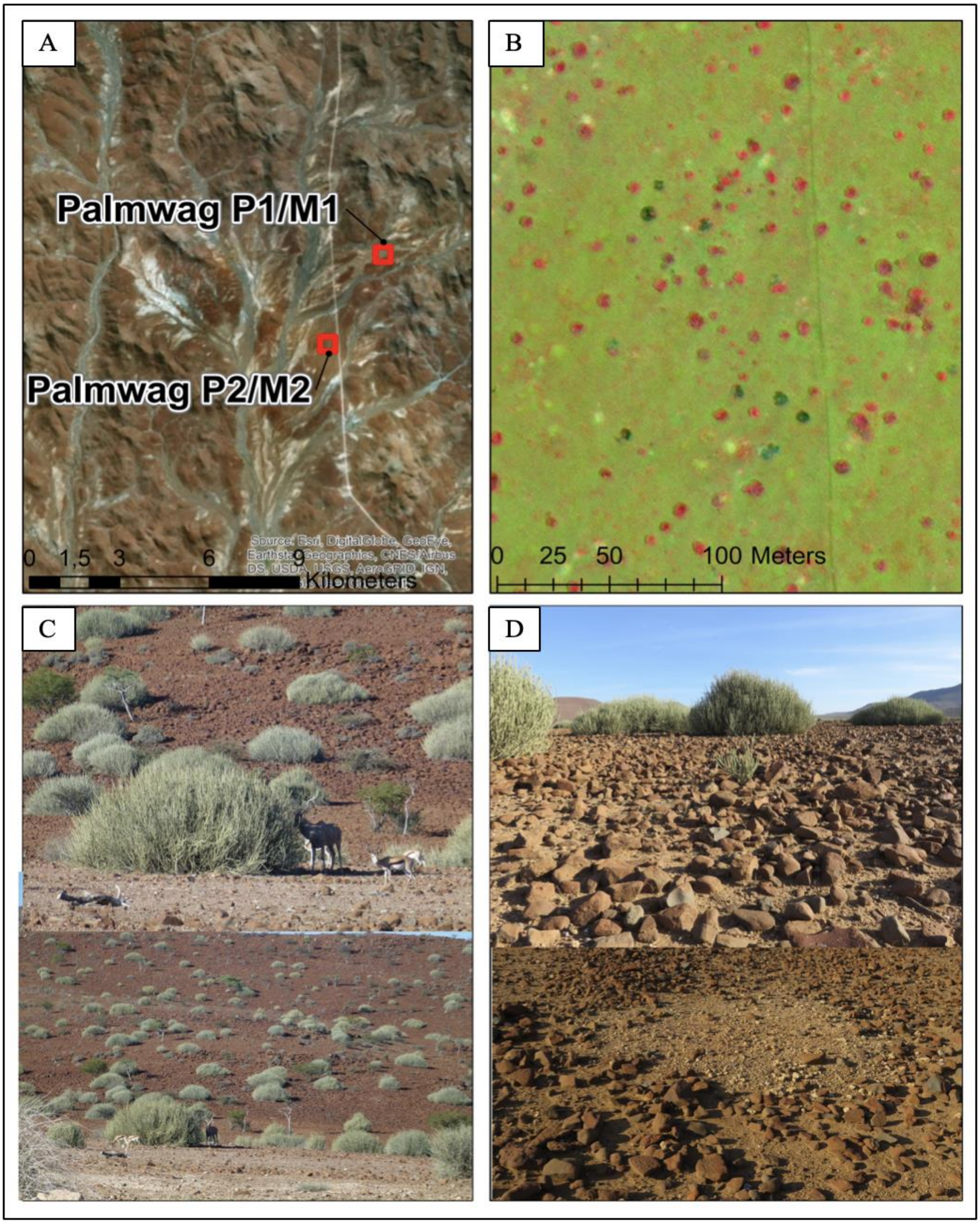


**Fig. S15.** Sampling design at Palmwag (red squares in **A**, source Google Earth™). *E. damarana* (red spots) and sand circles in rocks [6] (white spots) (**B**, courtesy of DigitalGlobe Foundation). Bright red indicates healthy vegetation, while dark tones indicate dead vegetation and green indicates the desert pavement. *E. damarana* occurring on the desert pavement along with sand circles in rocks (**C**, **D**)

*11.3. Brandberg*

Brandberg is located approximately 140 km south-southeast of Palmwag in the central-western plains at 21°04'26.85"S; 14°38'48.37"E, near the town of Uis (Fig. S16). On the sandy plains near Brandberg, *E. damarana* co-occurs with FCs and also without FCs on higher-lying, rocky areas. It was observed that many of the plants in the mixed sandy areas were dead. The environment surrounding Brandberg contains a combination of gravel and sandy plains, several smaller riffs and hills, as well as the Ugab River and several of its major tributaries (Fig. S16*A*). Both FCs and *E. damarana* frequently occur along drainage lines, where the shapes of the FCs are elliptical, with the longest axis parallel to the drainage lines (Fig. S16*C*), as was seen on the Giribes Plain. Fig. S16*B*–*D* show FCs co-occurring with *E. damarana*, both alive and dead plants can be seen, also evident from the false colour image composite (band 7, 4, 2), where a bright red tone indicates healthy vegetation and dark tones indicate dead vegetation.

Field work consisted of verification of the objects being studied remotely. This was done by recording the coordinates of 10 FCs and 10 *E. damarana* plants on the satellite images and locating them at Brandberg. During field work each object was verified. The occurrence of FCs at this site covers a much larger area than on the Giribes Plain and this was verified and photographed from an aeroplane.

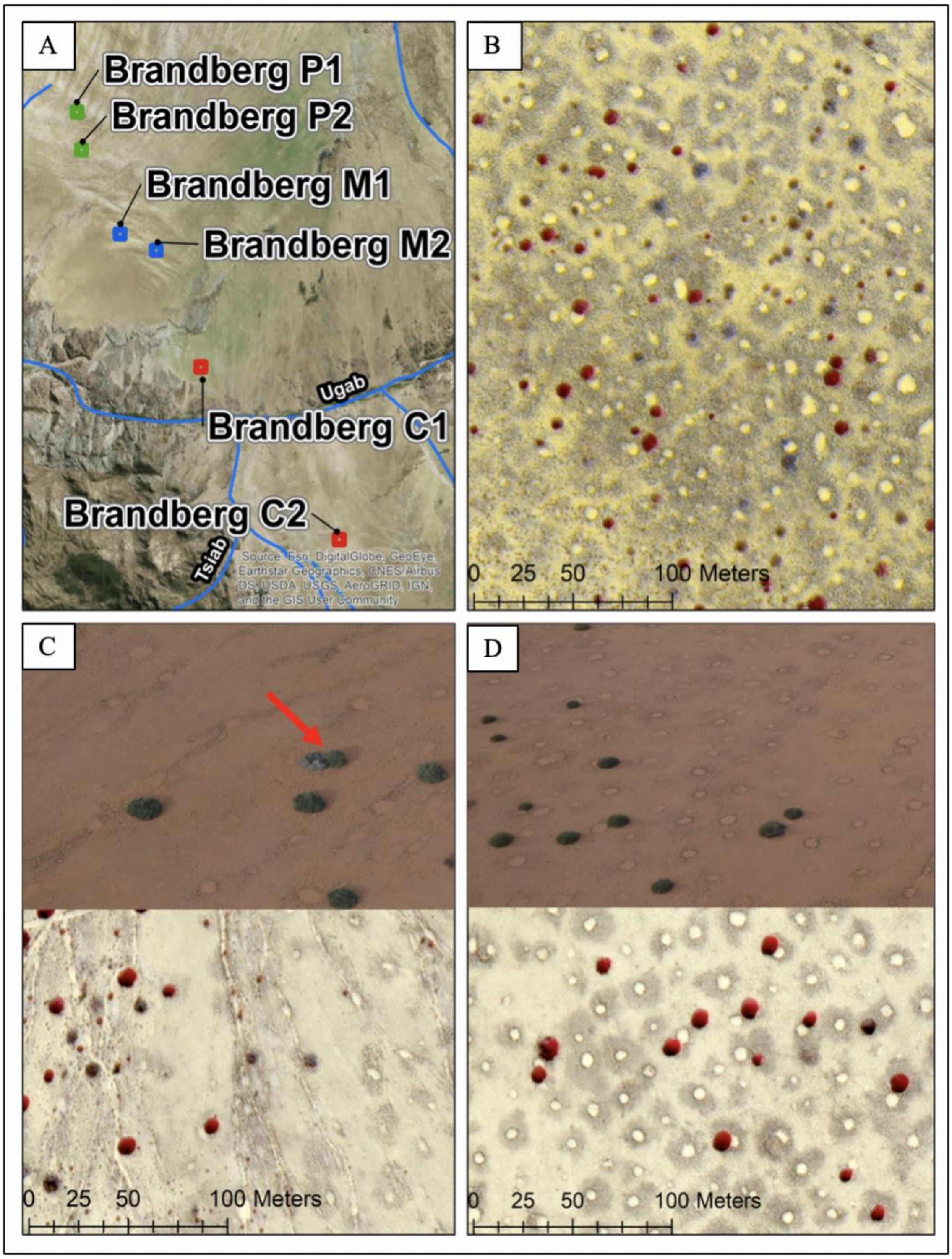


**Fig. S16.** Sampling design at Brandberg (**A**, source Google Earth™). The red squares indicate the two FC sites, the green squares indicate the two *E. damarana* sites and the blue squares indicate the two mixed sites. Fairy circles co-occurring with *E. damarana*, bright red indicates healthy vegetation, while dark tones indicate dead vegetation (**B–D**). Two adjacent *E. damarana* plants that will result in a 'mega FC' (**C**, red arrow). Satellite imagery in B–D courtesy of DigitalGlobe Foundation

*11.4. Garub*

Garub is located in southwestern Namibia at 26°38'52.17"S; 16°05'5.95"E, approximately 630 km south-southeast of Brandberg, between the towns of Aus and Lüderitz (Fig. S17). At this site, *E. gummifera* occurs alongside FCs on low-sloping sand dunes, where large numbers of these plants are dead and also on rocky/gravel areas in healthy populations without any FCs present. The environment consists of several rocky outcrops that rise above the flat terrain with various gravel plains and sandy deposits/accumulations. Low-sloping sand dunes have developed on the lower portions of many of the inselbergs (individual mountains), while a non-mobile dune field has developed between the inselbergs. The area is covered by grass from the genus *Stipagrostis*. Fairy circles and *E. gummifera* occur on these sandy deposits and also extend north of the dune fields onto gravel plains. The FC sites were 500 x 500 m, while the *E. gummifera* and the mixed sites were 300 x 300 m because of the terrain. In Fig. S17*C* FCs of Garub can be seen co-occurring with *E. gummifera*, which are also evident on the satellite image where a bright red tone indicates healthy vegetation and dark tones indicate dead vegetation. In Fig. S17*D* are photographs of FCs co-occurring with *E. gummifera*, many of which are dead and in different stages of decay.

Similar to the previous field sites, field work consisted of verification of the objects being studied remotely. This was done by recording the coordinates of 10 FCs and 10 *E. gummifera* plants on the satellite images and locating them at Garub. During field work each object was verified.

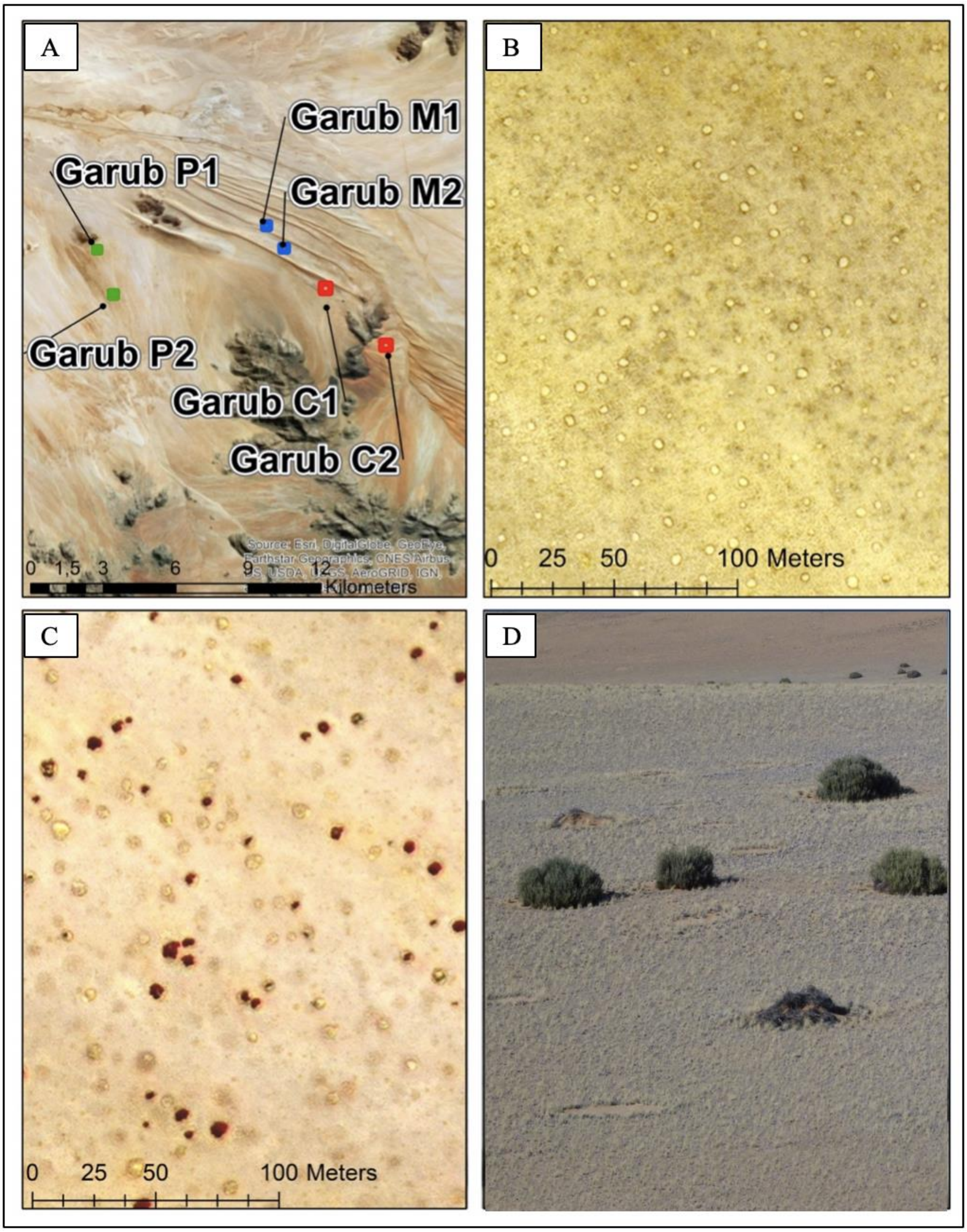


**Fig. S17.** Sampling design at Garub, southwestern Namibia (**A**, source Google Earth™). The red squares indicate the two FC sites, the green squares indicate the two *E. gummifera* sites and the blue squares indicate the two mixed sites. Fairy circles at Garub (**B**). Fairy circles co-occurring with *E. gummifera* (**C, D**), bright red indicates healthy vegetation, while dark tones indicate dead vegetation. Satellite imagery in B and C courtesy of DigitalGlobe Foundation

## 12. Spatial analysis – Clark-Evans test

The nearest event in the pattern to each event can be calculated as follows [7]:

$$R = \frac{\bar{r}_A}{\bar{r}_E}$$

where R = the nearest neighbour index;

$\bar{r}_A$ = average distance from randomly selected plants to their nearest neighbours;

$\bar{r}_E$ = expected mean distance between nearest neighbours.

Under the Poisson distribution with intensity $\lambda$, we have:

$$\bar{r}_E = \frac{1}{2\sqrt{\lambda}}$$

## 13. Spatial analysis – pair correlation function

Several second-order statistics, such as the commonly used Ripley's K-function or the pair-correlation function, use the information on all inter-point distances [8]. The K-function is the expected number of points in a circle of radius r, centred at an arbitrary point, divided by the intensity of the pattern. The pair correlation function g(r) results if the circles of Ripley's K-function are replaced by rings [9]. The pair correlation function is based on the distribution of distances of pairs of points and is a powerful tool that can be used to describe the second order structure of a spatial point pattern, i.e. the small-scale spatial correlation structure of the point pattern [8]. To reveal the degree of smaller-scale order effects in the pattern of FCs, *E. damarana* and *E. gummifera*, the pair-correlation function g(r) was used. This function measures the expected density of points at a given distance r of an arbitrary point, divided by the intensity, λ of the pattern. The pair correlation function enables an analyst to get a clearer picture of any separation distances at which there are few or many pairs of events and can be calculated with the following formula [9]:

$$g(r) = \frac{1}{\lambda^2} p(r)$$

Under complete spatial randomness (CSR) $g(r) = 1$. Values of $g(r) < 1$ indicate regularity (or also called overdispersion), while values of $g(r) > 1$ indicate aggregation (or clustering).

## 14. Spatial analysis – L-function

The L-Function is the cumulative counterpart of the pair correlation function [8]. The L-function makes visual interpretation of the K-function easier and results from a square root transformation of the K-function [8]. The L-function was used to assess departures from CSR at larger distances and calculated with the formula:

$$(L) = r(\frac{\sqrt{K(r)}}{\pi} - 1)$$

L(r) = 0 indicates CSR; L(r) < 0 indicates regularity (or overdispersion); L(r) > 0 indicates aggregation (or clustering).

All the summary statistics will be assessed in this study against CSR using the 5th highest and lowest values of 199 Monte Carlo simulations in order to produce 95% simulation envelopes.

## 15. Prediction model – data acquisition

**Table S8.** Data source specifications.

| **Data set** | **Format** | **Coordinate system** | **Source** |
|---|---|---|---|
| Average annual precipitation | Vector – (shapefile - polygon) | WGS 1984 | Environmental Information Services – Namibia [10] |
| Altitude | Raster – (41 x DEMs) Resolution: 30 x 30 m | UTM Zone 33 S | United States Geological Survey website [11] |
| Land cover | Raster – (20 x Satellite images) Resolution: 30 x 30 m | UTM Zone 33 S | United States Geological Survey website [11] |

### *15.1. The Atlas of Namibia*

The rainfall data used in the analysis was obtained from the Atlas of Namibia [12]. The rainfall data set was available in vector shapefile and contained categorical data. The scale of the data set was at the sub-continental scale. The rainfall data only had the WGS1984 geographic coordinate system associated with it and was projected to the same projection as the altitude and land cover data sets (see below). The data set was developed from actual rainfall monitoring data obtained from a variety of sources including private and governmental organizations [12], in contrast to Cramer & Barger [13] that used MAP generated from WorldClim data in their prediction model. For more details regarding the development of the rainfall data set see the Namibia Resource Consultants [14].

*15.2. ASTER satellite*

Elevation data was obtained from the United States Geological Survey's (USGS) website in the form of several ASTER Digital Elevation Models (DEMs) developed from the ASTER satellite [11]. A total of 41 DEMs were downloaded from the USGS website through the Earth Explorer interface. The spatial reference of the DEMs was UTM Zone 33S, which is based on the WGS84 datum. The spatial resolution of DEMs was 30 x 30 m.

*15.3. Landsat satellite*

It was decided to use Landsat™ 5 imagery, which is geometrically, radiometrically and atmospherically corrected. This decision was based on the fact that in 2003 the scan line corrector (SLC) of the Landsat™ 7 satellite failed. This resulted in data gaps, leaving individual scenes with only 78 percent of their pixels [11]. The spatial reference associated with the Landsat scenes were identical to the ASTER satellite scenes, i.e. UTM Zone 33S. A total of 20 Landsat 5 satellite images were obtained for March to May 2009. If an image was not available for 2009, one from 2008 was selected. The search criteria used to obtain the imagery was set to only include imagery with 0% cloud cover. The image dimensions were 170 km north-south by 183 km east-west, with a resolution of 30 x 30 m. Considerable processing of satellite imagery was required to extract the land cover data and is discussed in the model building steps below.

Landsat scenes were compiled by the USGS in such a way that the sides of adjacent images overlap. This could result in processing errors and had to be prevented by clipping the images. Clipping is a raster processing tool that is used to reduce the spatial extent of the image. This was done by creating a new polygon feature class that defined the reduced spatial extent of the image. This effectively means that the side of the image that overlapped with an adjacent image was 'cut-off'. The newly created feature classes were in the shape of a square and the position of each square was determined by the previous square's position.

In order to extract the desired land cover, i.e. sandy plains covered by grasslands, the images had to be classified into different land cover types according to their spectral properties. The *ISO-Cluster Unsupervised Image Classification* technique was applied to each image. This image classification technique entails grouping pixels with similar spectral values together into a group. Each image was grouped into 15 land cover classes representing features on the surface of the earth. It must be noted that surface features were represented by more than one land cover type; i.e. bare rock surfaces could be represented by more than two classes. Rock surfaces typically included inselbergs, rocky outcrops, and desert pavement (coarse gravel plains).

The raw land cover classification data was manually inspected and classes representing the same surface features grouped into a single land cover class so that the different land cover types corresponded to either rock, vegetation or sandy grass plains. Several measures were taken to correctly classify the land cover types according to the true surface type. This included using Google Earth™, the Landsat™ imagery, ArcMap World Imagery base, the National Land cover data set for Namibia [15] and shapefiles containing the location of various sandy accumulations [12] as reference to identify different features. The areas in Google Earth™ corresponding to the different images were visually assessed while assigning the land cover classes (Fig. S18). The Landsat imagery, ArcMap base map and the National Land cover data set layers were placed underneath the raw image classification data and the 'slider' tool was used to view the underlying data.

These processed land cover types were then reclassified so that the grasslands were contained in one land cover type and all other classes in one. This was done by assigning a value of '1' to the identified grassland land cover type and assigning a value of '0' to all the non-grassland land cover classes. The 'extract by attribute' tool was used to isolate the grasslands only and exclude all the other land cover classes. These 22 grassland raster data sets were then combined into a single layer with the 'mosaic to new data set' tool and represented the grasslands identified for the study area.

During the land cover classification process, it was observed that the land cover type 'bare rock' was the most easily distinguishable from the other classes. The 'vegetation class' was also easily identified, and consisted of more lush vegetation, for example thick vegetation found along rivers and dense bush. However, separating gravel plains from sandy plains proved the most difficult. Desert pavements, a type of gravel plain, for example those characteristic of the Etandeka Plateau, was included under bare rock. During field work, the location of several desert pavements was recorded along the route travelled. This aided in correctly identifying this land cover type. Less coarse gravel plains were also observed; these gravel plains are often covered by thin layers of sand. Fairy circles and *E. gummifera* co-occur on these gravel plains near Garub. Additionally, Van der Walt et al. [16] studied FCs found on gravel plains north of the Kuiseb River. As the satellite images were obtained after years of good rainfall, the abundant grass cover made it more difficult to distinguish between the less coarse gravel plains and the sandy plains. Therefore, these 'less coarse gravel plains' were included under the sandy grass plains class. As a result, large areas south of Brandberg and north of the Kuiseb River (the central-western plains), were included under the sandy grasslands layer (Fig. S18).

The final step in building the model was to do an intersection of the 'ideal rainfall and altitude' and the grassy covered sandy plains. This was done by clipping the land cover data sets to the ideal rainfall and altitude layer. This resulted in a final site suitability map indicating areas that are predicted to have FCs in mainly Namibia.

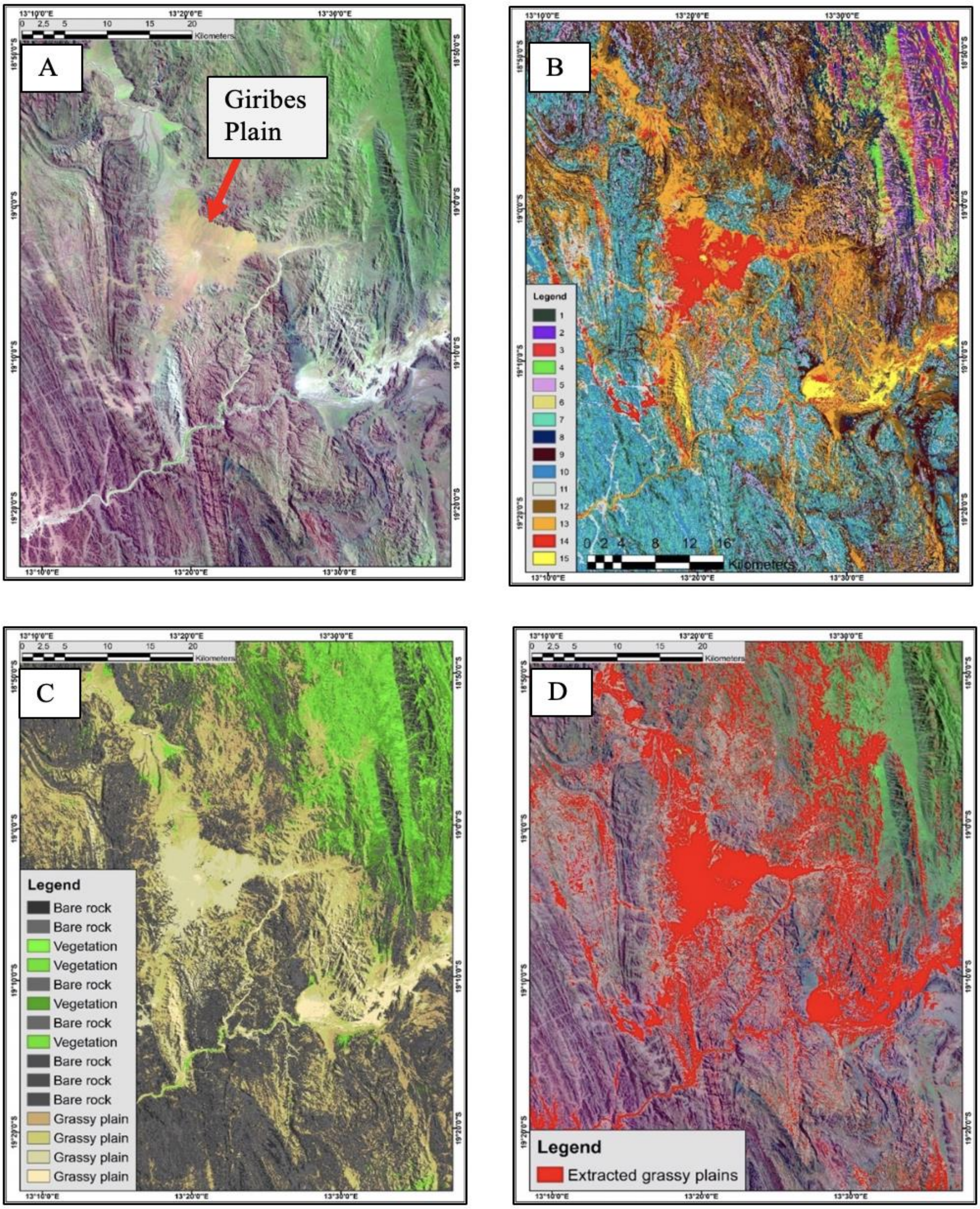


**Fig. S18.** The land cover classification process is illustrated by using the greater Giribes area as an example. Part of a Landsat™ 5 false colour composite image of which bands 7, 4, 2 were used to construct the image (**A**). The resultant 15 class ISO-Cluster image classification before manual inspection of the raw classified data (**B**). The re-classified ISO Cluster classified image (**C**). The grasslands on sandy soil extracted from the image classification process are shown in red (**D**). Created with ArcMap

## 16. Creating the prediction model

FCs are known to occur in areas with MAP of between 50–150 mm per year. In order to extract this data a shapefile containing the MAP data for the study area was obtained. To ensure that all the data layers contained the same spatial reference, the shapefile containing the MAP was projected to the UTM Zone 33S projection. Next a Boolean operation was performed to isolate the 50–150 mm MAP for the study area. The steps in the creation of the ArcGIS model are described in (Fig. S19 and S20).

Fairy circles are known to occur in areas with an elevation range of between 500–1 200 m above mean sea level. In order to extract this data 42 ASTER DEM's were imported into ArcMap and mosaicked into a single layer. This data set was then reclassified to contain areas with an elevation between 500 and 1 200 mamsl by assigning a '1' to elevations within this range and a '0' to elevations outside of this range (both less and greater than the ideal altitudinal range). This layer was then converted to a vector data set. The areas where both the 'suitable' rainfall and the 'suitable' altitude overlap was identified using the intersection tool in ArcMap.

The next step in the building of the model, was to extract areas within the ideal rainfall and altitudinal range where the underlying land cover are sandy plains covered by grasslands. This involved using the Landsat 5 imagery to extract the pertinent criteria (i.e. sandy plains covered by grasslands). A false colour image (B = 7, 4, 2) was created for each Landsat scene.

## 17. Prediction model validation – random points

The second part of the validation process was to determine if areas where FCs were predicted to be absent did not contain FCs, using the identified criteria (negative control). The resultant area, 29 380 $km^2$, was smaller than the modelled FC distribution of 55 955 $km^2$. The total area covered by the 100 random points, used to inspect the modelled FC distribution (positive control), was 31 500 $km^2$. To inspect an area within the negative control of the relative same size as that of the modelled FC distribution, 83 random points were generated with an 8 km buffer drawn around each point resulting in a total area of 16 600 $km^2$. This resulted in an area of 200 $km^2$ per point, compared to 315 $km^2$ of the 100 positive control random sample points.

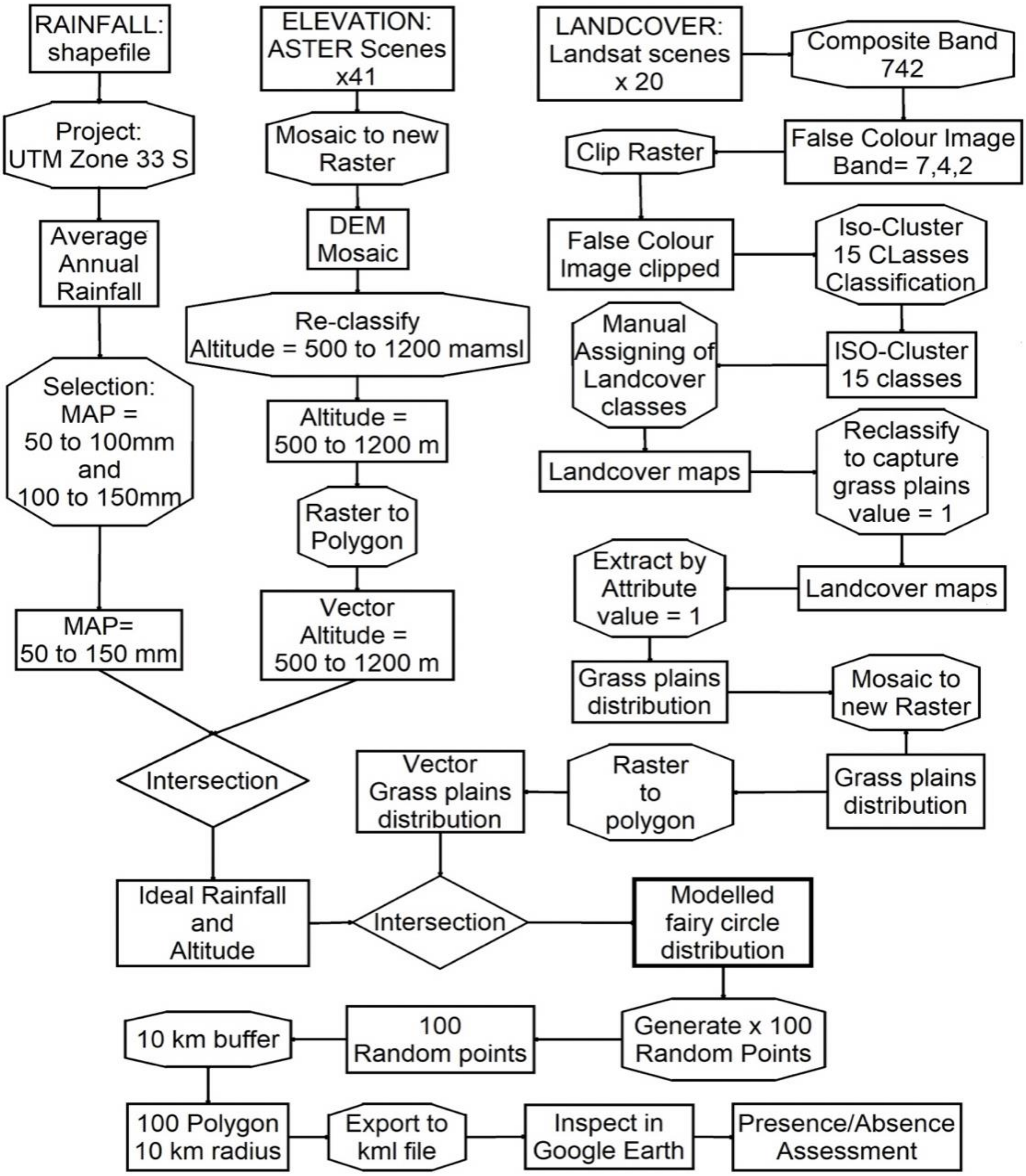


**Fig. S19.** Steps in the process to create the GIS model for the prediction of where FCs will be formed and the generation of random points for the positive validation where FCs should be present

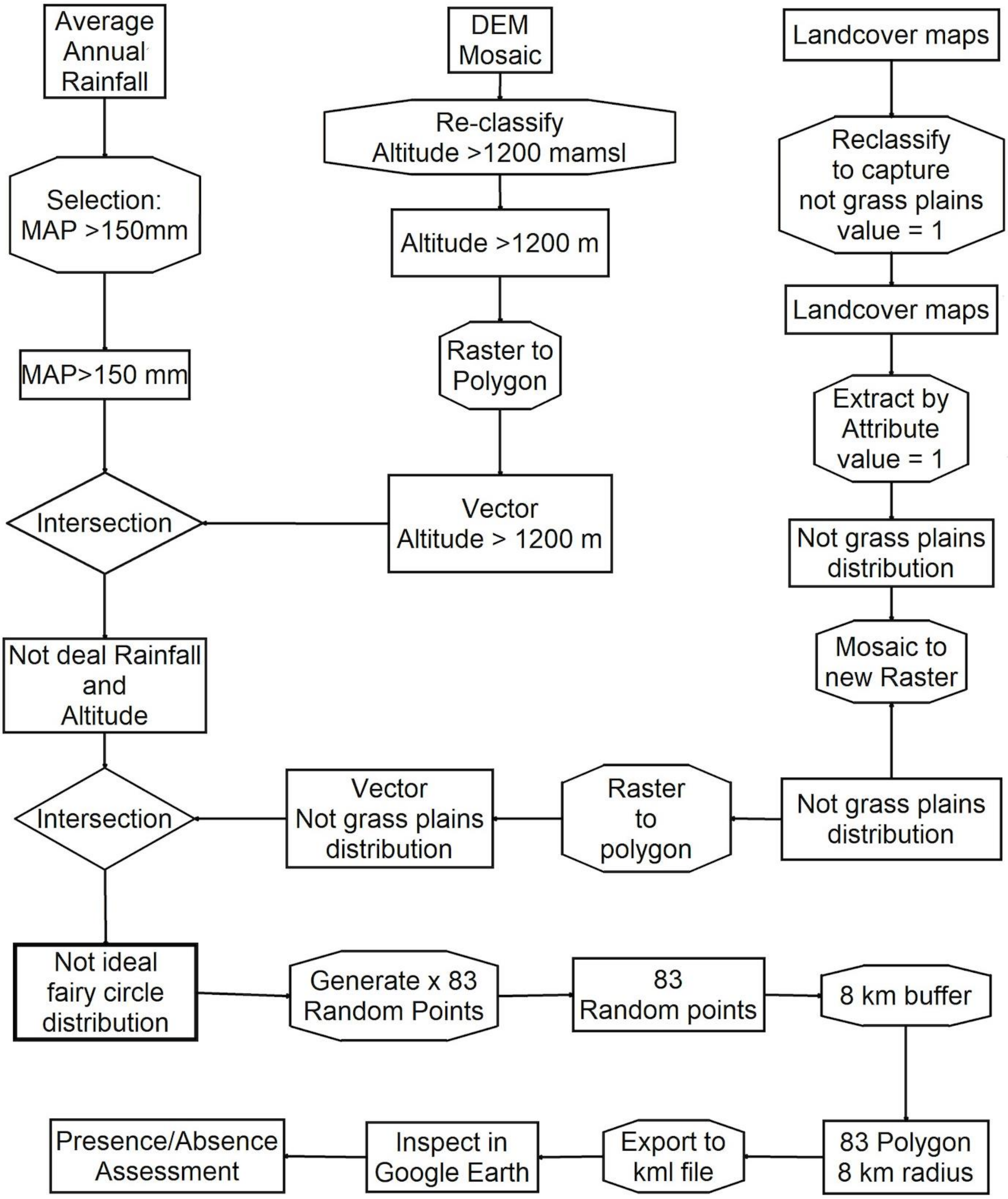


**Fig. S20.** Steps in the process for the generation of random points for the negative control where FCs should be absent

## Additional references


1. Doerr SH. Short communication on standardizing the ‘water drop penetration time’ and the ‘molarity of an ethanol droplet’ techniques to classify soil hydrophobicity: A case study using medium textured soils. Earth Surf Process. 1998;23:663–668.
2. Bowman G, Hutka J, Particle size analysis. In: Soil Physical Measurement and Interpretation for land evaluation. Victoria: CSIRO Publishing; 2002.
3. Patel JB. 16S rRNA Gene Sequencing for bacterial pathogen identification in the clinical laboratory. Mol Diagn. 2001;6:313–321.
4. Hall TA. BioEdit: A user friendly biological sequence alignment editor and analysis program for Windows 95/98/NT. Nucleic Acids Symp Ser. 1999;41:95–98.
5. Wood YA, Graham RC, Wells SG. Surface mosaic map unit development for a desert pavement surface. J Arid Environ. 2002;52:305–317.
6. Meyer JJM, Meyer AC, Meyer NL. Sand circles in stony landscapes of Namibia are caused by large *Euphorbia* shrubs. S Afr J Bot 2020;130;25–29; Available from: https://doi.org/10.1016/j.sajb.2019.11.009.
7. Clark PJ, Evans FC. Distance to nearest neighbor as a measure of spatial relationships in populations. Ecology. 1954;35:445–453.
8. Wiegand T, Gunatilleke S, Gunatilleke N, Okuda T. Analyzing the spatial structure of a Sri Lankan tree species with multiple scales of clustering. Ecology. 2007;(88)12: 3088–3102.
9. Law R, Illian J, Burslem D, Gratzer G, Gunatilleke C, Gunatilleke I. Ecological information from spatial patterns of plants: insights from point process theory. J Ecol. 2009;97:616–628; Available from: https://doi.org/10.1111/j.1365-2745.2009.01510.x.
10. Environmental Information Service (EIS) – Namibia. 2018. http://www.the-eis.com. Accessed 12 Sept 2019.
11. United States Geological Survey (USGS) 2018. http://landsat.usgs.gov. Accessed 12 Sept 2019.
12. Mendelsohn J, Jarvis A, Roberts C, Robertson T. Atlas of Namibia. Cape Town: Ministry of Environment & Tourism. David Philip; 2002.
13. Cramer MD, Barger NN. Are Namibian ‘Fairy Circles’ the consequence of self-organizing spatial vegetation patterning? PLoS ONE. 2013;8(8):e70876; Available from: https://doi.org/10.1371/journal.pone.0070876.

14. Namibia Resource Consultants. Rainfall distribution in Namibia: data analysis and mapping of spatial, temporal, and Southern Oscillation Index aspects. Phase Report for the Ministry of Agriculture, Water and Rural Development, Namibia; 1999.
15. FAO (2010). Land cover of Namibia – Globalcover Regional. 2009-05-12 T15:49; Available from: http://www.fao.org/geonetwork/srv/en/main.home.
16. Van der Walt AJ, Riegardt MJ, Cowan DA, Seely M, Ramond J-B. Unique microbial phylotypes in Namib desert dune and gravel plain fairy circle soils. Appl Environ Microbiol. 2016;82: 4592–4601; Available from: https://doi.org/10.1128/AEM.00844-16.
